# Programmable Helicity and Macrocycle Symmetry in β‑Peptides via Site-Selective Thioamide Substitution

**DOI:** 10.1021/jacs.5c13858

**Published:** 2025-10-29

**Authors:** Jungwoo Hong, Jaewook Kim, Jintaek Gong, Seoneun Jeong, Yi Sak Park, Sung Hyun Yoo, Jin Kim, Hee-Seung Lee

**Affiliations:** † Department of Chemistry, 34968 Korea Advanced Institute of Science and Technology (KAIST), Daejeon 34141, Republic of Korea; ‡ InnoCORE AI Co-Research & Education for Innovative Drug (AI-CRED) Institute, KAIST, Daejeon 34141, Republic of Korea; § Department of Chemistry, 34931 Chonnam National University, Gwangju 61186, Republic of Korea; ∥ Department of Chemistry, 65380 Sunchon National University, Sunchon 57922, Republic of Korea

## Abstract

Thioamides—minimalist amide isosteres in which sulfur replaces the backbone carbonyl oxygen—offer a precise means to modulate peptide conformation through altered hydrogen-bond geometry and polarity. This work presents a general and experimentally validated strategy for programming β-peptide secondary structure with atomic precision, using site-selective thioamide substitution as a minimalist backbone modification. While thioamides have been studied individually, their positional control within β-peptides to direct helicity, curvature, and topology has not been achieved before. Using *trans*-2-aminocyclopentanecarboxylic acid (ACPC) foldamers as a model system, we show that strategic thioamide placement enables hybrid 12/8-helices, backbone-encoded curvature, and conical 16/12-helices; symmetry-defined macrocycles (pseudo-*C*_2_, pseudo-*C*_3_, pseudo-*C*_4_) inaccessible by conventional β-peptide synthesis; gram-scale, solution-phase synthesis of β-peptides up to 32-mers (>4 kDa), the longest reported to date; and orthogonal editing via mild Ag­(I)-mediated backbone conversion to all-amide analogs in 97–99% yield, allowing folding to be programmed with temporary thioamide units before conversion to the desired scaffold. These advances establish a unified framework for controlling β-peptide helicity and topology through minimal backbone editing, significantly expanding the accessible structural and functional space for foldamer chemistry. The concepts and methodologies are broadly applicable to organic synthesis, supramolecular chemistry, biomolecular engineering, and peptide-inspired materials.

## Graphical abstract

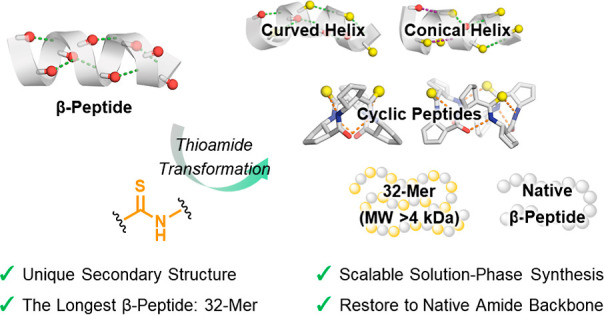

## Introduction

Precise control of secondary structure remains a fundamental challenge in peptide design, with broad implications for biomolecular engineering, materials development, and catalysis.^1–5^ One promising yet underexplored strategy involves the use of thioamides—minimalist amide isosteres in which sulfur replaces the backbone carbonyl oxygen.^6–11^ Although the O → S-substitution is chemically subtle, the distinct physicochemical properties of sulfur—including its larger atomic radius and reduced electronegativity—significantly alter hydrogen bonding behavior, polarity, and conformational preferences.^12–19^ These features have inspired growing interest in thioamides as backbone engineering elements in peptide design.

Thioamides offer a unique balance of hydrogen bond-donating and accepting properties.^20–22^ The increased acidity of the N–H group enhances its donor strength, while the diminished acceptor capacity of the C=S moiety subtly disrupts canonical intramolecular hydrogen bonding patterns.^23–26^ Beyond hydrogen bonding, thioamides are chemically versatile and enable diverse transformations, yet their application in rational peptide design remains limited.^27–31^ In particular, systematic studies on how thioamides modulate peptide conformation, especially in multithioamide systems, are scarce.^32–34^ This gap has hindered their integration as structural editing tools in foldamer or scaffold development. Notably, recent studies have begun to explore thioamide incorporation in α-helices and β-hairpins for modulating stability, proteolytic resistance, and spectroscopic properties, but analogous strategies in β-peptide foldamers remain rare.

To address this limitation, we explored thioamide incorporation into β-peptides composed of *trans*-2-aminocyclopentanecarboxylic acid (ACPC), a conformationally constrained building block known to stabilize 12-helical structures.^35–37^ Compared to α-peptides, β-peptides exhibit superior resistance to degradation, reduced epimerization, and predictable folding propensities, making them an ideal platform for evaluating subtle backbone modifications.^38–42^ Using ACPC-based oligomers, we systematically examined how thioamide placement affects backbone conformation through hydrogen bond modulation.

Our study reveals that site-selective thioamide substitution enables programmable control over β-peptide helicity and symmetry, producing noncanonical architectures such as curved and conical helices as well as macrocycles with defined pseudo-*C*_2_, pseudo-*C*_3_, and pseudo-*C*_4_ symmetries. These structural outcomes were characterized using X-ray crystallography and solution-state nuclear magnetic resonance (NMR) spectroscopy. Enhanced solubility of thioamide-containing peptides further allowed a modular, solution-phase synthesis of long β-peptides up to 32-mers, circumventing the need for solid-phase synthesis. We also demonstrated a mild, silver­(I)-mediated oxidative desulfurization to regenerate native amide backbones, establishing a postsynthetic backbone editing strategy.

Together, these results position thioamides as versatile motifs for conformational programming and synthetic access to complex peptide architectures, expanding the design toolkit for foldamers, biomimetic materials, and modular scaffolds. Beyond fundamental structure control, the ability to integrate helicity programming with macrocycle topology and scalable synthesis offers promising opportunities for chiral recognition, molecular channel engineering, and foldamer-based therapeutic design. A schematic overview of the thioamide-enabled design platform—encompassing site-selective substitution, solution-phase fragment condensation, and postsynthetic backbone transformation—is presented in Figure 1.

**Figure 1:**
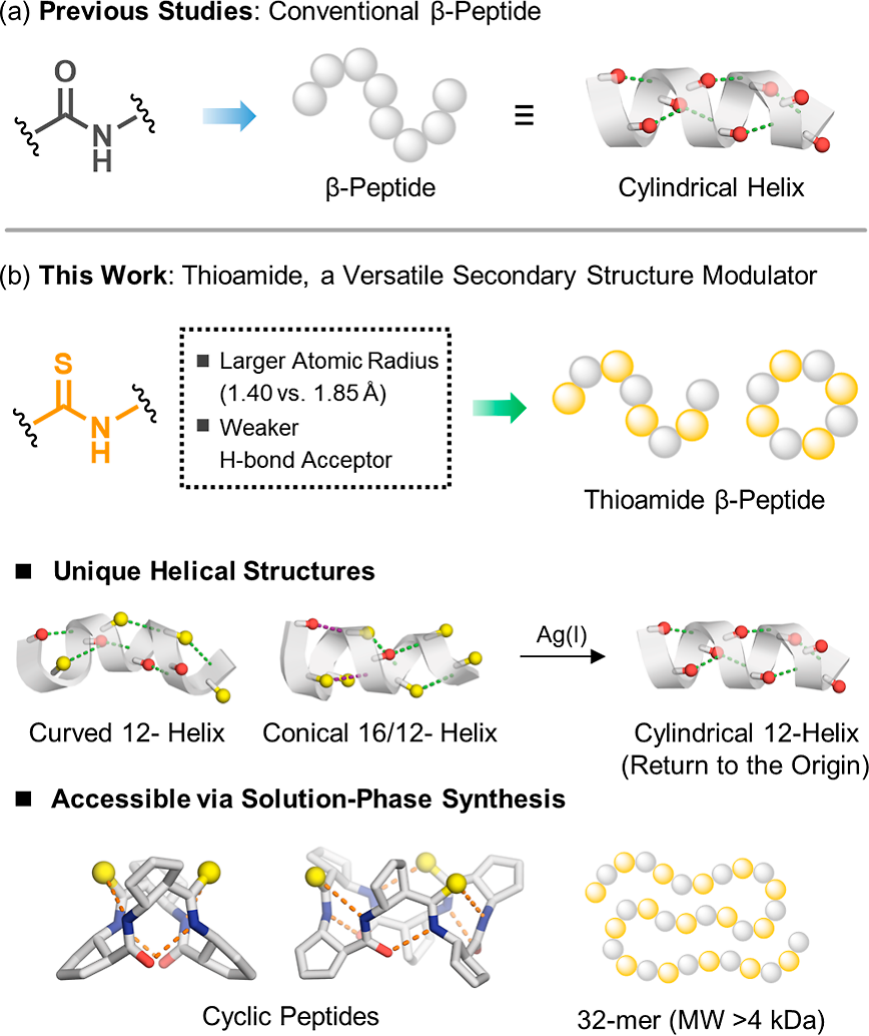
(a) Previous studies: canonical β-peptides with all-amide backbones adopt cylindrical 12-helices. (b) This work: site-selective O → Ssubstitution yields thioamide β-peptides, where the larger atomic radius and weaker hydrogen-bond acceptor property of sulfur enable programmable secondary-structure modulation. This approach provides access to curved and conical helices, symmetry-defined macrocycles, and long β-peptides (up to 32-mers, MW > 4 kDa) via solution-phase synthesis.

## Results and Discussion

To investigate how site-selective thioamide substitution influences intramolecular hydrogen bonding and helix formation, we designed a series of β-peptides based on (*S*,*S*)-ACPC oligomers, introducing thioamide groups at distinct positions—*C*-terminal (**1b**), throughout the backbone (**1c**), *N*-terminal (**1d**), and in alternating positions (**1e**, **2b**)—using ACPC_4_ (**1a**) and ACPC_6_ (**2a**) as all-amide references (Figure 2a). This positional variation allowed us to directly assess how local O → Ssubstitution perturbs hydrogen-bonding patterns and overall helicity without changing side-chain composition or overall sequence length. Such a design isolates the effect of the thioamide itself, enabling a structure–property correlation that is difficult to achieve with side-chain modifications or global sequence changes. All β-peptides were protected with *N*-terminal Boc and *C*-terminal Bn groups and synthesized by conventional solution-phase peptide coupling, mainly using PyBOP (EDCI·HCl was employed only for selected nonthioamide couplings). Thioamide residues were introduced through chemoselective thionation with Lawesson’s reagent (see Supporting Information).

**Figure 2:**
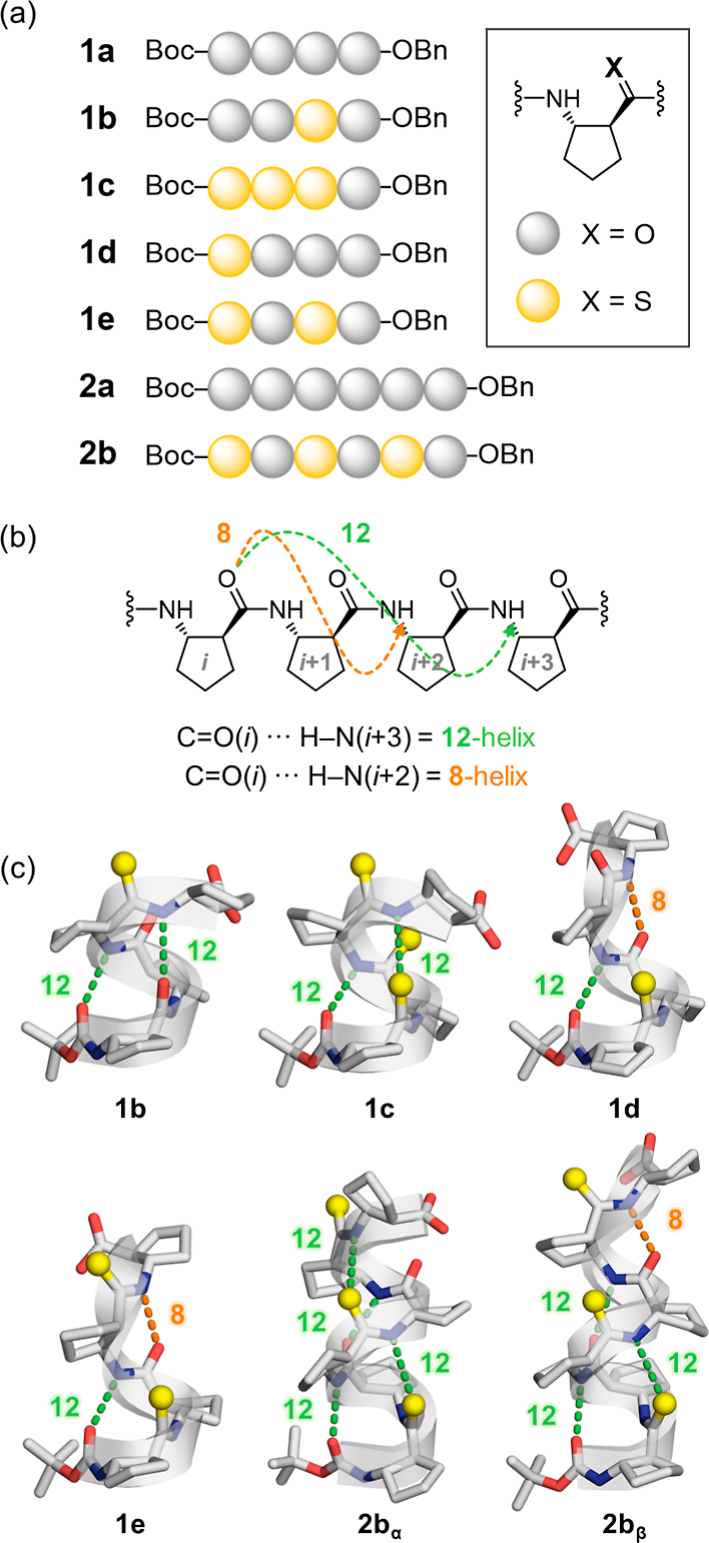
(a) Schematic structures of the thioamide β-peptide series (**1b**–**1e** and **2b**), derived from ACPC oligomers and protected with Boc and Bn groups at the *N*- and *C*-termini, respectively. (b) Representative hydrogen-bonding patterns stabilizing 12-membered (green) and 8-membered (orange) β-peptide helices. (c) Single-crystal X-ray structures of the thioamide-substituted peptides, showing position-dependent helicity. **1d** and **1e** adopt hybrid 12/8-helices. Sulfur atoms are shown in yellow. Solvent molecules, *C*-terminal Bn groups, and disordered regions are omitted for clarity. The structure of **1e** represents one conformer from the asymmetric unit.

Single-crystal X-ray diffraction (XRD) revealed that the parent foldamers **1a** and **2a** adopt canonical 12-helical conformations stabilized by C=O­(*i*)···H–N­(*i*+3) hydrogen bonds (green arrows, Figure 2b).^43,44^ Consistent with previous crystallographic studies of ACPC oligomers, 8-helical motifs (orange arrows) were absent, reflecting the conformational constraint imposed by the cyclopentane ring. In contrast, the thioamide analogs displayed distinct position-dependent effects (Figure 2c). *C*-terminal (**1b**) and fully substituted (**1c**) sequences retained the 12-helix, suggesting that replacement of backbone carbonyls at these sites does not significantly disrupt the *i* → *i*+3 hydrogen-bonding network. By contrast, *N*-terminal thioamide–amide sequences (**1d**, **1e**) adopted hybrid 12/8-helices incorporating both 12-membered (green) and 8-membered (orange) hydrogen bonds. This deviation likely arises from the reduced hydrogen-bond acceptor strength of the *N*-terminal thioamide, which promotes compensatory hydrogen bonding from adjacent amide units to satisfy the backbone’s hydrogen-bonding potential.

In prior crystallographic studies, ACPC oligomers predominantly adopt 12-helical conformations, and mixed 12/8-helices have only been observed under specific perturbations such as metal coordination.^45^ To our knowledge, the *N*-terminal thioamide substitution reported in this work (Figures 2c, S1, Table S2) represents another example of such a perturbation in a homooligomeric ACPC backbone. The emergence of such hybrid helices in a homooligomeric ACPC backbone highlights the sensitivity of its folding pattern to subtle backbone modifications. These results establish that strategic placement of thioamides can modulate helix geometry in a predictable manner, stabilizing otherwise disfavored conformations even under torsional strain. The fact that such modulation is achieved without altering the side-chain pattern underscores the utility of thioamide substitution as a purely backbone-based design element.

Encouraged by the hybrid architectures in **1d** and **1e**, we examined the longer hexamer **2b** containing an alternating thioamide–amide motif. XRD analysis revealed two polymorphs, **2b**_**α**_ and **2b**_**β**_, both predominantly 12-helical, but with **2b**_**β**_ incorporating a single *C*-terminal 8-membered hydrogen bond—indicating that longer oligomers can partially accommodate 8-helical segments in the solid state. In solution, however, 2D NMR experiments—total correlation spectroscopy (TOCSY) and rotating-frame Overhauser effect spectroscopy (ROESY)—in pyridine-*d*_5_ showed no nuclear Overhauser effect (NOE) correlations such as H_β_(*i*) ↔ H_N_(*i*+2) or H_β_(*i*) ↔ H_α_(*i*+2) at the *C*-terminus (Figure S2), suggesting that the 12-helical hydrogen-bond network is not maintained at this terminus. These results indicate that thioamide–amide units can function as sequence-independent, programmable modules for secondary structure modulation in both short and extended β-peptides, and motivated us to explore whether cumulative thioamide substitutions could introduce additional noncanonical helical geometries.

To probe the cumulative effects of multiple thioamide units on β-peptide helicity, we synthesized the β-octapeptide ACPC_8_ (**3a**) and its thioamide analog **3b**, incorporating a repeating tetrapeptide motif analogous to **1e** (Figures 3a, S3, Table S3). XRD analysis confirmed that both foldamers adopt 12-helical conformations, indicating that the periodic placement of thioamides in **3b** does not disrupt the overall hydrogen-bonding pattern. However, a pronounced difference in global helix shape was evident: the all-amide foldamer **3a** formed a canonical cylindrical 12-helix, whereas **3b** exhibited a distinct curvature along the helical axis despite retaining the same hydrogen-bond topology.

**Figure 3:**
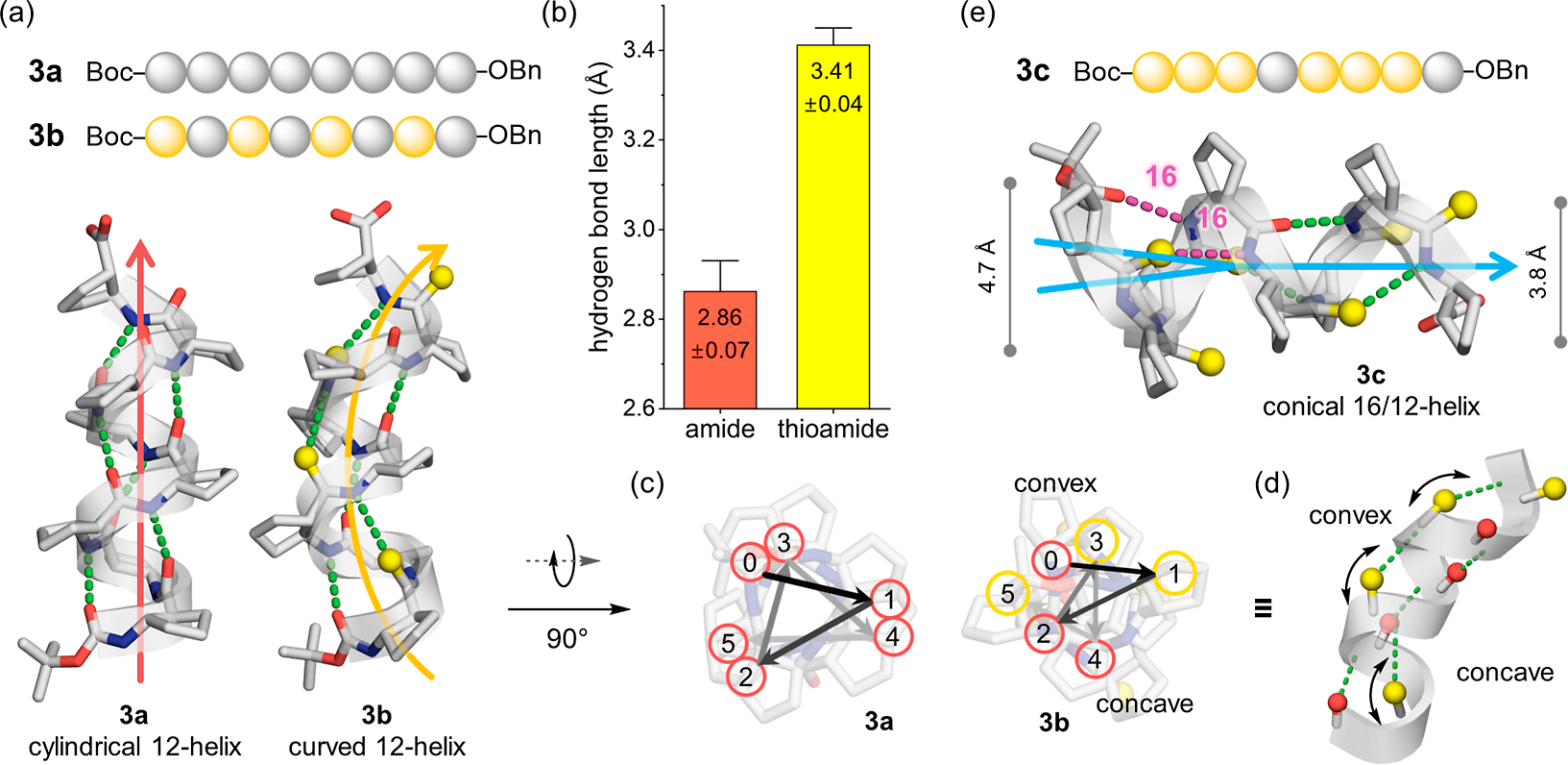
(a) Structures of β-octapeptides **3a** and **3b**, showing a transition from a cylindrical 12-helix (**3a**) to a curved 12-helix (**3b**) upon thioamide substitution. (b) Hydrogen-bond lengths (Å) for amide and thioamide acceptors in **3b**. (c) Axial views of **3a** and **3b** highlighting convex and concave faces arising from asymmetric thioamide placement. (d) Schematic representation of curvature in **3b**. (e) Structure of **3c** adopting a conical 16/12-helix, with diameters indicated for the 16- and 12-helical segments. Hydrogen bonds are shown as green (12-helix) or magenta (16-helix) dotted lines; sulfur atoms are depicted in yellow. Solvent molecules, *C*-terminal Bn groups, and disordered regions are omitted for clarity. The structure of **3c** represents one conformer from the asymmetric unit.

This curvature in **3b** correlates with the intrinsic geometric consequences of thioamide substitution. The donor–acceptor distances measured for hydrogen bonds mediated by C=O and C=S groups averaged 2.86 Å and 3.41 Å, respectively (Figure 3b, Table S4), consistent with the longer bond length and weaker acceptor capacity of the thioamide carbonyl. Axial projections of the crystal structures revealed an asymmetric distribution of C=S bonds around the helix circumference, producing one convex and one concave face (Figure 3c,d). The resulting curvature is intrinsic to the backbone architecture rather than induced by packing forces, as it is present in multiple independent molecules within the asymmetric unit.

Curved helices in β-peptides are uncommon, particularly in homooligomeric ACPC backbones, which typically favor cylindrical helices due to their rigid five-membered ring constraints. To our knowledge, the curvature observed here represents one of the few documented cases in an ACPC homooligomer, arising solely from backbone modification without external coordination or sequence heterogeneity. This demonstrates that thioamide substitution can be used not only to modulate hydrogen-bond patterns, as in **1d** and **1e**, but also to introduce directional bending into a helical scaffold in a predictable and programmable fashion.

Such programmable curvature has important implications for higher-order structure design.^46,47^ By controlling the magnitude and directionality of bending, it may be possible to assemble helices into defined bundles, arcs, or closed rings with tunable internal cavities.^48^ In protein–peptide recognition, curvature could be exploited to complement concave protein surfaces or to create chiral grooves for selective binding. Encouraged by this capability, we next sought to explore whether alternative thioamide placement patterns could induce other noncanonical helical geometries.

Inspired by the curvature observed in **3b**, we next designed and synthesized the thioamide-containing octamer **3c**, bearing a tetrameric motif analogous to **1c** (Figures 3e, S3, Table S4). Single-crystal XRD analysis revealed a unique hybrid 16/12-helical architecture, comprising both 16-membered C=O­(*i*)···H–N­(*i*+4) hydrogen bonds and 12-membered C=O­(*i*)···H–N­(*i*+3) hydrogen bonds. The 16-helix segment is less tightly wound than the 12-helix segment, resulting in a measurable increase in helix diameter (4.7 Å vs 3.8 Å) and a gradual tapering of the helix from the *N*-terminus to the *C*-terminus, giving rise to a conical morphology.

This hybrid 16/12-helical conformation is notable because ACPC-based β-peptides typically adopt exclusively 12-helical structures in the absence of external perturbations. The emergence of the 16-helical segment in **3c** suggests that thioamide substitution can redistribute hydrogen-bonding patterns along the backbone, relieving torsional strain and allowing the helix to locally unwind. The result is a shape-programmed scaffold in which helical pitch and diameter vary systematically along the molecular axis.

Conical helices of this type could offer distinct advantages in supramolecular assembly. The tapering geometry may facilitate directional packing, influence void space within assembled arrays, or enable selective encapsulation of guest molecules based on size gradients. Moreover, combining curvature (as in **3b**) with conical shaping (as in **3c**) could yield complex, hierarchically organized architectures with tailored spatial properties (Figures S4–S6). These findings demonstrate that by varying the position and periodicity of thioamide substitution, it is possible to exert multidimensional control over β-peptide helix geometry, extending beyond uniform curvature into more sophisticated, spatially graded structures.

Building on the structural programmability demonstrated in linear β-peptides, we next explored whether thioamide substitution could facilitate macrocyclization. Cyclic β-peptides are attractive scaffolds in molecular design because their constrained geometry enhances conformational stability, improves metabolic resistance, and creates well-defined cavities for molecular recognition.^49–51^ However, the efficient synthesis of β-peptide macrocycles can be hindered by poor solubility of the linear precursors, especially for sequences rich in hydrophobic residues or containing multiple ACPC units.

To evaluate the potential of thioamides in overcoming these limitations, we prepared heterochiral β-peptide dimers **4a** (amide dimer) and **4b** (thioamide dimer) derived from (*R*,*R*)- and (*S*,*S*)-ACPC residues (Figure 4a). Upon global deprotection, **4a** was soluble in DMF but became insoluble and precipitated under PyBOP/base coupling conditions, precluding efficient cyclization. In contrast, **4b** remained soluble under identical conditions and underwent clean head-to-tail macrocyclization, furnishing cyclic 4-, 6-, and 8-mers (**5a**–**5c**) in good yield. This solubility enhancement highlights a practical advantage of thioamide incorporation: reduced polarity and increased compatibility with organic solvents during macrocycle formation.

**Figure 4:**
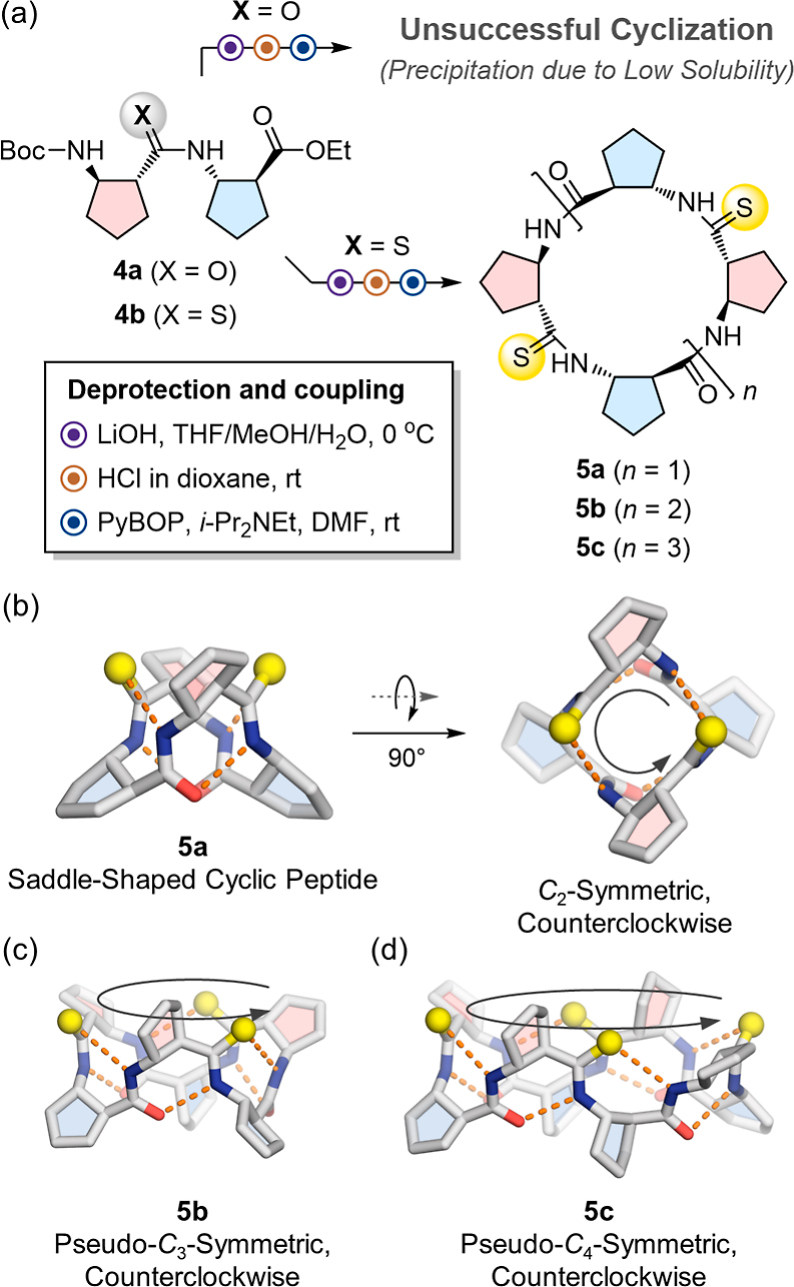
(a) Macrocyclization of heterochiral β-peptide dimers **4a** and **4b** via PyBOP-mediated head-to-tail coupling after global deprotection. Amide dimer **4a** fails to cyclize due to precipitation from low solubility, whereas thioamide dimer **4b** remains soluble, enabling efficient synthesis of cyclic 4-, 6-, and 8-mers (**5a**–**5c**). (b) Single-crystal structure of **5a**, a *C*_2_-symmetric, saddle-shaped cyclic peptide, oriented counterclockwise. (c) Single-crystal structure of **5b**, pseudo-*C*_3_-symmetric, oriented counterclockwise. (d) Single-crystal structure of **5c**, pseudo-*C*_4_-symmetric, oriented counterclockwise. Sulfur atoms are shown in yellow, and hydrogen bonds are depicted as orange dotted lines. Solvent molecules are omitted for clarity. The structure of **5b** and **5c** represents one conformer from the asymmetric unit.

Single-crystal XRD analysis revealed that **5a** adopts a *C*_2_-symmetric, saddle-shaped conformation stabilized by two C=O­(*i*)···H–N­(*i*+2) intramolecular hydrogen bonds (Figures 4b, S7, Table S5). Larger macrocycles **5b** and **5c** exhibit pseudo-*C*_3_ and pseudo-*C*_4_ symmetries, respectively, each forming an elliptical topology (Figure 4c,d). In all three macrocycles, amide and thioamide groups alternate in a counterclockwise orientation, with sulfur atoms projecting from one macrocycle face. This defined arrangement creates symmetry-related positions that can be selectively functionalized either through the distinct chemical reactivity of thioamides or via side chains oriented by the uniform ACPC chirality.

Solution NMR spectra of **5a**–**5c** are consistent with their crystallographic symmetries, displaying signal patterns characteristic of *C*_2_-, *C*_3_-, and *C*_4_-symmetric architectures (Figure S8). Interestingly, the larger macrocycles show evidence of conformational flexibility in solution, which may allow adaptive binding to different guest molecules. The combination of symmetry control and enhanced synthetic accessibility provided by thioamide substitution offers new opportunities for designing β-peptide macrocycles as chiral hosts, channel-forming scaffolds, or rigid frameworks for catalytic site organization.

We next applied thioamide substitution to address a persistent synthetic challenge in β-peptide chemistry: the preparation of long, well-folded oligomers in solution phase. Conventional solution-phase synthesis of β-peptides is typically restricted to relatively short sequences due to stepwise coupling inefficiencies, limited solubility of intermediates, and the cumulative difficulty of purifying highly hydrophobic oligomers.^52–54^ As a result, access to β-peptides beyond 12–16 residues generally requires specialized strategies or results in low overall yields.

Given the enhanced solubility imparted by thioamide incorporation, we hypothesized that long β-peptides could be prepared efficiently by solution-phase fragment condensation, bypassing the limitations of conventional approaches. To test this, we prepared thioamide-containing β-peptide fragments with *C*-terminal carboxylic acids of varying lengths (2-, 4-, and 8-mers) and performed stepwise fragment couplings (Figure 5a). This modular approach proved highly robust, affording 12-, 16-, 20-, and 24-mers (**8**–**11**) in ∼80% yield on a gram scale. Notably, we isolated a 32-mer (**12**) in over 200 mg quantity, with a molecular weight exceeding 4 kDa—approaching the size of a small protein and, to our knowledge, representing the longest β-peptide reported to date. These oligomers also showed remarkable stability; for example, the 12-mer **8** exhibited no detectable decomposition by ^1^H NMR even after 3 years at – 20 °C under nitrogen.

**Figure 5:**
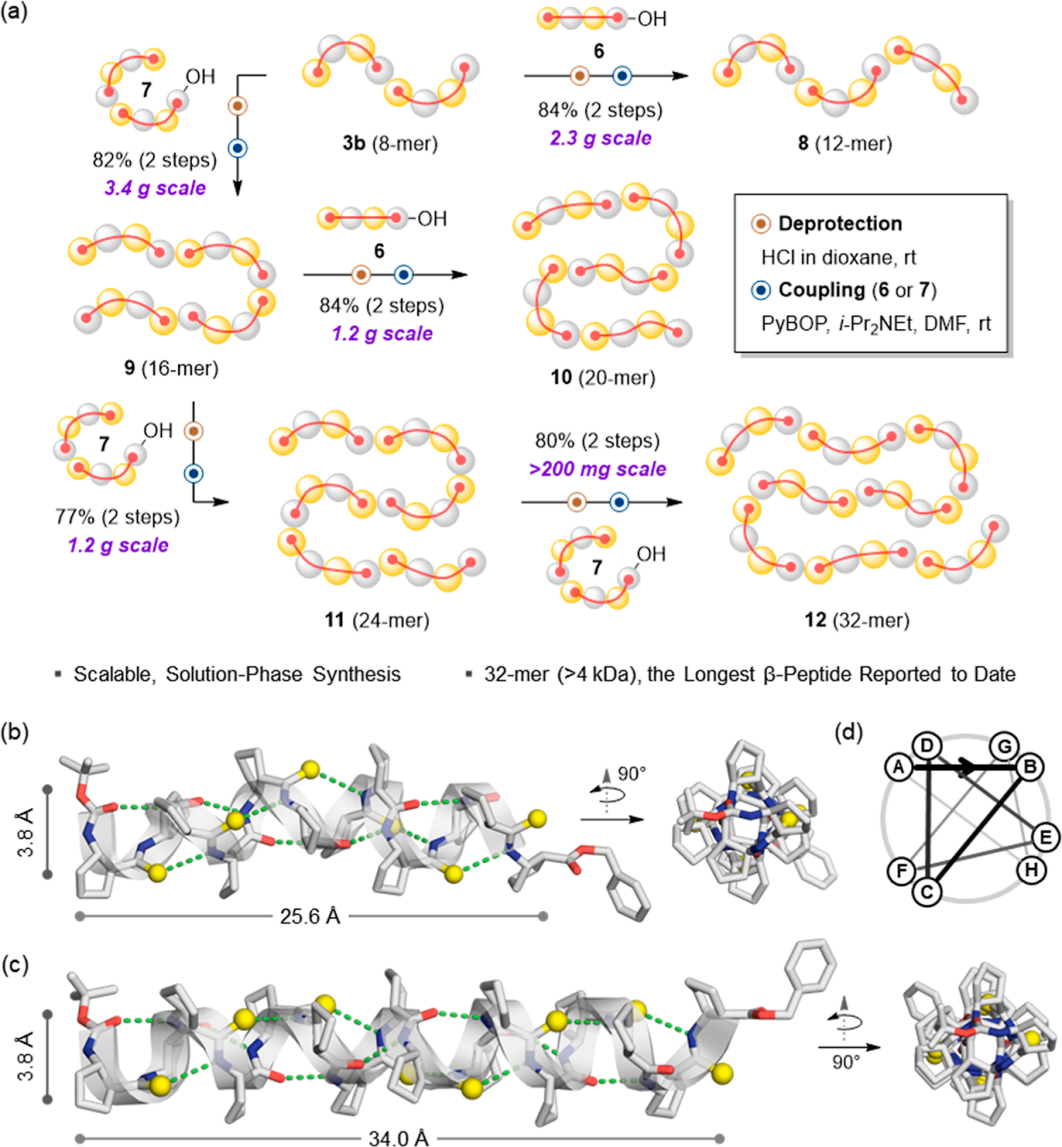
(a) Gram-scale solution-phase fragment condensation for the synthesis of long thioamide β-peptides. Fragment coupling of *C*-terminal acid and *N*-terminal amine fragments affords 8-mer (**3b**), 12-mer (**8**), 16-mer (**9**), 20-mer (**10**), 24-mer (**11**), and 32-mer (**12**) products in high yields. The 32-mer (>4 kDa) represents the longest β-peptide reported to date. (b) Single-crystal structure of 12-mer (**8**), adopting a 12-helix with a length of 25.6 Å. (c) Single-crystal structure of 16-mer (**9**), adopting a 12-helix with a length of 34.0 Å and a diameter of 3.8 Å. (d) Helical wheel diagram showing the octad repeat motif in 12- and 16-mers. Sulfur atoms are shown in yellow, and hydrogen bonds are depicted as green dotted lines. Solvent molecules and disordered regions are omitted for clarity.

Single-crystal XRD analysis of the 12-mer (**8**) and 16-mer (**9**) revealed that both maintain the canonical 12-helical conformation observed in shorter oligomers, extending to axial lengths of 25.6 Å and 34.0 Å, respectively (Figures 5b,c, S9, Table S6). The helices exhibit an octad repeat pattern, with each *trans*-ACPC residue contributing approximately 135° per turn, corresponding to 2.7 residues per turn, a diameter of 3.8 Å, and a pitch of 5.7 Å (Figures 5d, S10). This repeat-based architecture suggests that the helix is modular, with longer oligomers essentially behaving as concatenations of 8-residue units.

Circular dichroism (CD) spectroscopy in CHCl_3_ confirmed that the longer oligomers preserve 12-helical folding in solution (Figure S11). While minor spectral deviations were noted for the 8-mer (**3b**), the spectra of 12-, 16-, 20-, and 24-mers were nearly superimposable, indicating that the folding propensity is length-independent over this range. Although limited solubility precluded detailed solution-phase characterization of the 32-mer (**12**), its crystallographic and spectroscopic analogs suggest that it adopts a 12-helix approximately 7 nm in length, extrapolated from the measured length of the 16-mer.

The ability to prepare long, structurally defined β-peptides in gram quantities under solution-phase conditions has significant implications for foldamer-based materials. Such constructs could serve as rigid nanorods, programmable spacers for multivalent display, or building blocks for higher-order assemblies requiring precise control over axial length and surface chemistry. Thioamide-enabled fragment coupling thus represents a general, scalable route to β-peptides with record lengths and preserved secondary structure, opening avenues for applications where both molecular precision and preparative scale are essential.

With a robust solution-phase platform for synthesizing long thioamide β-peptides in hand, we next sought to exploit their chemical reactivity for postsynthetic backbone editing. Thioamides are known to undergo oxidative desulfurization under mild conditions, enabling conversion to the corresponding amides without altering the rest of the molecular framework. We envisioned that such a transformation could be applied to our long thioamide β-peptides to produce all-amide analogs that would otherwise be inaccessible by direct solution-phase synthesis.

After evaluating various oxidants, solvents, and additives, we identified a mild and scalable protocol using silver nitrate and sodium carbonate in aqueous dichloromethane (Figure 6a). This Ag­(I)-mediated oxidative desulfurization proceeded to completion within 30 min across a range of β-peptide lengths, affording the corresponding all-amide products in 97–99% isolated yields. Importantly, the reaction tolerated the Boc- and Bn-protecting groups present on the peptides, and the Ag_2_S byproduct could be removed simply by filtration, eliminating the need for chromatographic purification.

**Figure 6:**
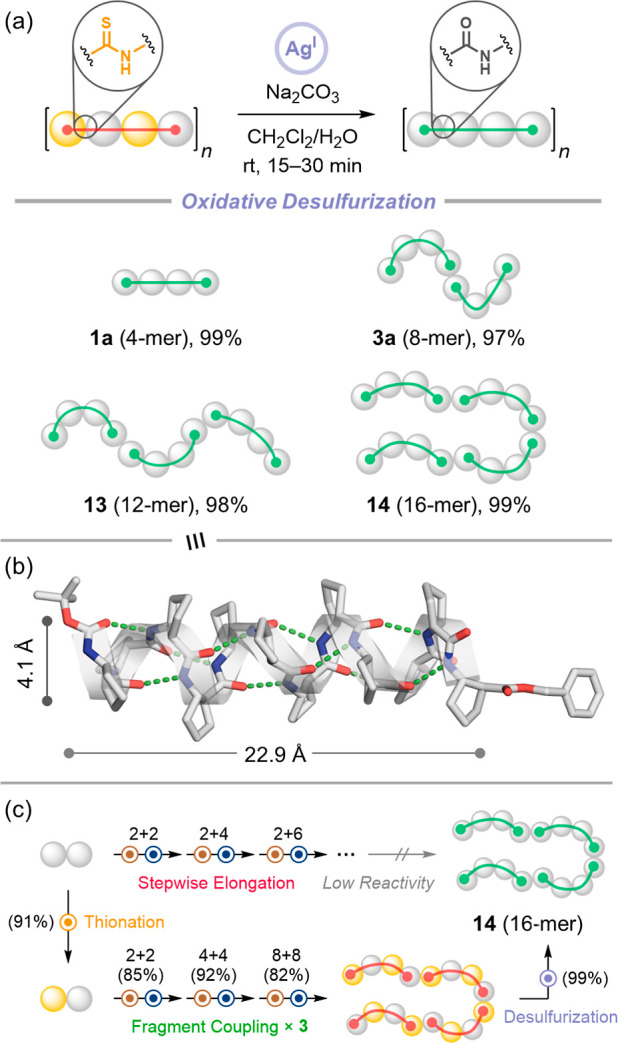
(a) Ag­(I)-mediated global oxidative desulfurization of long thioamide β-peptides. Treatment with silver nitrate and sodium carbonate in aqueous CH_2_Cl_2_ converts thioamide-containing oligomers into their all-amide counterparts (**13** and **14**) in 97–99% yield within 30 min. This mild, scalable protocol enables access to long β-peptides via solution-phase synthesis without chromatographic purification. Boc- and Bn-protecting groups are retained. (b) Single-crystal structure of 12-mer (**13**), adopting a 12-helix with a length of 22.9 Å and a diameter of 4.1 Å. Disordered regions are omitted for clarity. (c) Concise synthetic route to β-peptides enabled by thioamide substitution.

This strategy granted access to long all-amide β-peptides—including the 12-mer (**13**) and 16-mer (**14**)—that could not be obtained directly under our solution-phase synthesis conditions.^52,53,55,56^ XRD analysis of **13** revealed that it adopts the canonical 12-helical conformation, but with subtle geometric differences relative to its thioamide precursor **8**. Specifically, the replacement of longer C=S­(*i*)···H–N­(*i*+3) hydrogen bonds with shorter C=O­(*i*)···H–N­(*i*+3) bonds resulted in a decrease in axial length (22.9 Å vs 25.6 Å for **8**) and a slight increase in helix diameter (Figures 6b, S12, Table S7). These observations confirm that thioamide incorporation can be used to modulate hydrogen-bond geometry, and that the final scaffold properties can be fine-tuned postsynthetically through backbone conversion.

By decoupling conformational programming (via thioamides) from final backbone composition (via desulfurization), this platform enables modular generation of β-peptide libraries with tunable structures. Such orthogonal control over folding and composition opens new opportunities for systematic structure–function studies, optimization of foldamer–protein interactions, and the design of functional β-peptide materials. In combination with the scalable synthesis of long β-peptides, this postsynthetic editing strategy significantly broadens the accessible chemical space for foldamer-based applications (Figure 6c).

## Conclusion

In summary, we have established thioamides as versatile design elements for programmable control of β-peptide secondary structure. Site-selective incorporation of thioamide units modulates hydrogen-bond geometry to access noncanonical conformations—including curved 12-helices, conical 16/12-helices, and symmetry-defined macrocycles—while enhancing solubility to enable the scalable, solution-phase synthesis of long oligomers up to 32-mers. A mild Ag­(I)-mediated oxidative desulfurization further provides a postsynthetic backbone conversion to native amides, decoupling conformational control from final scaffold composition.

This combination of positional control, scalable synthesis, and orthogonal backbone editing offers a general strategy for accessing β-peptide architectures that were previously difficult or impossible to obtain. The ability to program helicity and macrocycle symmetry through minimal backbone modification expands the structural repertoire available for foldamer design, while the solubility benefits of thioamides open synthetic routes to record-length oligomers under solution-phase conditions. Moreover, the decoupling of conformational programming from final scaffold composition enables modular generation of β-peptide libraries for systematic structure–function studies.

Beyond their intrinsic structural appeal, these capabilities have broad potential in the creation of functional foldamer-based materials, molecular recognition systems, and peptide-inspired therapeutics. The programmable curvature and symmetry achieved here could be harnessed for the design of chiral channels, protein–peptide interface modulators, or self-assembling nanostructures with defined topology. We anticipate that the principles demonstrated with ACPC-based β-peptides will be transferable to other β-residue frameworks and mixed α/β architectures, providing a foundation for the next generation of foldamer chemistry and its translation into functional applications.

## Supplementary Material



## Notes

Detailed experimental procedures, compound characterization (^1^H, ^13^C NMR and HRMS), copies of NMR spectra of new compounds, and crystallographic data (PDF)

## ⊥

Jintaek Gong: Department of Chemistry Education, Sunchon National University, Sunchon 57922, Republic of Korea
