# Supplementary material for: Programmable Helicity and Macrocycle Symmetry in β‑Peptides via Site-Selective Thioamide Substitution
Source: J Am Chem Soc. 2025 Oct 29;147(48):44216–24. doi: 10.1021/jacs.5c13858 (PMC12679621; doi:10.1021/jacs.5c13858)
Supplement: Supplementary file 1 [file ja5c13858_si_001.pdf]

Supporting Information

Programmable Helicity and Macrocycle Symmetry in β-Peptides via Site-Selective Thioamide Substitution

Jungwoo Hong,[a,b] Jaewook Kim,[a,b] Jintaek Gong,[a] Seoneun Jeong,[a] Yi Sak Park,[a] Sung Hyun Yoo,[c] Jin Kim,[d] and Hee-Seung Lee*[,a,b]

[a]Department of Chemistry, Korea Advanced Institute of Science and Technology (KAIST), Daejeon 34141, Republic of Korea

[b]InnoCORE AI Co-Research & Education for Innovative Drug (AI-CRED) Institute, KAIST, Daejeon 34141, Republic of Korea

[c]Department of Chemistry, Chonnam National University, Gwangju 61186, Republic of Korea

[d]Department of Chemistry, Sunchon National University, Sunchon 57922, Republic of Korea

*Corresponding author. E-mail: hee-seung_lee@kaist.ac.kr

Table of Contents

1. Materials and Methods ········ **S3**

1.1. General Information ········ S3

1.2. Characterization ········ S3

1.3. Single Crystal X-ray Diffraction ········ S4

2. Synthetic Procedures and Characterization Data ········ **S5**

3. Supplementary Figures ········ **S33**

4. Supplementary Tables ········ **S41**

5. Crystallography ········ **S47**

5.1. Crystallization Methods ········ S47

5.2. Crystallographic Information ········ S48

6. Copies of NMR Spectra ········ **S50**

6.1. 1D NMR Spectra ········ S50

6.2. 2D NMR Spectra ········ S74

7. References ········ **S77**

1. Materials and Methods

1.1. General Information

All reactions were performed in oven-dried round-bottom flasks fitted with rubber septa under N_2 atmosphere. Commercially available reagents and solvents were purchased from Sigma-Aldrich, Alfa Aesar, TCI, and Combi-Blocks. Unless otherwise noted, reagents and solvents were used without additional purification. Tetrahydrofuran (THF) was freshly distilled under Na and benzophenone before use. CH_2Cl_2 was freshly distilled under P_2O_5 before use. *N*,*N*-Dimethylformamide (DMF) was filtered through alumina and stored over 4 Å molecular sieve. $CHCl_3$ was stored over 4 Å molecular sieve. *i*-Pr_2NEt and *N*-methylmorpholine (NMM) were distilled under CaH_2 and stored over KOH pellet. 4-Dimethylaminopyridine (DMAP) was recrystallized from EtOH. High-purity deionized water was afforded by Milli-Q apparatus. Analytical thin–layer chromatography (TLC) was performed using Merck (Darmstadt, Germany) silica gel 60 F_{254} glass plates. TLC plates were visualized using a 254 nm UV lamp, an acidic ninhydrin (Nin), an ethanolic phosphomolybdic acid (PMA), or a basic aqueous potassium permanganate ($KMnO_4$) solution. Purification of crude reaction mixtures by column chromatography was performed using Merck silica gel 60 (230–400 mesh).

1.2. Characterization

^1H and ^{13}C nuclear magnetic resonance (NMR) spectra were obtained with Bruker AVANCE NEO Nanobay (400 MHz for ^1H NMR, 101 MHz for ^{13}C NMR), Bruker AVANCE NEO (500 MHz for ^1H NMR, 126 MHz for ^{13}C NMR) and measured in $CDCl_3$, DMSO-d_6, pyridine-d_5, or $CD_3OH/CDCl_3$ (1/4, v/v) at the NMR facility of Department of Chemistry, KAIST. Chemical shifts were recorded as parts per million (ppm) relative to the residual solvent signal of $CDCl_3$ (δ_H = 7.26 ppm, δ_C = 77.16 ppm), DMSO-d_6 (δ_H = 2.50 ppm, δ_C = 39.52 ppm), pyridine-d_5 (δ_H = 7.58 ppm, δ_C = 135.91 ppm), or tetramethylsilane (TMS) (δ_H = 0.00 ppm, δ_C = 0.00 ppm) [multiplicity (s = singlet, d = doublet, t = triplet, q = quartet, app. p = apparent pentet, app. h = apparent heptet, m = multiplet, br = broad)] and coupling constants were reported in Hertz (Hz). All two-dimensional (2D) NMR experiments were conducted by following standard 2D correlation sequences at the NMR facility of the Department of Chemistry, KAIST. High-resolution mass spectrometry (HRMS) spectra were recorded on Waters Xevo G2-XS QTof spectrometer from KARA (KAIST Analysis Center for Research Advancement) (Daejeon, Republic of Korea) by using the ESI-positive method. Matrix-assisted laser desorption/ionization time-of-flight (MALDI-TOF) spectra were recorded on Bruker autoflex maX spectrometer from KARA by using positive ion mode. Circular dichroism (CD) spectra were recorded on JASCO J-1500 spectrophotometer with 1.0 mm path length quartz cells in 0.2 mM $CHCl_3$ solution and normalized by the number of amino acid residues. CD spectra were recorded from 420 to 240 nm with a scanning speed of 50 nm/min and were averaged 5 times.

1.3. Single Crystal X-ray Diffraction

Diffraction data of Boc–(*S*,*S*)-ACPC$_6$–OBn **2a** and Boc–(*S*,*S*)-ACPC$_8$–OBn **3a** single crystals were collected on a Rigaku R-AXIS RAPID II instrument on the Center for Research Facility of Sunchon National University (Sunchon, Republic of Korea) with graphite monochromated Mo Kα radiation (λ = 0.71073 Å) under a stream of nitrogen at 123 K. Cell parameter determination, refinement, data reduction, and absorption correction processes were performed using RAPID AUTO software. The detailed data processing options were not controlled arbitrarily, unlike in general settings. In other cases, the diffraction pattern was obtained at the Pohang Accelerator Laboratory synchrotron (Pohang, Republic of Korea) 2D-SMC beamline and 11C-Micro-MX beamline with monochromatic synchrotron radiation. The collected images were indexed, integrated, and scaled using *HKL-2000*[1]or *HKL-3000sm*[1,2]. Space group determination and data reduction were performed by the *XPREP* program from the *SHELXTL*[3] package. The structure was solved by direct methods with the *SHELXT*[4] program and refined with the *SHELXL*[5] embedded in the *OLEX2*[6,7] program. For some crystal samples, restraints for all bond lengths and angles were established using the Mogul Geometry Check based on the Cambridge Structural Database (CSD) for rational structural refinement. Each bond length and angle was restrained using the mean value, standard deviation, and number of hits in the CSD. All crystal data were registered at the Cambridge Crystallographic Data Centre (CCDC).

2. Synthetic Procedures and Characterization Data

General procedure A: hydrolysis of ester

Option A: To a solution of ester (1.0 equiv.) in THF/MeOH/H_2O (1/1/1, 0.1 to 0.2 M) was added lithium hydroxide monohydrate (5.0 equiv.) at 0 °C. The resulting suspension was stirred vigorously at the same temperature. After the full consumption of the starting material (checked by TLC, 4–5 h), the mixture was concentrated under reduced pressure to remove volatiles. The mixture was cooled to 0 °C, acidified with 1.0 M aqueous HCl (until pH becomes 2–3), and extracted with CH_2Cl_2 (three times). The combined organic layer was dried with anhydrous sodium sulfate. The mixture was concentrated under reduced pressure to afford the corresponding carboxylic acid, which was used for the next step without further purification.

Option B: To a solution of ester (1.0 equiv.) in THF/MeOH/H_2O (1/1/1, 0.05 M) was added lithium hydroxide monohydrate (10.0 equiv.) at 0 °C. The resulting suspension was stirred vigorously at room temperature until the full consumption of the starting material (checked by TLC, 2–3 h). The mixture was cooled to 0 °C, acidified with 1.0 M aqueous HCl (until pH becomes 2–3), and extracted with CH_2Cl_2 (three times). The combined organic layer was dried with anhydrous sodium sulfate. The mixture was concentrated under reduced pressure to afford the corresponding carboxylic acid, which was used for the next step without further purification.

General procedure B: amide coupling utilizing EDCI·HCl and HOBt

Amide coupling was conducted using 1-ethyl-3-(3-dimethylaminopropyl(carbodiimide) hydrochloride (EDCI·HCl) and 1-hydroxybenzotriazole monohydrate (HOBt·H_2O) as a coupling reagent. To a Boc-protected amine (1.0 equiv.), HCl (4.0 N in 1,4-dioxane, over 10 equiv., at least 0.8 mL) was added, and the resulting mixture was stirred under N_2 atmosphere. After the full consumption of the starting material (checked by TLC, generally 1 to 2 h), all volatiles were removed under reduced pressure and further dried under a high vacuum to afford the HCl salt of free amine. The salt was redissolved in a minimal amount of DMF and neutralized with *i*-Pr_2NEt (1.0 equiv.). In the other round-bottom flask, a carboxylic acid (1.1 equiv.) was dissolved in DMF (0.1 M), EDCI·HCl (1.3 equiv.), HOBt·H_2O (1.3 equiv.), and *i*-Pr_2NEt (3.0 equiv.) were added at room temperature. After 20 min of stirring under nitrogen atmosphere, the neutralized amine was cannulated to the activated carboxylic acid, and the RBF was washed twice with a minimal amount of additional DMF and subsequently cannulated. The resulting mixture was stirred at room temperature overnight and diluted with EtOAc (five-fold volume of overall DMF used). The organic layer was washed with 0.5 M aqueous HCl (same volume as EtOAc), saturated aqueous $NaHCO_3$ (half volume as EtOAc), brine (half amount as EtOAc), and dried over anhydrous magnesium sulfate. The crude mixture was purified by flash column chromatography to afford the pure product.

General procedure C: chemoselective thionation of amide utilizing Lawesson's reagent

To a solution of peptide (1.0 equiv.) in THF (0.1 M), Lawesson's reagent (0.6 equiv. for each amide) was added at room temperature under nitrogen atmosphere. The resulting suspension was refluxed until the full consumption of amide (generally 0.5–2 h) and concentrated under reduced pressure to remove all volatiles. The crude mixture was purified by flash column chromatography to afford thioamide.

Note: Repeated (2–3 times) chromatography is recommended to remove all byproducts from Lawesson's reagent.

General procedure D: amide coupling utilizing PyBOP

Option A: Amide coupling involving carboxylic acid containing thioamide group was conducted using benzotriazol-1-yloxytripyrrolidinophosphonium hexafluorophosphate (PyBOP) as a coupling reagent. To a Boc-protected amine (1.0 equiv.), HCl (4.0 N in 1,4-dioxane, 10 equiv.) was added, and the resulting mixture was stirred under N_2 atmosphere. After the full consumption of the starting material (checked by TLC, generally 1 to 2 h), all volatiles were removed under reduced pressure and further dried under a high vacuum to afford the HCl salt of free amine. The salt was redissolved in a minimal amount of DMF and neutralized with *i*-Pr_2NEt (1.0 equiv.). In the other round-bottom flask, a carboxylic acid (1.1 equiv.) was dissolved in DMF (0.1 M), and PyBOP (1.1 equiv.) and *i*-Pr_2NEt (1.5 equiv.) were added at room temperature. After 20 min of stirring under nitrogen atmosphere, the neutralized amine was cannulated to the activated carboxylic acid, and the RBF was washed twice with a minimal amount of additional DMF and subsequently cannulated. The resulting mixture was stirred at room temperature overnight and diluted with EtOAc (five-fold volume of overall DMF used). The organic layer was washed with 0.5 M aqueous HCl (same volume as EtOAc), saturated aqueous $NaHCO_3$ (half volume as EtOAc), brine (half amount as EtOAc), and dried over anhydrous magnesium sulfate. The crude mixture was purified by flash column chromatography to afford the desired product.

Option B: same as **option A**, but 1.25 equiv. of carboxylic acid and PyBOP was used.

Option C: After Boc group deprotection, the salt was dissolved in a minimal amount of $CHCl_3$, and neutralized with *i*-Pr_2NEt (1.0 equiv.). In the other round-bottom flask, a carboxylic acid (1.4 equiv.) was dissolved in $CHCl_3$ (0.1 M), and PyBOP (1.4 equiv.), and *i*-Pr_2NEt (2.0 equiv.) were added at room temperature. After 20 min of stirring under nitrogen atmosphere, the neutralized amine was cannulated to the activated carboxylic acid, and the RBF was washed twice with a minimal amount of additional $CHCl_3$ and subsequently cannulated. The resulting mixture was stirred at room temperature overnight, quenched with sat. aqueous NH_4Cl, and extracted with $CHCl_3$ three times. The combined organic layer was dried over magnesium sulfate. The crude mixture was purified by flash column chromatography to afford the desired product.

General procedure E: Ag(I)-mediated global oxidative desulfurization of thioamide

To a solution of thioamide peptide (1.0 equiv.) in CH_2Cl_2 (0.01–0.02 M) was added Na_2CO_3 (1.25 equiv. for each thioamide) and $AgNO_3$ (2.5 equiv. for each thioamide) in H_2O (0.1–0.2 M) at room temperature. After 10–30 min of vigorous stirring, the organic layer was filtered through a pad of celite and washed with $CHCl_3$ (5 × 3 mL). The mixture was concentrated under reduced pressure to afford pure, desulfurized peptide.

Scheme S1. Preparation of thioamide β-peptide fragments. Boc = *tert*-butoxycarbonyl, Bn = benzyl, DMF = *N,N*-dimethylformamide, THF = tetrahydrofuran, LR = Lawesson's reagent.

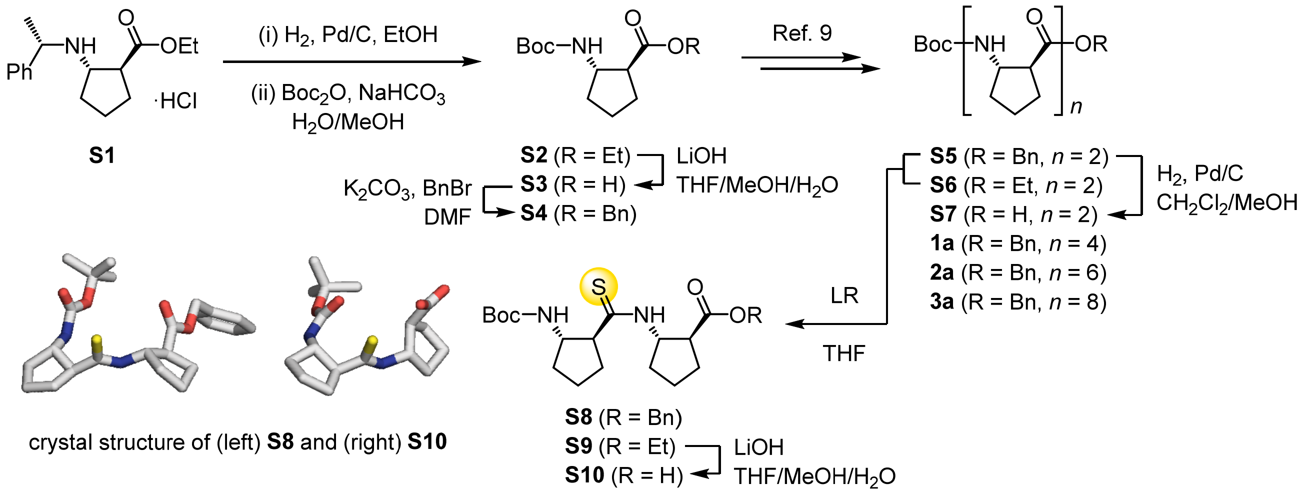

Note: (1S,2S)-2-aminocyclopentanecarboxylic acid (ACPC) oligomers ***1a*****–*****3a*** *were prepared by following the previous reports.*[8,9]

<u>Synthesis of **S2**</u>

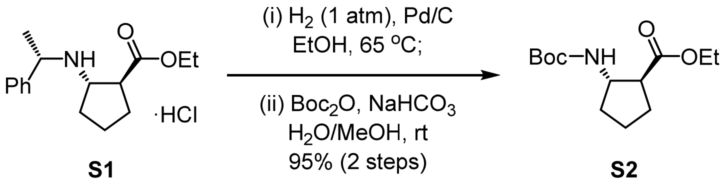

To a suspension of (*S*)-1-phenylethyl–(*S,S*)-ACPC–OEt hydrochloride **S1**[8] (17.5 g, 58.76 mmol) in EtOH (150 mL) was added 10% Pd/C (1.22 g) under under nitrogen gas and the inner atmosphere was exchanged to H_2 (using balloon) by repeating evacuation and back-filling five times. The resulting mixture was stirred vigorously at 65 °C for 6 h. After cooling down to room temperature, the reaction mixture was filtered through a pad of celite, washed with MeOH (3 × 50 mL), then concentrated under reduced pressure. The crude debenzylated product was diluted in H_2O/MeOH (1/1, 180 mL), then $NaHCO_3$ (14.81 g, 176.28 mmol) and di-*tert*-butyl decarbonate (Boc_2O, 14.85 mL, 64.64 mmol) were added slowly. The mixture was stirred at room temperature for 1.5 h and then concentrated under reduced pressure to remove all volatiles. The resulting mixture was diluted with saturated aqueous NH_4Cl (200 mL) and extracted with EtOAc (3 × 150 mL). The combined organic layer was washed with brine (150 mL), dried with anhydrous magnesium sulfate, and then concentrated under reduced pressure. The crude solid was recrystallized from hexane (180 mL) to afford **S2** as a colorless crystalline solid (14.32 g, 95%). Spectroscopic data were consistent with those previously reported.[9]

Synthesis of **S3**

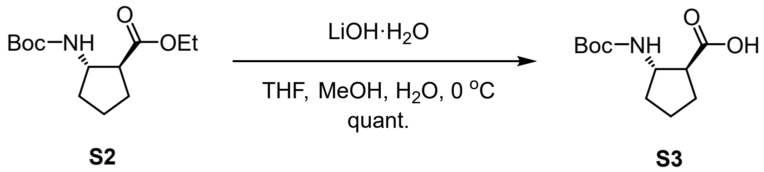

S3 was synthesized from **S2** (10.00 g, 38.86 mmol) by following the **general procedure A (option A)**. 8.89 g, quant., white solid. Spectroscopic data were consistent with those previously reported.[9]

Synthesis of **S4**

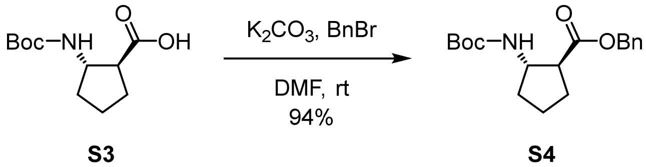

To a solution of **S3** (6.00 g, 26.17 mmol) in DMF (100 mL) was added dried K_2CO_3 (4.70 g, 34.02 mmol) and benzyl bromide (3.73 mL, 31.40 mmol) slowly under nitrogen atmosphere. The mixture was stirred vigorously at room temperature for 5 h. The resulting mixture was diluted with EtOAc (400 mL), washed with saturated aqueous NH_4Cl (400 mL), saturated aqueous $NaHCO_3$ (200 mL), and brine (200 mL). The organic layer was dried with anhydrous magnesium sulfate and concentrated under reduced pressure. The crude solid was recrystallized from hexane (180 mL) to afford **S4** as a colorless solid (7.88 g, 94 %). Spectroscopic data were consistent with those previously reported.[9]

Synthesis of **S5**

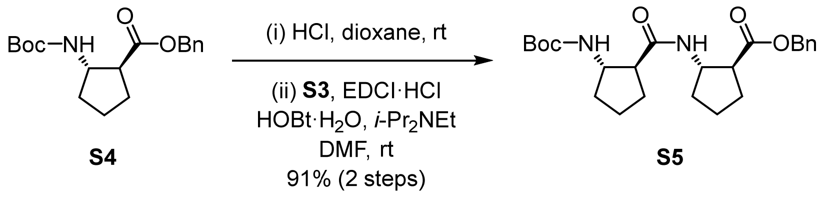

A solution of **S4** (5.00 g, 15.65 mmol by following the **general procedure B**. Purified by recrystallization from acetone/hexane (1/2, 300 mL), 6.27 g, 93%, white solid. Spectroscopic data were consistent with those previously reported.[9]

Synthesis of **S6**

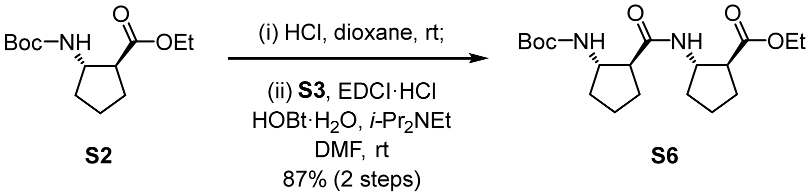

S2 **S6**

S6 was prepared from **S2** (3.00 g, 11.66 mmol) by following the **general procedure B**. Purified by recrystallization from acetone/hexane (1/2, 200 mL), 3.72 g, 87%, white solid. Spectroscopic data were consistent with those previously reported.[9]

Synthesis of **S7**

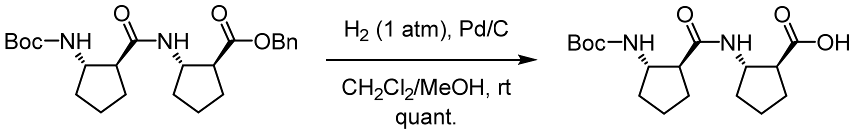

S5 **S7**

To a solution of **S5** (2.49 g, 5.78 mmol) in CH_2Cl_2/MeOH (1/3, 23.1 mL) was added 10% Pd/C (0.25 g) under nitrogen gas, and the inner atmosphere was exchanged to H_2 (using a balloon) by repeating evacuation and back-filling five times. After the vigorous stirring at room temperature for 1.5 h, the reaction mixture was filtered through a pad of celite, washed with CH_2Cl_2/MeOH (1/1, 3 × 10 mL), then concentrated under reduced pressure to afford pure **S7** (1.96 g, quant.) as a white solid. Spectroscopic data were consistent with those previously reported.[9]

Synthesis of **S8**

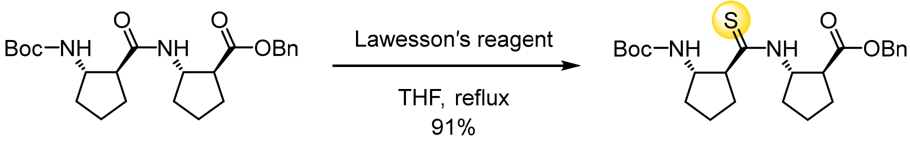

S5 **S8**

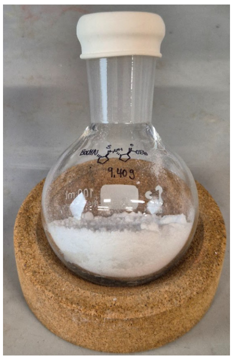

S8 was synthesized from **S5** (10.00 g, 23.226 mmol) by following the **general procedure C**. Purified by flash column chromatography (CH_2Cl_2/Et_2O/hexane = 2/3/5), 9.40 g, 91%, white solid.

^{1}H NMR (400 MHz, $CDCl_3$) δ 9.73 (s, 1H), 7.37–7.24 (m, 5H), 5.18–5.00 (m, 3H), 4.70 (d, J = 6.4 Hz, 1H), 3.77 (t, J = 5.8 Hz, 1H), 2.99–2.86 (m, 2H), 2.35–2.19 (m, 2H), 2.14–1.99 (m, 2H), 1.98–1.85 (m, 2H), 1.83–1.76 (m, 2H), 1.73–1.57 (m, 3H), 1.45–1.34 (m, 10H).

^{13}C NMR (101 MHz, $CDCl_3$) δ 204.72, 174.52, 156.68, 136.11, 128.51, 128.15, 128.08, 80.42, 66.62, 60.59, 60.13, 57.45, 50.05, 33.14, 31.97, 31.60, 28.86, 28.42, 23.71, 23.51.

HRMS (ESI) m/z: $[M+Na]^+$ calcd for 469.2131, found: 469.2114.

Synthesis of **S9**

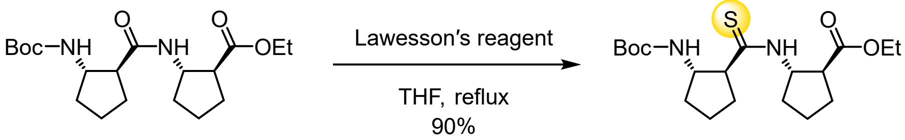

S6 **S9**

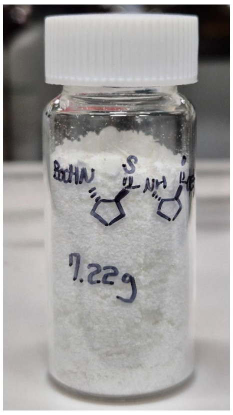

S9 was synthesized from **S6** (7.70 g, 20.897 mmol) by following the **general procedure for C**. Purified by flash column chromatography (CH_2Cl_2/Et_2O/hexane = 2/3/5), 7.22 g, 90 %, white solid.

^{1}H NMR (400 MHz, $CDCl_3$) δ 9.73 (s, 1H), 5.01 (p, J = 6.7 Hz, 1H), 4.72 (d, J = 6.7 Hz, 1H), 4.12 (qd, J = 7.1, 1.6 Hz, 2H), 3.88 (t, J = 6.2 Hz, 1H), 2.96 (q, J = 6.9, 6.2 Hz, 1H), 2.83 (dt, J = 8.9, 6.9 Hz, 1H), 2.35 (dq, J = 13.2, 8.2 Hz, 1H), 2.23 (dt, J = 12.9, 7.0 Hz, 1H), 2.11–1.86 (m, 4H), 1.83–1.73 (m, 3H), 1.72–1.60 (m, 2H), 1.50–1.40 (m, 10H), 1.23 (t, J = 7.1 Hz, 3H).

^{13}C NMR (101 MHz, $CDCl_3$) δ 204.65, 174.73, 156.78, 80.63, 60.82, 60.81, 60.20, 57.60, 50.29, 33.35, 32.05, 31.74, 28.96, 28.51, 23.80, 23.65, 14.34.

HRMS (ESI) m/z: $[M+Na]^+$ calcd for 407.1975, found: 407.1985.

Synthesis of **S10**

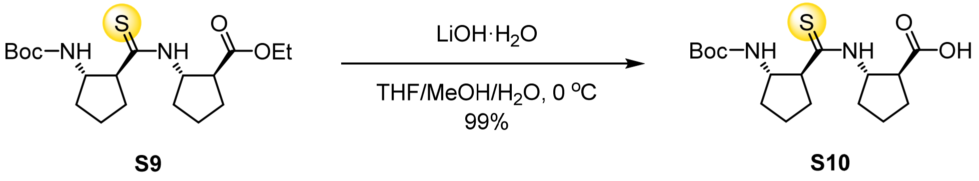

S10 was synthesized from **S9** (7.00 g, 18.204 mmol) by following the **general procedure A (option A)**. 6.45 g, 99%, white solid.

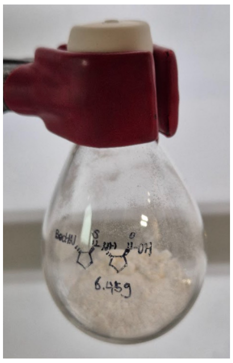

^{1}H NMR (500 MHz, DMSO-d_6) δ 12.13 (s, 1H), 9.70 (d, J = 6.9 Hz, 1H), 6.74 (d, J = 8.0 Hz, 1H), 4.79 (tt, J = 7.3, 4.5 Hz, 1H), 4.04 (app. p, J = 7.7 Hz, 1H), 2.87 (q, J = 8.3 Hz, 1H), 2.78 (dt, J = 9.6, 5.0 Hz, 1H), 2.05 – 1.79 (m, 5H), 1.74 – 1.39 (m, 7H), 1.35 (s, 9H).

^{13}C NMR (126 MHz, DMSO-d_6) δ 205.47, 175.69, 154.98, 77.53, 58.85, 57.98, 57.76, 48.96, 32.98, 32.11, 31.37, 29.38, 28.25, 23.86, 22.97.

HRMS (ESI) m/z: $[M+Na]^+$ calcd for 379.1662, found: 379.1668.

Scheme S2. Synthesis of thioamide β-peptides. EDCI = 1-ethyl-3-(3-dimethylaminopropyl)carbodiimide, HOBt = hydroxybenzotriazole, PyBOP = (benzotriazol-1-yloxy) tripyrrolidinophosphonium hexafluorophosphate, LR = Lawesson's reagent.

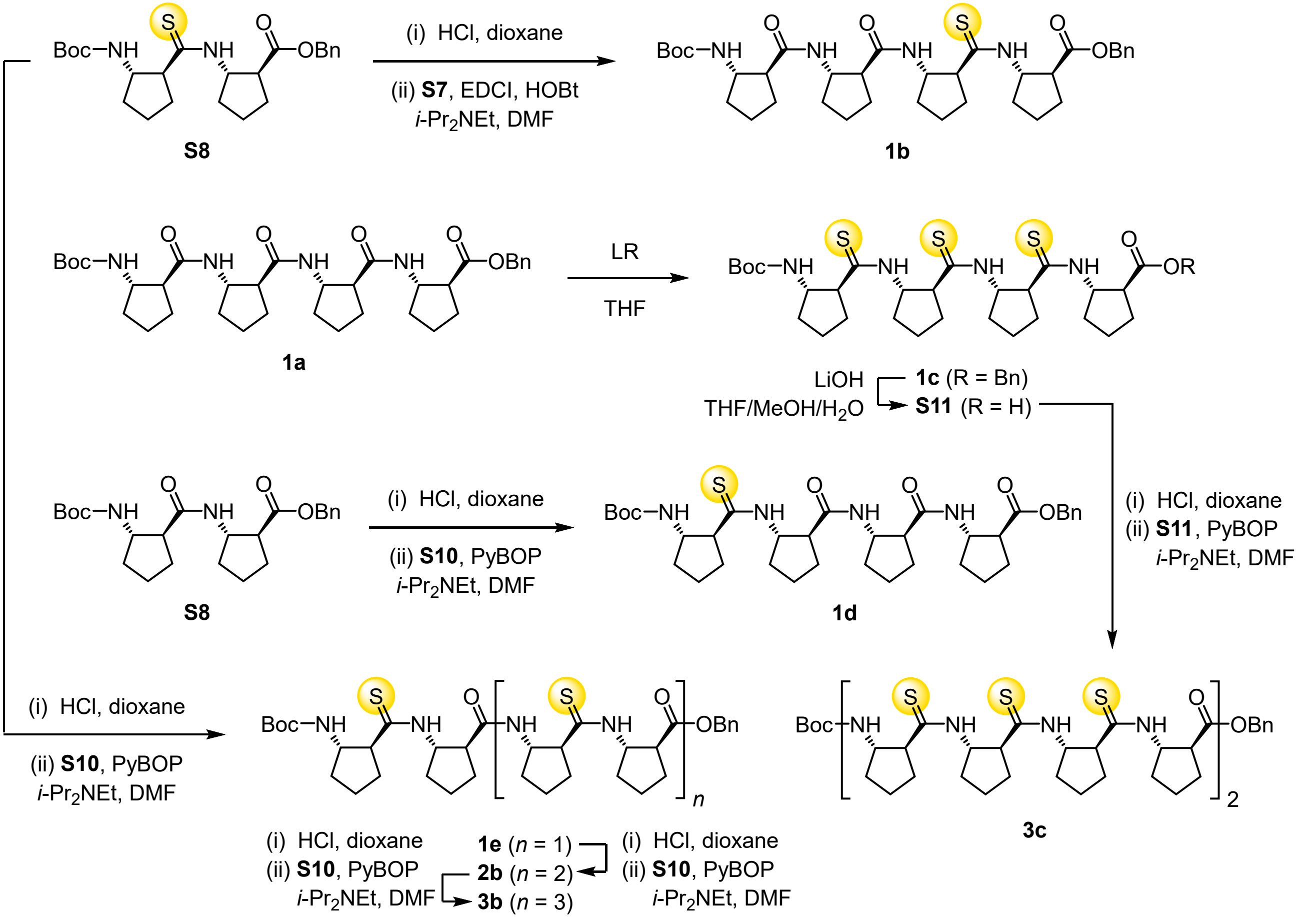

Synthesis of **1b**

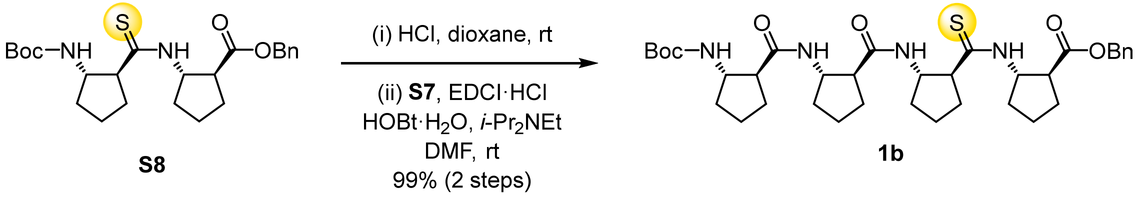

1b was prepared from **S8** (30 mg, 0.067 mmol) by following the **general procedure B**. Purified by flash column chromatography (EtOAc/hexane = 6/4), 44.6 mg, 99%, white solid.

^{1}H NMR (400 MHz, Pyridine-d_5) δ 10.81 (d, J = 7.0 Hz, 1H), 9.26 (d, J = 8.1 Hz, 1H), 8.28 (d, J = 8.4 Hz, 2H), 7.54 (d, J = 7.5 Hz, 2H), 7.35 (t, J = 7.5 Hz, 2H), 7.26 (t, J = 7.4 Hz, 1H), 5.71 (td, J = 7.5, 3.6 Hz, 1H), 5.35 (ABq, J = 12.6 Hz, 2H), 4.92 (td, J = 8.7, 4.5 Hz, 1H), 4.74 (app. p, J = 7.8 Hz, 1H), 4.49 (app. p, J = 7.8 Hz, 1H), 3.85 (dt, J = 9.2, 4.7 Hz, 1H), 3.53 (q, J = 7.0 Hz, 1H), 2.92 (dt, J = 13.7, 6.9 Hz, 1H), 2.77 (q, J = 8.1 Hz, 1H), 2.67 (q, J = 7.6 Hz, 1H), 2.32 (td, J = 11.4, 10.3, 4.8 Hz, 3H), 2.20 (dt, J = 13.5, 6.8 Hz, 1H), 2.13–1.89 (m, 9H), 1.89–1.62 (m, 9H), 1.60–1.53 (m, 1H), 1.53 (s, 9H).

^{13}C NMR (126 MHz, Pyridine-d_5) δ 208.23, 176.01, 175.86, 174.88, 157.32, 137.76, 129.17, 128.70, 128.50, 79.43, 66.67, 60.67, 60.40, 58.62, 57.89, 56.58, 54.82, 54.35, 50.42, 34.75, 33.87, 33.69, 33.52, 32.71, 31.02, 29.95, 29.84, 28.98, 26.47, 25.89, 25.39, 24.95.

HRMS (ESI) m/z calcd for [M+Na]$^+$: 691.3500, found: 691.3497.

Synthesis of **1c**

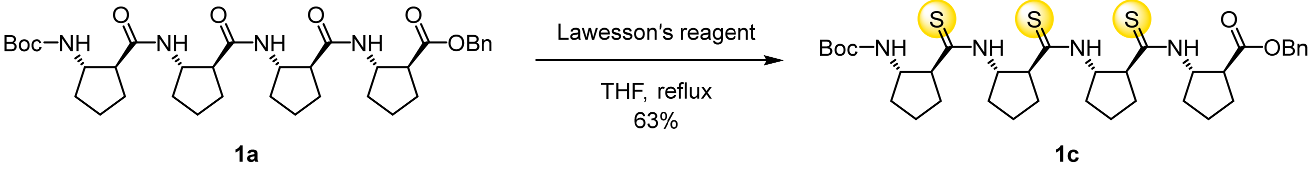

1c was synthesized from **1a** (100.0 mg, 0.153 mmol) by following the **general procedure C**. Purified by flash column chromatography (EtOAc/hexane = 3/7 to 4/6), 68.0 mg, 63%, white solid.

^{1}H NMR (400 MHz, $CDCl_3$) δ 9.64 (d, J = 7.1 Hz, 1H), 9.44 (d, J = 6.8 Hz, 1H), 9.14 (d, J = 6.1 Hz, 1H), 7.40–7.24 (m, 5H), 5.19–5.01 (m, 3H), 4.80 (d, J = 6.5 Hz, 1H), 4.70 (app. h, J = 3.9 Hz, 1H), 4.55 (tt, J = 7.0, 2.9 Hz, 1H), 3.83 (app. p, J = 6.7 Hz, 1H), 3.30 (dt, J = 7.8, 4.7 Hz, 1H), 3.11 (t, J = 8.7 Hz, 1H), 3.00 (dt, J = 8.8, 6.7 Hz, 1H), 2.83 (q, J = 7.9 Hz, 1H), 2.64–2.50 (m, 2H), 2.43 (dq, J = 15.1, 7.7 Hz, 1H), 2.27–2.06 (m, 4H), 2.05–1.78 (m, 12H), 1.75–1.60 (m, 4H), 1.44 (m, 10H).

^{13}C NMR (101 MHz, $CDCl_3$) δ 205.52, 205.26, 204.63, 174.73, 156.71, 136.24, 128.54, 128.20, 128.06, 80.90, 77.48, 77.16, 76.84, 66.64, 64.28, 64.18, 61.56, 60.27, 59.50, 59.09, 58.38, 50.20, 34.03, 32.36, 31.99, 31.86, 31.26, 30.78, 29.62, 29.30, 28.55, 24.65, 24.38, 24.15, 24.12.

HRMS (ESI) m/z calcd for [M+Na]$^+$: 723.3043, found: 723.3063.

Synthesis of **1d**

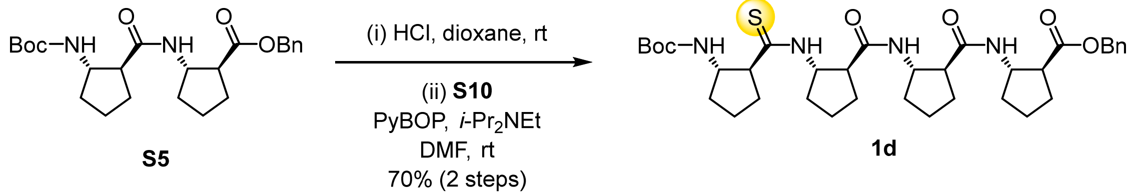

1d was synthesized from **S5** (50.0 mg, 0.116 mmol) by following the **general procedure for D (option A)**. Purified by flash column chromatography (EtOAc/hexane = 6/4 to 7/3), 54.2 mg, 70 %, white solid.

^{1}H NMR (500 MHz, $CDCl_3$) δ 9.34 (d, *J* = 7.6 Hz, 1H), 8.16 (d, *J* = 7.6 Hz, 1H), 7.68 (d, *J* = 7.0 Hz, 1H), 7.34–7.27 (m, 5H), 5.20–5.01 (m, 3H), 4.82 (app. p, J = 7.2 Hz, 1H), 4.44 (app. p, J = 7.3 Hz, 1H), 4.09 (dt, *J* = 12.5, 7.1 Hz, 1H), 3.88 (app. p, J = 6.7 Hz, 1H), 2.89 (q, *J* = 7.5 Hz, 1H), 2.82 (q, *J* = 7.8 Hz, 1H), 2.70–2.62 (m, 2H), 2.40 (dq, *J* = 15.3, 7.8 Hz, 1H), 2.15 (dt, *J* = 17.0, 7.7 Hz, 1H), 2.11–2.00 (m, 5H), 2.00–1.87 (m, 2H), 1.86–1.75 (m, 7H), 1.74–1.58 (m, 6H), 1.49 (dt, *J* = 13.0, 6.9 Hz, 2H), 1.43 (s, 9H).

^{13}C NMR (126 MHz, $CDCl_3$) δ 205.75, 175.16, 174.51, 173.83, 156.68, 136.38, 128.51, 128.03, 128.00, 80.43, 66.39, 61.63, 61.05, 59.15, 55.86, 54.82, 52.90, 52.16, 50.64, 33.81, 33.08, 32.93, 32.22, 31.84, 28.74, 28.53, 27.59, 27.17, 24.68, 24.66, 24.31, 23.39.

HRMS (ESI) *m/z* calcd for $[M+Na]^+$: 691.3500, found: 691.3497.

Synthesis of **1e**

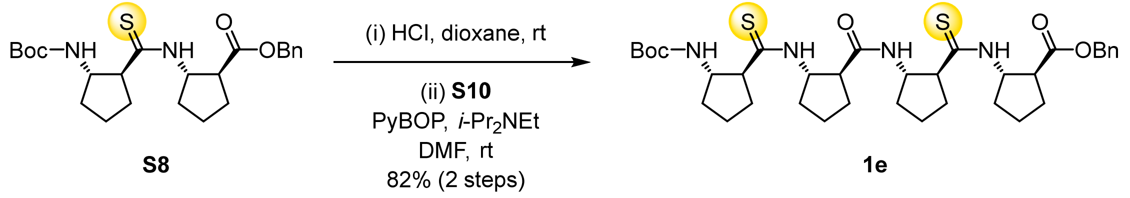

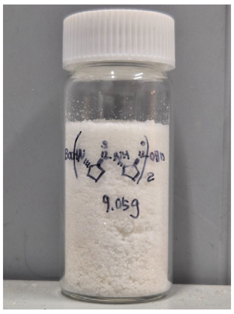

1e was synthesized from **S8** (7.20 g, 16.121 mmol) by following the **general procedure D (option A)**. Purified by flash column chromatography (EtOAc/Hexane = 5/5 to 6/4), 9.05 g, 82%, white solid.

^{1}H NMR (500 MHz, $CDCl_3$) δ 10.12 (d, *J* = 7.2 Hz, 1H), 9.22 (d, *J* = 7.4 Hz, 1H), 7.66 (d, *J* = 7.2 Hz, 1H), 7.37–7.24 (m, 6H), 5.17–5.02 (m, 3H), 4.86–4.72 (m, 2H), 3.99 (tt, *J* = 7.4, 5.0 Hz, 1H), 3.81 (app. p, *J* = 6.4 Hz, 1H), 3.02–2.93 (m, 2H), 2.85 (td, *J* = 7.9, 4.9 Hz, 1H), 2.70 (q, *J* = 6.6 Hz, 1H), 2.48–2.32 (m, 2H), 2.28–1.90 (m, 8H), 1.88–1.76 (m, 7H), 1.75–1.58 (m, 5H), 1.55–1.39 (m, 11H).

^{13}C NMR (126 MHz, $CDCl_3$) δ 205.52, 204.86, 174.80, 174.49, 156.82, 136.31, 128.54, 128.20, 128.06, 80.86, 66.63, 61.79, 61.34, 60.44, 60.32, 58.91, 57.25, 52.01, 50.03, 33.90, 32.87, 32.20, 32.15, 31.85, 31.78, 29.01, 28.55, 26.88, 24.45, 24.32, 24.26, 23.87.

HRMS (ESI) *m/z* calcd for $[M+Na]^+$: 707.3271, found: 707.3364.

Synthesis of **2b**

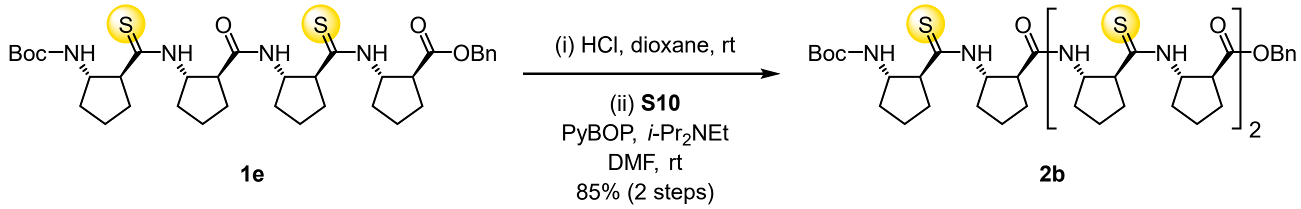

2b was synthesized from **1e** (44.6 mg, 0.065 mmol) by following the **general procedure D (option A)**. Purified by flash column chromatography (EtOAc/Hexane = 6/4), 51.1 mg, 85%, white solid.

^{1}H NMR (500 MHz, Pyridine-d_5) δ 11.31 (d, J = 8.8 Hz, 1H), 10.96 (d, J = 7.2 Hz, 1H), 9.96 (d, J = 9.0 Hz, 1H), 8.88 (d, J = 7.3 Hz, 1H), 8.57 (d, J = 8.3 Hz, 1H), 8.40 (d, J = 8.0 Hz, 1H), 7.51 (d, J = 7.3 Hz, 2H), 7.36 (dd, J = 8.3, 6.8 Hz, 2H), 7.28 (t, J = 7.3 Hz, 1H), 5.74–5.57 (m, 3H), 5.31 (ABq, J = 12.5 Hz, 2H), 4.75 (ddd, J = 13.9, 8.0, 6.0 Hz, 1H), 4.65–4.53 (m, 2H), 3.83 (ddd, J = 9.3, 6.4, 3.7 Hz, 1H), 3.59 (dt, J = 9.1, 6.7 Hz, 1H), 3.53 (dt, J = 9.1, 6.4 Hz, 1H), 3.32 (dt, J = 9.0, 6.4 Hz, 1H), 3.13 (q, J = 7.6 Hz, 1H), 3.03 (dq, J = 13.5, 6.8 Hz, 1H), 2.91 (dt, J = 9.2, 6.4 Hz, 1H), 2.79 (dq, J = 13.7, 7.0 Hz, 1H), 2.65 (dq, J = 13.9, 7.2 Hz, 1H), 2.42–2.29 (m, 2H), 2.27–2.09 (m, 7H), 2.08–1.84 (m, 16H), 1.84–1.61 (m, 8H), 1.49 (s, 9H).

^{13}C NMR (126 MHz, Pyridine-d_5) δ 208.12, 208.06, 207.31, 175.84, 175.49, 175.40, 157.42, 137.59, 129.22, 128.69, 128.58, 79.62, 66.85, 62.55, 61.34, 61.34, 61.09, 61.06, 61.01, 59.95, 59.57, 59.54, 53.15, 52.45, 50.68, 34.50, 34.20, 33.94, 33.76, 33.73, 33.68, 33.43, 32.92, 32.65, 30.39, 30.15, 29.88, 28.99, 26.49, 26.40, 26.25, 26.13, 26.09, 24.83.

HRMS (ESI) *m*/*z* calcd for $[M+Na]^+$: 945.4411, found: 945.4395.

Synthesis of **3b**

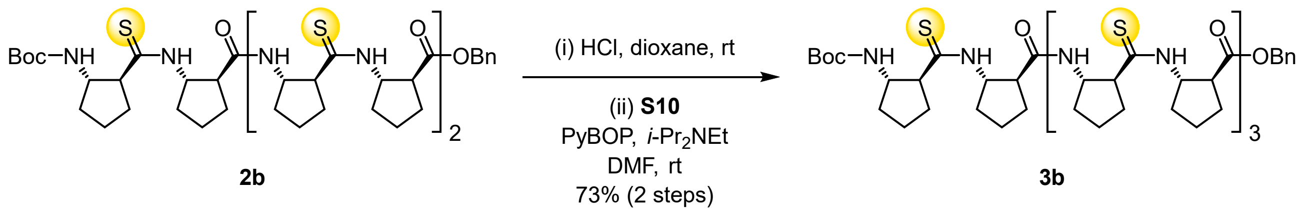

3b was synthesized from **2b** (25.8 mg, 0.028 mmol) by following the **general procedure D (option A)**. Purified by flash column chromatography (EtOAc/Hexane = 7/3), 23.6 mg, 73 %, white solid.

^{1}**H NMR** (500 MHz, $CDCl_3$) δ 10.12 (d, J = 7.1 Hz, 1H), 9.07 (d, J = 9.3 Hz, 1H), 9.02 (d, J = 9.7 Hz, 1H), 8.71 (d, J = 9.4 Hz, 1H), 8.51 (d, J = 8.6 Hz, 1H), 8.45 (d, J = 7.7 Hz, 1H), 7.67 (d, J = 8.5 Hz, 1H), 7.37–7.26 (m, 5H), 5.42 (d, J = 8.0 Hz, 1H), 5.20 – 5.03 (m, 6H), 4.36–4.25 (m, 2H), 4.14–4.01 (m, 2H), 3.22–3.14 (m, 2H), 3.01–2.91 (m, 2H), 2.88 (q, J = 7.4 Hz, 1H), 2.66–2.57 (m, 2H), 2.50–2.33 (m, 5H), 2.24–1.95 (m, 12H), 1.95–1.66 (m, 27H), 1.66–1.52 (m, 5H), 1.43 (s, 9H).

^{13}**C NMR** (126 MHz, $CDCl_3$) δ 207.36, 207.11, 206.89, 206.65, 175.52, 174.78, 174.23, 173.98, 156.50, 136.27, 128.55, 128.03, 127.96, 80.26, 66.52, 62.38, 61.01, 60.93, 60.91, 60.74, 60.66, 60.27, 60.08, 59.29, 58.27, 58.25, 57.94, 52.20, 51.28, 51.03, 49.92, 34.37, 33.79, 33.64, 33.50, 33.49, 33.39, 33.37, 33.27, 33.17, 32.78, 32.29, 32.22, 29.73, 29.23, 29.00, 28.85, 28.64, 25.77, 25.71, 25.59, 25.54, 25.44, 25.28, 25.08, 24.31.

HRMS (ESI) m/z calcd for $[M+Na]^+$: 1183.5551, found: 1183.5518.

*Note: **3b** can also be synthesized from **1e** on a multi-gram scale, as described later.*

Synthesis of **S11**

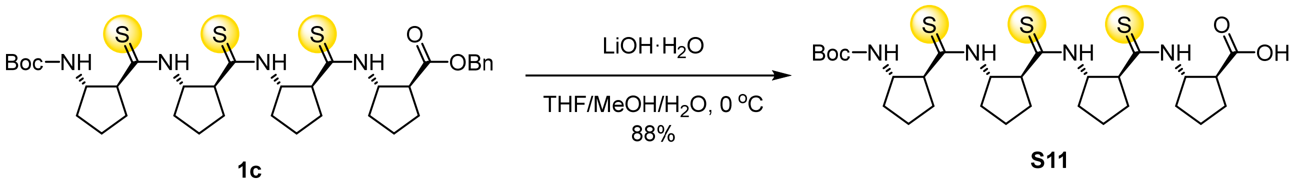

S11 was synthesized from **1c** (75 mg, 0.107 mmol) by following the **general procedure A (option B)**. Used for subsequent reaction without further purification, 57.5 mg, 88 %, white solid.

^{1}**H NMR** (500 MHz,) δ 9.51 (d, J = 6.5 Hz, 1H), 9.41 (d, J = 7.6 Hz, 1H), 8.77 (d, J = 8.3 Hz, 1H), 4.97–4.87 (m, 2H), 4.76 (dt, J = 8.5, 4.4 Hz, 1H), 4.69 (dq, J = 7.2, 3.5 Hz, 1H), 3.91 (app. p, J = 7.5 Hz, 1H), 3.35 (s, 1H), 3.24 (t, J = 6.8 Hz, 1H), 3.02–2.91 (m, 1H), 2.79 (q, J = 7.6 Hz, 1H), 2.55–2.37 (m, 3H), 2.24–2.07 (m, 4H), 2.06–1.79 (m, 13H), 1.78–1.65 (m, 3H), 1.49 (dt, J = 13.7, 7.3 Hz, 1H), 1.43 (s, 9H).

^{13}**C NMR** (126 MHz,) δ 206.21, 205.91, 205.78, 175.40, 156.53, 80.84, 63.89, 63.75, 62.01, 61.49, 60.05, 59.56, 58.26, 51.36, 34.21, 32.84, 32.41, 32.14, 32.07, 31.93, 31.13, 28.57, 27.55, 25.42, 25.35, 25.00, 24.49.

HRMS (ESI) m/z: $[M+Na]^+$ calcd for 633.2573, found: 633.2567.

Synthesis of **3c**

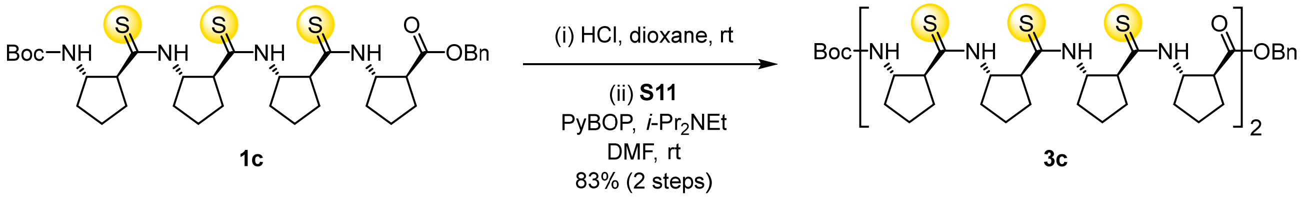

3c was synthesized from **1c** (80.0 mg, 0.114 mmol) by following the **general procedure D (option B)**. Purified by flash column chromatography (CH_2Cl_2/EtOAc/Hexane = 2/4/4), 113.1 mg, 83 % (from **1c**), white solid.

^{1}H NMR (500 MHz, Pyridine-d_5) δ 11.11 (d, J = 8.0 Hz, 1H), 10.57 (d, J = 7.4 Hz, 1H), 10.41 (d, J = 7.9 Hz, 1H), 10.27 (d, J = 7.2 Hz, 1H), 10.19 (d, J = 6.9 Hz, 1H), 10.02 (d, J = 8.8 Hz, 1H), 8.74 (d, J = 7.9 Hz, 1H), 8.40 (d, J = 8.0 Hz, 1H), 7.53 (d, J = 7.5 Hz, 2H), 7.35 (t, J = 7.5 Hz, 2H), 7.27 (t, J = 7.3 Hz, 1H), 5.73 (td, J = 8.5, 4.1 Hz, 1H), 5.63–5.48 (m, 3H), 5.45–5.38 (m, 2H), 5.33 (ABq, J = 12.5 Hz, 2H), 4.75 (app. p, J = 7.2 Hz, 1H), 4.54 (app. p, J = 7.5 Hz, 1H), 3.97 (dt, J = 8.1, 3.9 Hz, 1H), 3.90 (ddd, J = 9.0, 5.8, 3.3 Hz, 1H), 3.73–3.60 (m, 2H), 3.45 (dt, J = 8.9, 6.1 Hz, 1H), 3.41–3.30 (m, 2H), 3.27 (q, J = 7.4 Hz, 1H), 2.88 (dq, J = 12.9, 7.2 Hz, 1H), 2.80 (dq, J = 12.9, 6.4 Hz, 1H), 2.75 – 2.56 (m, 4H), 2.39–2.15 (m, 10H), 2.17–1.83 (m, 25H), 1.84–1.64 (m, 7H), 1.48 (s, 9H).

^{13}C NMR (126 MHz, Pyridine-d_5) δ 207.87, 207.85, 207.84, 207.55, 207.29, 207.13, 175.88, 175.46, 157.37, 137.56, 129.20, 128.71, 128.57, 79.65, 66.82, 64.70, 64.51, 63.96, 63.68, 62.14, 62.07, 61.51, 61.13, 60.98, 60.90, 60.06, 59.74, 59.25, 58.87, 52.15, 50.45, 34.34, 34.22, 34.21, 34.14, 34.06, 34.00, 33.77, 33.63, 33.57, 33.47, 33.43, 32.98, 32.86, 32.60, 30.72, 30.60, 29.00, 26.96, 26.85, 26.60, 26.49, 26.09, 26.03, 25.71, 25.17.

HRMS (ESI) m/z calcd for $[M+Na]^+$: 1215.5094, found: 1215.5104.

Scheme S3. Synthesis of cyclic thioamide β-peptides.

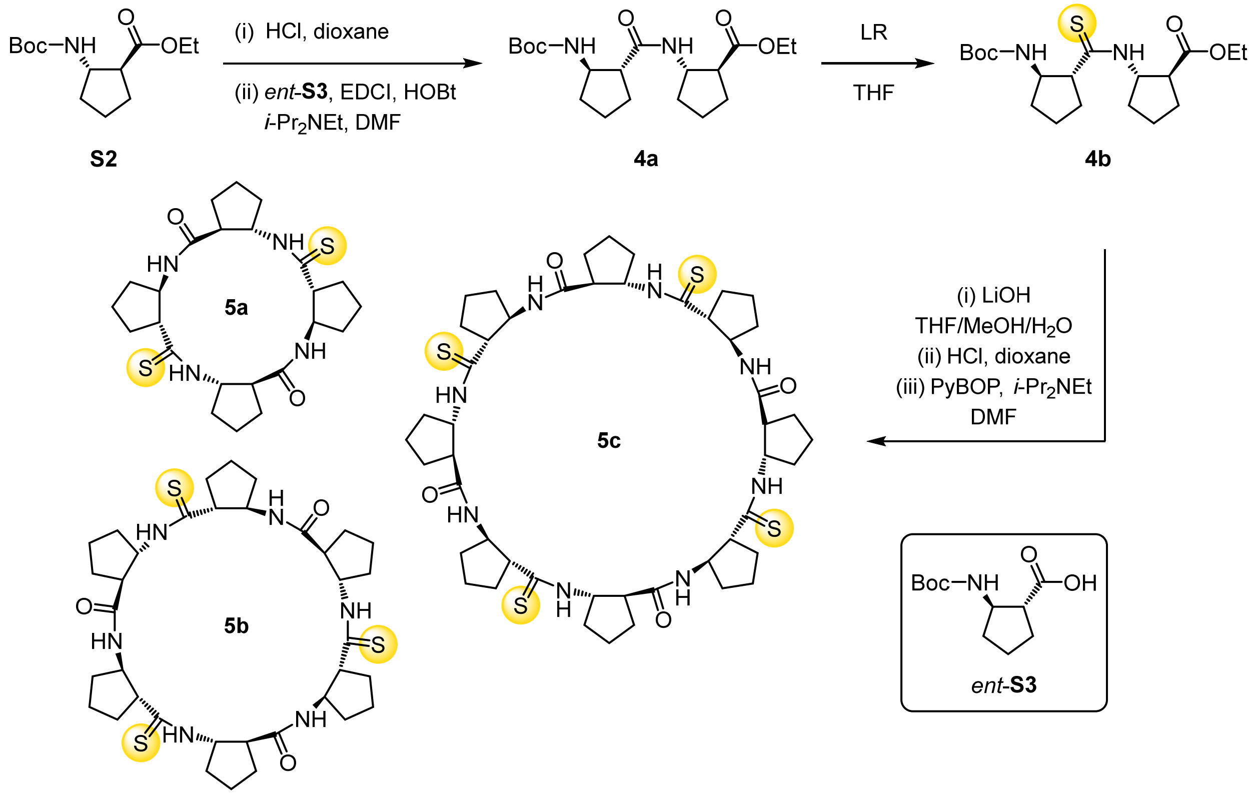

<u>Synthesis of **4a**</u>

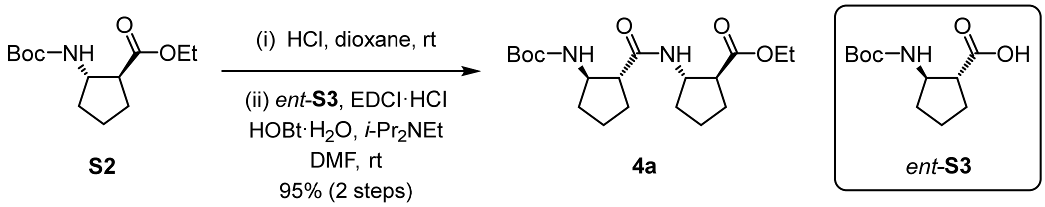

4a was synthesized from **S2** (1.00 g, 3.89 mmol) by following **general procedure B**. Purified by recrystallization from acetone/hexane (1/2, 90 mL), 1.36 g, 95 % (from **S2**), white solid.

^{1}H NMR (400 MHz, $CDCl_3$) δ 7.68 (s, 1H), 4.72 (d, J = 5.4 Hz, 1H), 4.39 (p, J = 7.0 Hz, 1H), 4.11 (q, J = 7.1 Hz, 2H), 3.92 (app. p, J = 7.1 Hz, 1H), 2.67–2.51 (m, 2H), 2.15–1.94 (m, 4H), 1.94–1.80 (m, 2H), 1.81–1.68 (m, 2H), 1.69–1.52 (m, 3H), 1.49–1.35 (m, 10H), 1.22 (t, J = 7.1 Hz, 3H).

^{13}C NMR (101 MHz, $CDCl_3$) δ 175.07, 173.38, 156.57, 80.25, 60.61, 56.46, 54.81, 53.48, 50.81, 33.79, 33.06, 28.70, 28.51, 27.69, 24.28, 23.44, 14.34.

HRMS (ESI) *m/z* calcd for $[M+Na]^+$: 391.2203, found: 391.2204.

Note: *ent-**S3** was prepared by following the analogous procedure with **S3**, starting from ethyl 2-oxocyclopentanecarboxylate and (R)-(+)-1-phenylethylamine.*[8,9]

Synthesis of **4b**

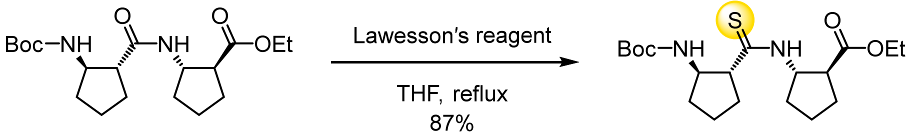

4a **4b**

4b was synthesized from **4a** (921.2 mg, 2.50 mmol) by following the **general procedure C**. Purified by flash column chromatography (CH_2Cl_2/Et_2O/hexane = 20/35/45), 831.9 mg, 87%, white solid.

^{1}H NMR (500 MHz, $CDCl_3$) δ 9.58 (s, 1H), 4.99 (p, J = 6.6 Hz, 1H), 4.85 (s, 1H), 4.07 (q, J = 7.1 Hz, 2H), 3.83 (t, J = 6.8 Hz, 1H), 2.90 (q, J = 8.5 Hz, 1H), 2.75 (q, J = 6.8 Hz, 1H), 2.33 (tt, J = 8.7, 4.3 Hz, 1H), 2.19 (dq, J = 14.3, 7.2 Hz, 1H), 2.07–1.92 (m, 3H), 1.85 (dq, J = 13.1, 7.2 Hz, 1H), 1.78–1.59 (m, 5H), 1.49–1.34 (m, 10H), 1.18 (t, J = 7.1 Hz, 3H).

^{13}C NMR (126 MHz, $CDCl_3$) δ 204.76, 174.72, 156.59, 80.24, 60.65, 60.51, 60.06, 57.79, 50.00, 33.22, 32.18, 31.59, 29.12, 28.36, 23.79, 23.64, 14.15.

HRMS (ESI) *m*/*z* calcd for $[M+Na]^+$: 407.1975, found: 407.1976.

Synthesis of **5a**, **5b**, and **5c**

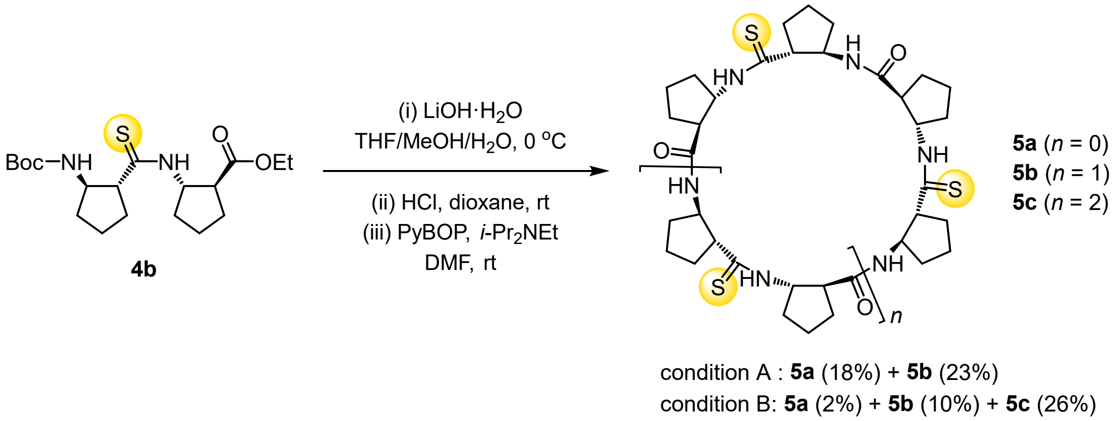

Hydrolysis and Boc deprotection: To a solution of **4b** (192.3 mg, 0.50 mmol) in THF/MeOH/H_2O (1/1/1, 5.0 mL) was added lithium hydroxide hydrate (104.9 mg, 2.5 mmol) at 0 °C. The mixture was stirred at the same temperature for 4.5 h, and the reaction was quenched by careful addition of 1.0 M aqueous HCl (until pH became 2–3). The aqueous layer was extracted with CH_2Cl_2 (3 × 5 mL), and the combined organic layer was dried over anhydrous $MgSO_4$ and concentrated under reduced pressure. The crude carboxylic acid was treated with HCl (4.0 N in 1,4-dioxane, 2.5 mL) and stirred at room temperature under nitrogen atmosphere for 1.5 h. After the reaction was complete (checked by TLC), all volatiles were removed under reduced pressure and the product was further dried under high vacuum to afford a fully deprotected dipeptide.

Amide coupling (condition A): The resulting solid was dispersed in DMF (70 mL), and PyBOP (265.4 mg, 0.51 mmol) was added to the mixture. Then *i*-Pr_2NEt (0.26 mL, 1.50 mmol) in DMF (30 mL) was added dropwise over 30 min. After 48 h of stirring at room temperature under nitrogen atmosphere, the reaction mixture was concentrated under reduced pressure and diluted with EtOAc (50 mL). The organic layer was washed with 1.0 M aqueous HCl (50 mL), H_2O (50 mL), brine (25 mL), and dried over anhydrous $MgSO_4$. The crude mixture was purified by flash chromatography (10/90 to 15/85 acetone/CH_2Cl_2) to afford **5a** (23.1 mg, 18% over 3 steps) and **5b** (27.2 mg, 23% over 3 steps), both as a white solid.

Amide coupling (condition B): The resulting solid was dispersed in DMF (10 mL), and PyBOP (265.4 mg, 0.51 mmol) was added to the mixture, then *i*-Pr_2NEt (0.26 mL, 1.50 mmol) was added dropwise over 5 min. After 36 h of stirring at room temperature under nitrogen atmosphere, the reaction mixture was diluted with EtOAc (50 mL). The organic layer was washed with 1.0 M aqueous HCl (50 mL), H_2O (50 mL), brine (25 mL), and dried over anhydrous $MgSO_4$. The crude mixture was purified by flash chromatography (10/90 to 20/80 acetone/CH_2Cl_2) to afford **5a** (2.7 mg, 2% over 3 steps), **5b** (9.8 mg, 5% over 3 steps), and **4c** (20.7 mg, 17% over 3 steps), as a white solid.

for **5a**

^{1}H NMR (500 MHz, 1/4 $CD_3OH/CDCl_3$) δ 8.97 (d, *J* = 9.2 Hz, 2H), 7.56 (d, *J* = 9.8 Hz, 2H), 5.44 (dt, *J* = 19.6, 10.3 Hz, 2H), 4.49 (qd, *J* = 10.3, 7.2 Hz, 2H), 3.44–3.32 (m, 2H, partially overlapped with CD_3OH), 2.89 (td, *J* = 12.0, 8.3 Hz, 2H), 2.45 (dtd, *J* = 12.4, 9.2, 8.7, 2.8 Hz, 2H), 2.26 (ddt, *J* = 12.3, 8.7, 4.5 Hz, 2H), 2.12 (ddt, *J* = 10.8, 8.1, 4.1 Hz, 2H), 2.03 (ddt, *J* = 10.9, 7.9, 4.1 Hz, 2H), 1.99–1.78 (m, 6H), 1.76–1.56 (m, 10H).

^{13}C NMR (126 MHz, 4/1 $CD_3OH/CDCl_3$) δ 204.21, 174.49, 63.38, 56.82, 53.69, 49.89, 31.71, 30.85, 29.44, 28.08, 20.79, 19.81.

HRMS (ESI) *m/z* calcd for $[M+H]^+$: 477.2352, found: 477.2354.

for **5b**

^{1}H NMR (500 MHz, 1/4 $CD_3OH/CDCl_3$) δ 10.00 (d, *J* = 8.5 Hz, 3H), 8.48 (d, *J* = 8.6 Hz, 3H), 5.15 (app. p, *J* = 8.1 Hz, 3H), 4.23 (app. p, *J* = 8.0 Hz, 3H), 2.89 (q, *J* = 8.6 Hz, 3H), 2.59 (q, *J* = 8.3 Hz, 3H), 2.28 (dtd, *J* = 13.0, 8.3, 4.9 Hz, 3H), 2.20–1.81 (m, 21H), 1.80–1.64 (m, 12H).

^{13}C NMR (126 MHz, 1/4 $CD_3OH/CDCl_3$) δ 204.03, 174.45, 62.94, 58.60, 53.77, 53.50, 31.98, 31.71, 30.81, 28.10, 22.44, 22.00.

HRMS (ESI) *m/z* calcd for $[M+H]^+$: 715.3492, found: 715.3499.

for **5c**

^{1}H NMR (500 MHz, 1/4 $CD_3OH/CDCl_3$) δ 10.20 (d, *J* = 7.8 Hz, 4H), 8.58 (d, *J* = 8.1 Hz, 4H), 4.99–4.90 (m, 4H), 4.21 (app. p, *J* = 7.7 Hz, 4H), 2.94 (q, *J* = 8.5 Hz, 4H), 2.63 (dt, *J* = 8.4, 6.1 Hz, 4H), 2.27 (dtd, *J* = 12.7, 7.9, 4.3 Hz, 4H), 2.15–1.94 (m, 20H), 1.94–1.69 (m, 24H).

^{13}C NMR (126 MHz, 1/4 $CD_3OH/CDCl_3$) δ 204.05, 174.57, 62.34, 60.52, 54.37, 52.99, 32.45, 31.99, 31.62, 27.40, 23.75, 22.41.

HRMS (ESI) *m/z* calcd for $[M+Na]^+$: 975.4452, found: 975.4450.

Scheme S4. Scalable synthesis of long thioamide β-peptides and their global desulfurization.

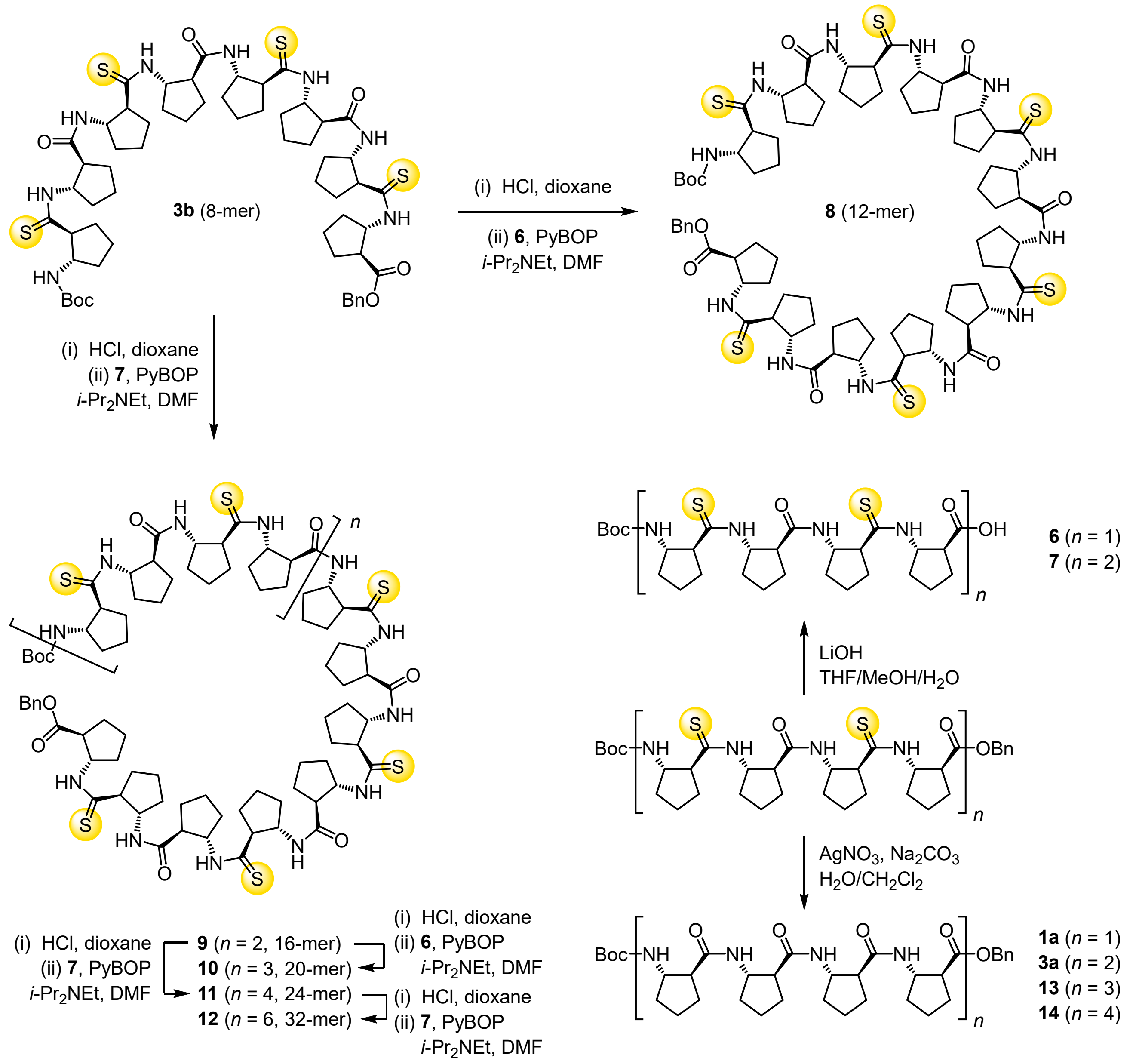

Synthesis of **6**

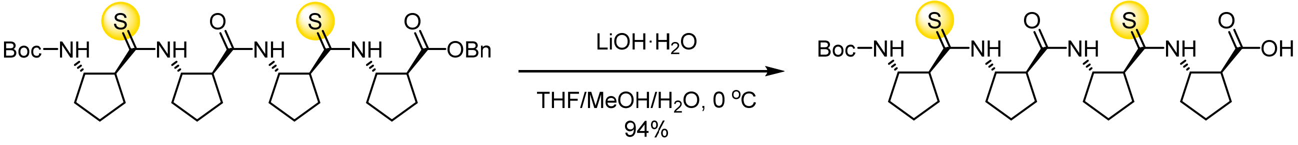

1e **6**

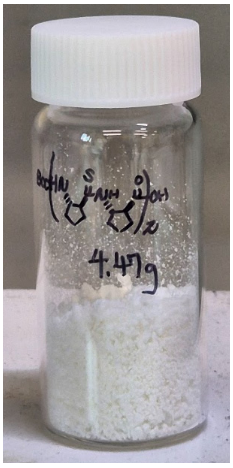

6 was synthesized from **1e** (5.50 g, 8.03 mmol) by following the **general procedure A (option B)**. Used for subsequent reaction without further purification, 4.47 g, 94 %, white solid. For analysis, a small amount of sample was recrystallized from MeOH/Et_2O.

^{1}H NMR (500 MHz, DMSO-d_6) δ 12.08 (s, 1H), 9.99–9.76 (m, 2H), 7.82 (d, J = 7.6 Hz, 1H), 6.98 (d, J = 8.0 Hz, 1H), 4.83–4.77 (m, 2H), 4.14 (app. p, J = 6.7 Hz, 1H), 4.06 (t, J = 6.9 Hz, 1H), 3.05 (q, J = 7.4 Hz, 1H), 2.92–2.77 (m, 2H), 2.58 (q, J = 6.7 Hz, 1H), 2.06–1.75 (m, 11H), 1.75 – 1.44 (m, 13H), 1.37 (s, 9H).

^{13}C NMR (126 MHz, DMSO-d_6) δ 205.65, 205.38, 175.76, 173.28, 155.31, 77.84, 59.73, 59.08, 59.04, 58.13, 57.95, 56.97, 50.54, 48.93, 32.96, 32.48, 32.23, 32.21, 31.55, 31.52, 29.32, 28.54, 28.26, 24.30, 23.93, 23.88, 23.67.

HRMS (ESI) m/z: $[M+Na]^+$ calcd for 617.2802, found: 617.2804.

Synthesis of 8-mer **3b**

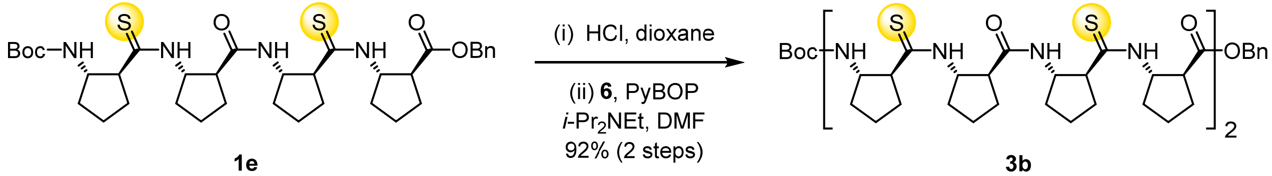

1e **3b**

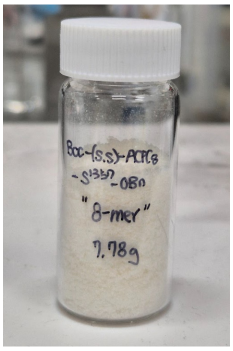

3b was synthesized from **1e** (5.00 g, 7.30 mmol) by following the **general procedure D (option B)**. Purified by flash column chromatography (EtOAc/Hexane = 6/4 to 7/3), 7.78 g, 92%, white solid.

Synthesis of **7**

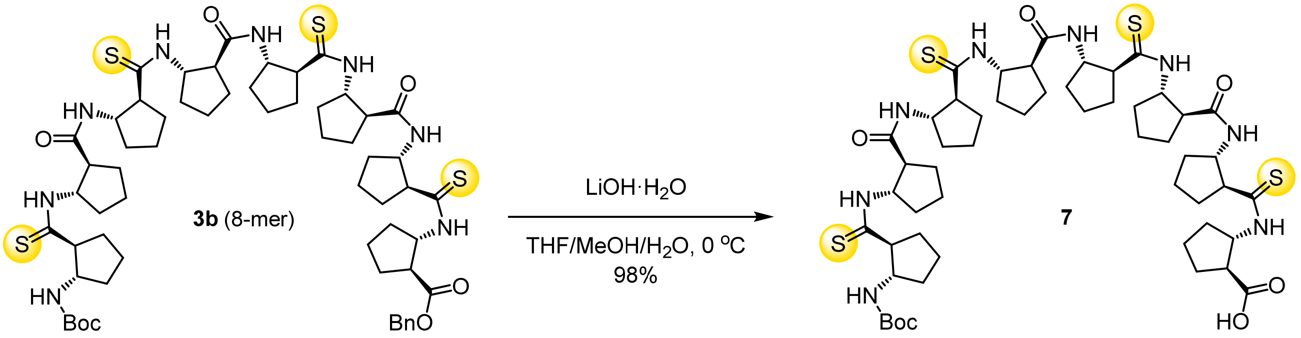

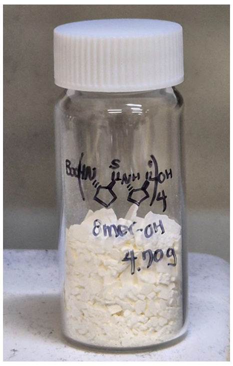

7 was synthesized from **3b** (5.20 g, 4.48 mmol) by following the **general procedure A (option B)**. Used for subsequent reaction without further purification, 4.70 g, 98%, white solid. For analysis, a small amount of sample was purified by flash column chromatography (5/95 MeOH/CH_2Cl_2).

^{1}H NMR (500 MHz, 4/1 $CDCl_3$/CD_3OH) δ 9.70 (d, J = 8.8 Hz, 1H), 9.50 (d, J = 7.7 Hz, 1H), 9.30 (d, J = 9.4 Hz, 1H), 9.21 (d, J = 9.5 Hz, 1H), 8.86 (d, J = 8.5 Hz, 1H), 8.58 (d, J = 8.3 Hz, 1H), 7.90 (d, J = 8.3 Hz, 1H), 6.02 (d, J = 8.3 Hz, 1H), 5.18–5.01 (m, 4H), 4.37–4.30 (m, 4H), 3.20–3.08 (m, 2H), 3.07–2.96 (m, 2H), 2.84 (q, J = 7.6 Hz, 1H), 2.74–2.65 (m, 2H), 2.50 (q, J = 7.4 Hz, 1H), 2.40–2.26 (m, 4H), 2.26–2.02 (m, 9H), 2.02–1.81 (m, 17H), 1.81–1.51 (m, 18H), 1.45 (s, 9H).

^{13}C NMR (126 MHz, 4/1 $CDCl_3$/CD_3OH) δ 208.37, 207.52, 206.62, 175.14, 174.84, 174.62, 156.67, 79.94, 61.44, 61.25, 61.14, 61.05, 60.96, 60.51, 59.86, 59.75, 58.45, 58.38, 58.07, 52.19, 51.51, 51.02, 50.19, 34.15, 33.76, 33.64, 33.55, 33.48, 32.94, 32.74, 31.97, 29.85, 29.69, 29.32, 29.12, 28.92, 28.59, 25.93, 25.90, 25.81, 25.70, 25.55, 25.52, 24.74, 24.11.

HRMS (ESI) m/z: $[M+Na]^+$ calcd for 1093.5082, found: 1093.5076.

Synthesis of 12-mer **8**

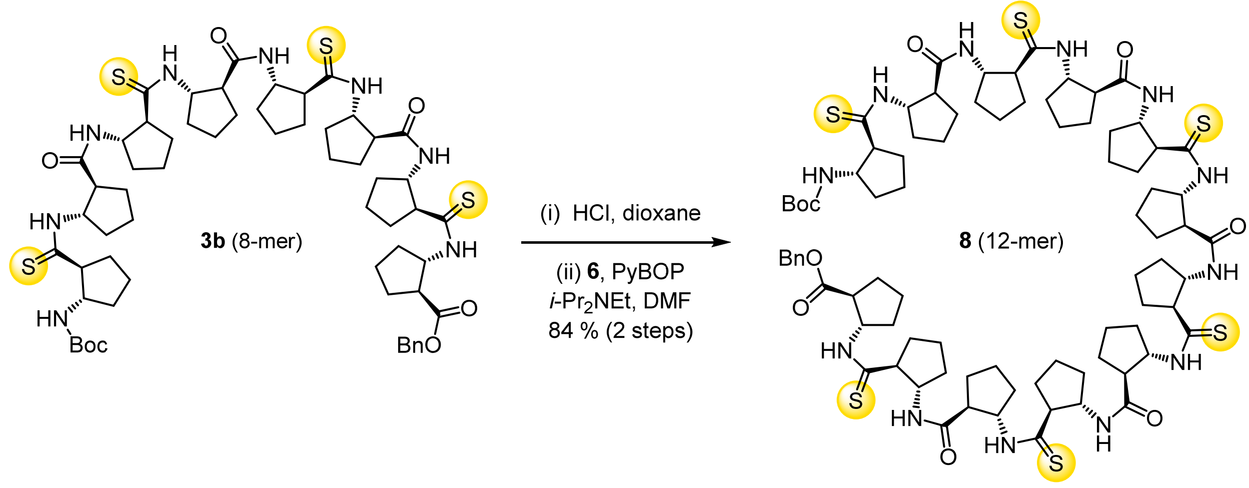

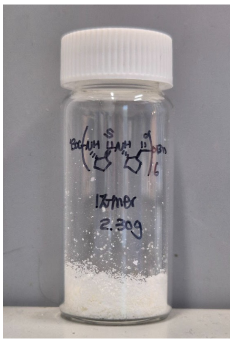

8 was synthesized from **3b** (1.50 g, 2.14 mmol) by following the **general procedure D (option B)**. Purified by flash column chromatography (2/4/4 CH_2Cl_2/EtOAc/hexane), 2.30 g, 84 % (from **3b**), white solid.

^{1}H NMR (500 MHz, $CDCl_3$) δ 10.12 (d, J = 7.1 Hz, 1H), 9.30–9.20 (m, 2H), 9.11 (d, J = 9.3 Hz, 1H), 9.04 (d, J = 9.8 Hz, 1H), 8.76–8.64 (m, 2H), 8.64–8.55 (m, 2H), 8.49 (d, J = 7.7 Hz, 1H), 7.66 (d, J = 8.3 Hz, 1H), 7.38–7.27 (m, 5H), 5.21–5.04 (m, 8H), 5.32 (d, J = 7.8 Hz, 1H), 4.40–4.26 (m, 4H), 4.11 (dt, J = 8.8, 4.4 Hz, 1H), 4.03 (app. p, J = 7.8 Hz, 1H), 3.24–3.13 (m, 2H), 3.08–2.98 (m, 3H), 2.94 (q, J = 7.8, 7.2 Hz, 1H), 2.85 (q, J = 7.5 Hz, 1H), 2.69–2.57 (m, 4H), 2.50–2.33 (m, 7H), 2.67–1.96 (m, 19H), 1.96–1.79 (m, 22H), 1.79–1.51 (m, 25H), 1.44 (s, 9H).

^{13}C NMR (126 MHz, $CDCl_3$) δ 207.46, 207.31, 207.26, 207.17, 206.93, 206.85, 175.63, 174.75, 174.41, 174.24, 174.09, 156.45, 136.18, 128.54, 128.05, 127.91, 80.09, 66.53, 62.06, 61.05, 60.96, 60.89, 60.80, 60.70, 60.19, 60.07, 59.33, 58.21, 58.07, 57.87, 52.13, 51.13, 50.87, 49.90, 34.34, 33.80, 33.72, 33.59, 33.52, 33.48, 33.44, 33.39, 33.29, 33.18, 32.73, 32.49, 32.22, 29.89, 29.38, 29.21, 29.16, 29.06, 28.85, 28.62, 25.85, 25.82, 25.76, 25.73, 25.68, 25.66, 25.62, 25.57, 25.29, 25.00, 24.40.

HRMS (ESI) m/z: $[M+Na]^+$ calcd for 1659.7831, found: 1659.7901.

*Note: ent-**8** for racemic crystallography was prepared by following the analogous procedure with **8**. Spectroscopic data were consistent with **8**.*

Synthesis of 16-mer **9**

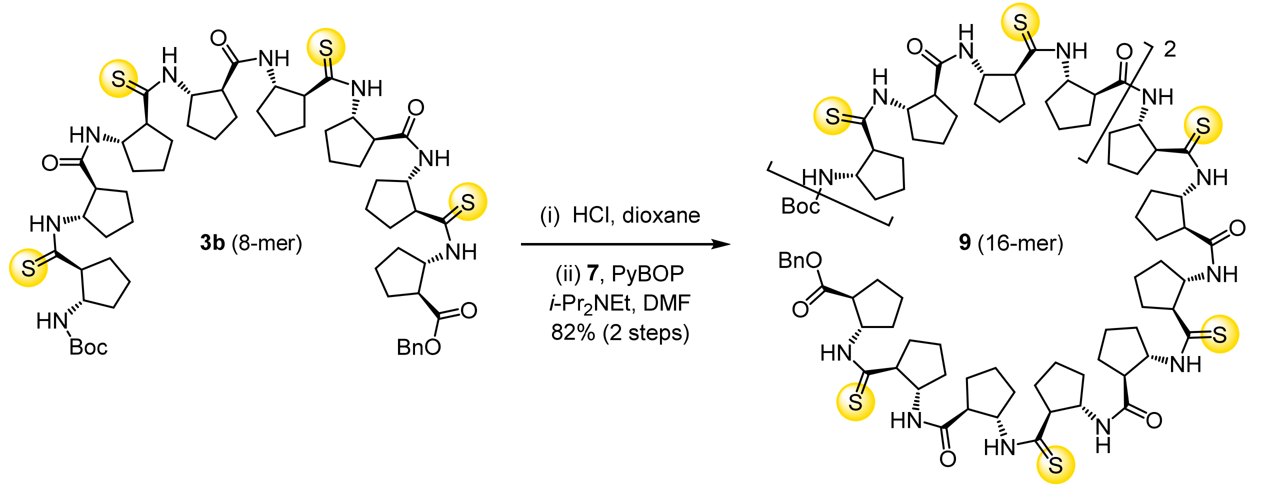

9 was synthesized from **3b** (2.30 g, 1.98 mmol) by following the **general procedure D (option C)**. Purified by flash column chromatography (acetone/CH_2Cl_2 = 5/95 to 8/92), 3.42 g, 82% (from **3b**), white solid.

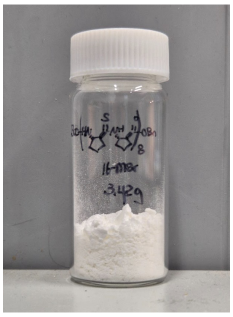

^{1}H NMR (500 MHz, $CDCl_3$) δ 10.09 (d, J = 6.9 Hz, 1H), 9.40–9.25 (m, 4H), 9.22–9.09 (m, 2H), 8.86 (d, J = 9.2 Hz, 1H), 8.83–8.75 (m, 3H), 8.73 (d, J = 8.4 Hz, 1H), 8.66 (d, J = 8.5 Hz, 1H), 8.54 (d, J = 7.6 Hz, 1H), 7.78 (d, J = 8.3 Hz, 1H), 7.36–7.27 (m, 5H), 5.57 (d, J = 8.2 Hz, 1H), 5.23–5.02 (m, 10H), 4.44–4.22 (m, 6H), 4.18–4.01 (m, 2H), 3.24–3.14 (m, 2H), 3.09–2.84 (m, 7H), 2.72–2.56 (m, 6H), 2.51–2.28 (m, 9H), 2.23–1.97 (m, 24H), 1.96–1.52 (m, 64H), 1.42 (s, 9H).

^{13}C NMR (126 MHz, $CDCl_3$) δ 207.47, 207.33, 207.29, 207.18, 206.99, 206.87, 175.60, 174.73, 174.40, 174.29, 174.25, 174.07, 156.45, 136.19, 128.53, 128.03, 127.89, 80.07, 66.50, 62.18, 61.08, 61.01, 60.97, 60.91, 60.89, 60.79, 60.67, 60.21, 60.13, 59.27, 58.20, 58.20, 58.06, 57.91, 52.12, 51.13, 50.84, 49.88, 34.34, 33.80, 33.75, 33.58, 33.53, 33.49, 33.42, 33.31, 33.17, 32.80, 32.42, 32.19, 29.84, 29.36, 29.18, 29.18, 29.05, 28.85, 28.62, 25.86, 25.82, 25.75, 25.73, 25.70, 25.67, 25.62, 25.57, 25.31, 25.06, 24.37.

HRMS (ESI) m/z: $[M+Na]^+$ calcd for 2136.0110, found: 2136.0100.

*Note: ent-**9** for racemic crystallography was prepared by following the analogous procedure with **9**. Spectroscopic data were consistent with **9**.*

Synthesis of 20-mer **10**

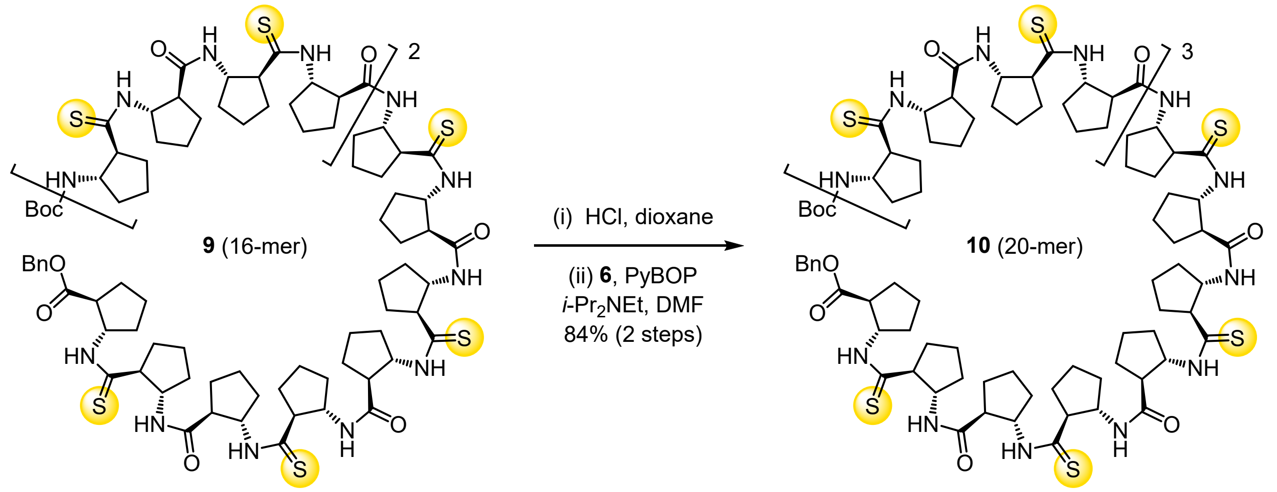

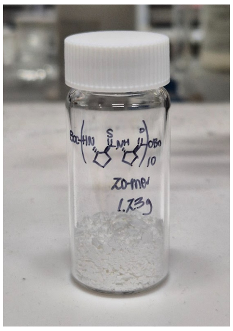

10 was synthesized from **9** (1.20 g, 0.567 mmol) by following the **general procedure D (option C)**. Purified by flash column chromatography (acetone/$CHCl_3$ = 5/95 to 10/90), 1.23 g, 84% (from **9**), white solid.

^{1}H NMR (500 MHz, $CDCl_3$) δ 10.13 (d, *J* = 7.0 Hz, 1H), 9.42–9.20 (m, 6H), 9.12 (d, *J* = 9.4 Hz, 1H), 9.04 (d, *J* = 9.6 Hz, 1H), 8.85–8.73 (m, 5H), 8.71 (d, *J* = 8.5 Hz, 1H), 8.61 (d, *J* = 8.4 Hz, 1H), 8.55 (d, *J* = 9.4 Hz, 1H), 8.50 (d, *J* = 7.7 Hz, 1H), 7.66 (d, *J* = 8.4 Hz, 1H), 7.38–7.26 (m, 5H), 5.31 (d, *J* = 7.8 Hz, 1H), 5.25–5.04 (m, 12H), 4.45–4.25 (m, 8H), 4.10 (app. p, *J* = 6.4 Hz, 1H), 4.03 (app. p, *J* = 7.2 Hz, 1H), 3.24–3.14 (m, 2H), 3.10–2.98 (m, 6H), 2.95 (q, *J* = 7.3 Hz, 1H), 2.85 (q, *J* = 6.4 Hz, 1H), 2.71–2.59 (m, 8H), 2.51–2.32 (m, 11H), 2.25–1.97 (m, 31H), 1.97–1.61 (m, 80H), 1.45 (s, 9H).

^{13}C NMR (126 MHz, $CDCl_3$) δ 207.50, 207.41, 207.38, 207.34, 207.21, 207.03, 206.85, 175.58, 174.77, 174.37, 174.34, 174.27, 174.09, 156.51, 136.27, 128.55, 128.03, 127.94, 80.20, 66.51, 62.37, 61.11, 61.10, 61.06, 61.00, 60.82, 60.69, 60.25, 60.14, 59.29, 58.25, 58.10, 57.97, 52.16, 51.18, 50.90, 50.84, 49.90, 34.39, 33.82, 33.77, 33.62, 33.58, 33.54, 33.45, 33.39, 33.20, 32.84, 32.39, 32.22, 29.31, 29.24, 29.22, 29.08, 28.88, 28.64, 25.90, 25.85, 25.79, 25.76, 25.67, 25.64, 25.51, 25.35, 25.11, 24.36.

HRMS (ESI) *m/z*: $[M+Na+H]^{2+}$ calcd for 1307.1248, found: 1307.1208.

Synthesis of 24-mer **11**

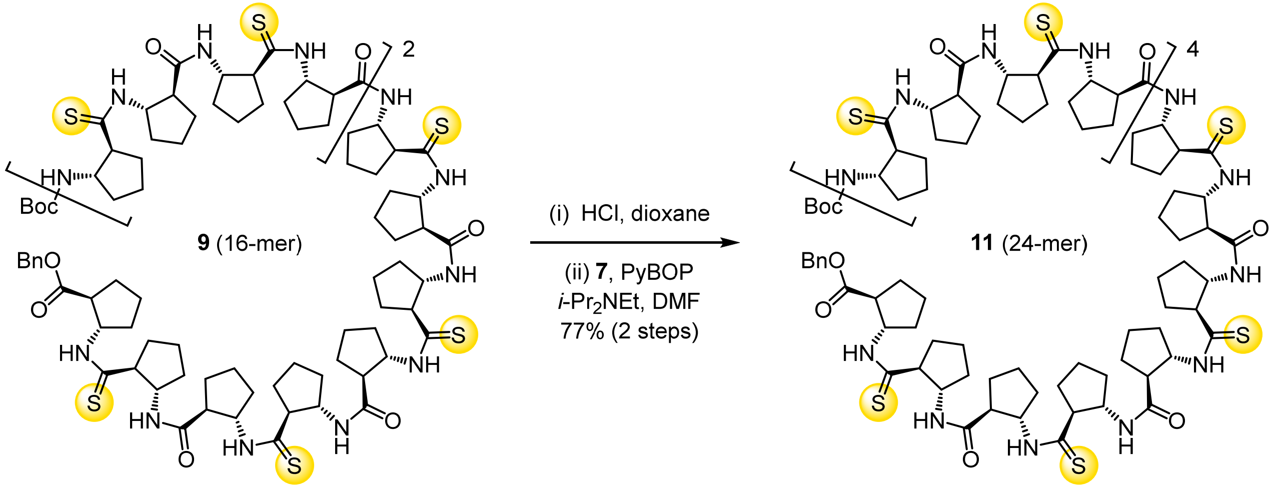

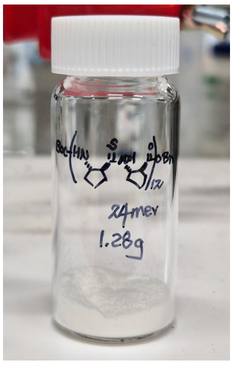

11 was synthesized from **9** (1.15 g, 0.544 mmol) by following the **general procedure D (option C)**. Purified by flash column chromatography (acetone/$CHCl_3$ = 5/95 to 10/90), 1.28 g, 77% (from **9**), white solid.

^{1}H NMR (500 MHz, $CDCl_3$) δ 10.16 (d, J = 7.2 Hz, 1H), 9.41–9.29 (m, 6H), 9.29–9.22 (m, 2H), 9.09 (d, J = 9.2 Hz, 1H), 8.96 (d, J = 9.7 Hz, 1H), 8.86–8.72 (m, 7H), 8.69 (d, J = 8.5 Hz, 1H), 8.58 (d, J = 8.5 Hz, 1H), 8.47 (d, J = 7.5 Hz, 1H), 8.34 (d, J = 9.4 Hz, 1H), 7.57 (d, J = 8.2 Hz, 1H), 7.40–7.27 (m, 5H), 5.22–5.04 (m, 15H), 4.42–4.27 (m, 10H), 4.12–4.04 (m, 1H), 4.04–3.95 (m, 1H), 3.24–3.13 (m, 2H), 3.11–2.97 (m, 9H), 2.92–2.88 (m, 1H), 2.80 (q, J = 7.1 Hz, 1H), 2.71–2.59 (m, 10H), 2.53–2.31 (m, 14H), 2.21–1.98 (m, 37H), 1.96–1.79 (m, 50H), 1.80–1.65 (m, 44H), 1.46 (s, 9H).

^{13}C NMR (126 MHz, $CDCl_3$) δ 207.50, 207.49, 207.48, 207.45, 207.42, 207.39, 207.33, 207.18, 206.94, 206.68, 175.42, 174.85, 174.38, 174.36, 174.33, 174.28, 174.22, 174.07, 174.05, 156.64, 136.52, 128.53, 128.10, 127.94, 80.81, 66.49, 61.48, 61.17, 61.11, 61.10, 61.05, 61.03, 60.93, 60.91, 60.77, 60.65, 60.34, 60.05, 59.24, 58.33, 58.15, 52.22, 51.30, 51.05, 50.99, 50.91, 50.90, 49.86, 34.49, 33.88, 33.82, 33.72, 33.66, 33.63, 33.59, 33.55, 33.54, 33.50, 33.38, 33.30, 33.20, 32.88, 32.27, 32.07, 29.85, 29.51, 29.27, 29.19, 29.08, 29.03, 28.90, 28.65, 25.95, 25.91, 25.80, 25.76, 25.63, 25.57, 25.41, 25.28, 25.11, 24.22.

MALDI-TOF MS m/z: $[M+Na]^+$ calcd for 3088.4670, found: 3088.3708.

Synthesis of 32-mer **12**

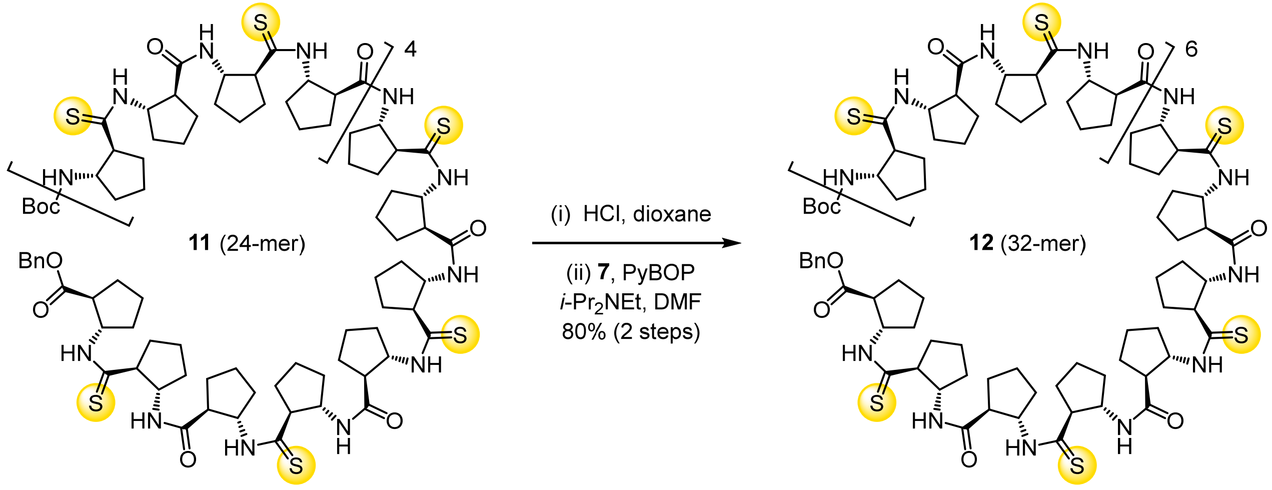

12 was synthesized from **11** (250 mg, 0.0815 mmol) by following the **general procedure D (option C)**. The reaction mixture of the amide coupling step was stirred at room temperature for 88 h and then concentrated under reduced pressure. The mixture was dispersed in MeOH (20 mL), centrifuged (5000 rpm, 3 min), and the supernatant was discarded. The resulting solid was washed twice with MeOH (20 mL) and twice with $CHCl_3$/MeOH (1/4, MeOH), by repeating the dispersion-centrifugation sequence under the same conditions to afford **12** (261 mg, 80%) as a white solid.

MALDI-TOF MS *m*/*z*: $[M+Na]^+$ calcd for 4040.9229, found: 4040.6185.

Note: The compound showed extremely low solubility in all types of organic solvents (including CH_2Cl_2, $CHCl_3$, THF, MeOH, EtOH, DMF, DMSO, NMP, and their mixtures), making NMR analysis challenging.

Synthesis of **1a**

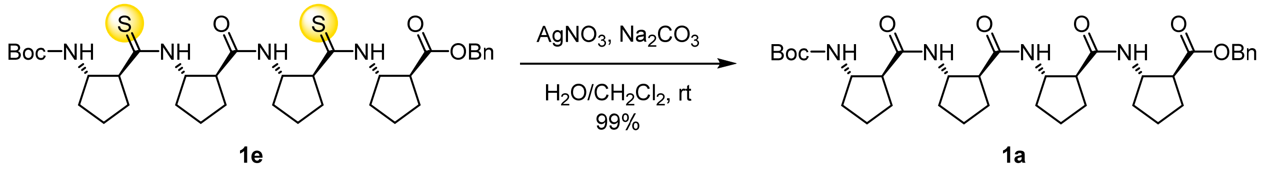

1a was prepared from **1e** (68.5 mg, 0.100 mmol) by following the **general procedure E**. 65.3 mg, 99%, white solid. Spectroscopic data were consistent with those previously reported.[9]

Synthesis of **3a**

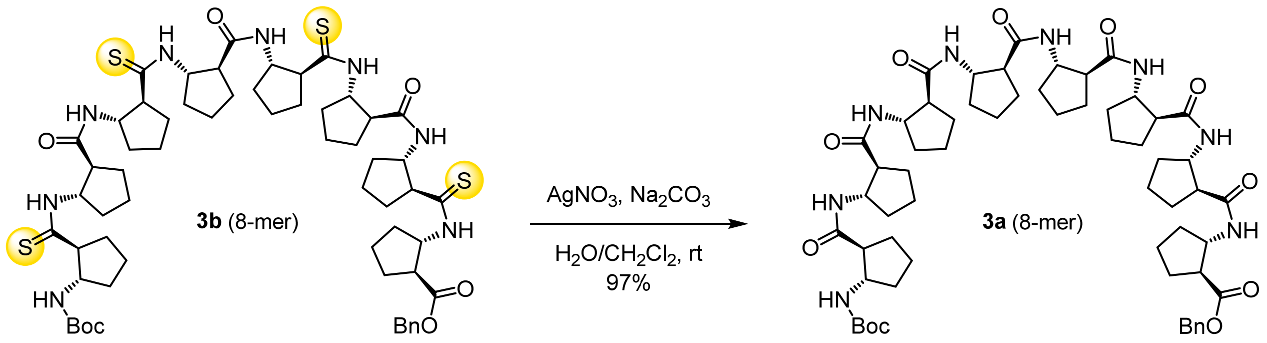

3a was prepared from **3b** (116.2 mg, 0.100 mmol) by following the **general procedure E**. 106.5 mg, 97%, white solid. Spectroscopic data were consistent with those previously reported.[9]

Synthesis of **13**

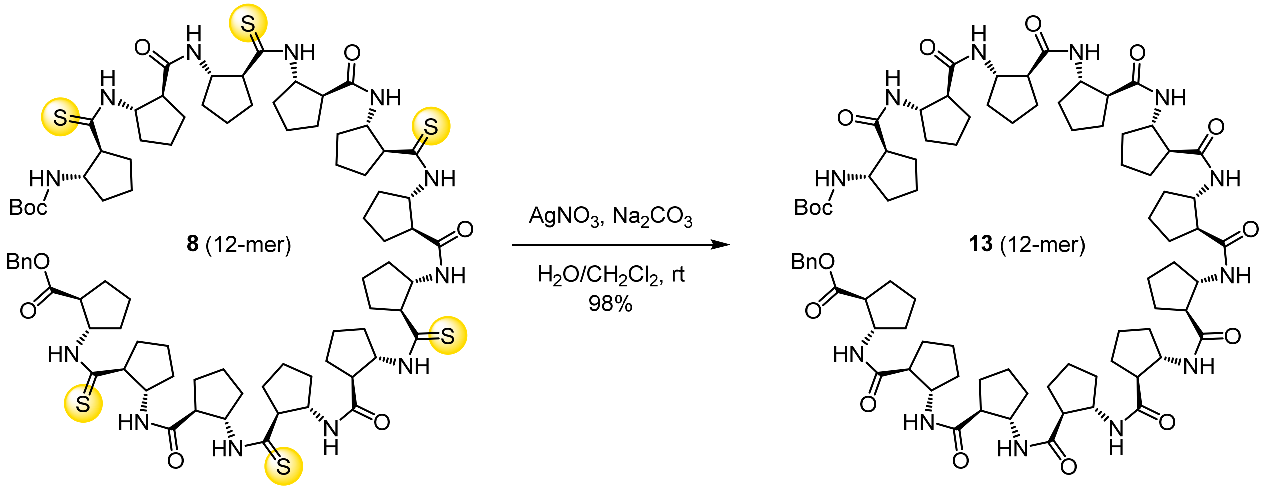

13 was prepared from **8** (163.8 mg, 0.100 mmol) by following the **general procedure E**. 151.3 mg, 98%, white solid.

^{1}H NMR (500 MHz, $CDCl_3$) δ 8.86–8.48 (m, 9H), 7.75 (d, *J* = 9.2 Hz, 1H), 7.44 (d, *J* = 8.2 Hz, 1H), 7.37–7.24 (m, 5H), 6.13 (d, *J* = 8.7 Hz, 1H), 5.13 (ABq, *J* = 12.6 Hz, 2H), 4.51 (app. p, *J* = 5.7 Hz, 1H), 4.38–4.17 (m, 10H), 4.05 (app. p, *J* = 8.7 Hz, 1H), 3.09 (dt, *J* = 8.9, 5.8 Hz, 1H), 2.70 (q, *J* = 7.7 Hz, 1H), 2.60–2.37 (m, 9H), 2.23 (q, *J* = 7.7 Hz, 1H), 2.16–1.88 (m, 25H), 1.88–1.50 (m, 47H), 1.42 (s, 9H).

^{13}C NMR (500 MHz, $CDCl_3$) δ 175.71, 175.48, 175.12, 175.03, 175.00, 174.96, 174.83, 156.55, 136.53, 128.52, 127.98, 127.96, 79.51, 66.26, 57.44, 55.55, 55.51, 55.45, 55.44, 55.33, 54.69, 54.40, 52.51, 52.43, 52.40, 52.36, 52.34, 52.29, 52.20, 52.10, 50.00, 33.64, 33.48, 33.24, 33.21, 33.13, 33.08, 32.57, 29.63, 29.45, 28.82, 28.70, 28.63, 28.60, 28.18, 25.16, 24.95, 24.82, 24.79, 24.71, 24.64, 23.83, 23.19.

HRMS (ESI) *m/z*: $[M+Na+H]^{2+}$ calcd for 782.4637, found: 782.4628.

*Note: ent-**13** for racemic crystallography was prepared by following the analogous procedure with **13**. Spectroscopic data were consistent with **13**.*

Synthesis of **14**

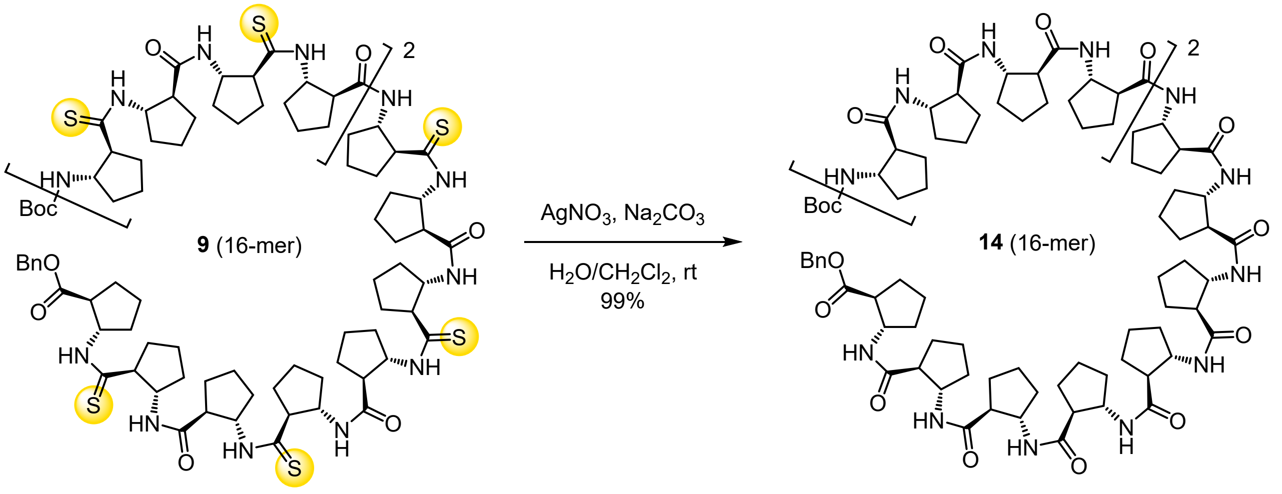

14 was prepared from **9** (105.8 mg, 0.050 mmol) by following the **general procedure E**. 98.1 mg, 99%, white solid.

^{1}H NMR (500 MHz, $CDCl_3$) δ 8.82–8.59 (m, 11H), 8.54 (d, *J* = 8.8 Hz, 1H), 8.43 (d, *J* = 9.0 Hz, 1H), 7.67 (d, *J* = 9.2 Hz, 1H), 7.39–7.27 (m, 5H), 6.80 (d, *J* = 8.2 Hz, 1H), 5.53 (d, *J* = 8.4 Hz, 1H), 5.25–5.04 (ABq, *J* = 12.6 Hz, 2H), 4.52 (s, 1H), 4.38–4.21 (m, 14H), 4.05 (app. p, *J* = 8.5 Hz, 1H), 3.10 (q, *J* = 6.9 Hz, 1H), 2.76 (q, *J* = 7.9, 7.4 Hz, 1H), 2.26–1.89 (m, 38H), 1.86–1.57 (m, 58H), 1.44 (s, 9H).

^{13}C NMR (126 MHz, $CDCl_3$) δ 175.84, 175.72, 175.69, 175.10, 175.00, 174.89, 174.86, 156.54, 136.53, 128.53, 128.03, 127.98, 79.62, 77.42, 77.16, 76.91, 66.32, 57.40, 55.55, 55.47, 55.37, 54.75, 54.31, 52.50, 52.38, 52.28, 52.07, 50.02, 33.64, 33.61, 33.56, 33.35, 33.33, 33.30, 33.22, 33.15, 33.11, 33.07, 33.06, 33.03, 32.58, 31.70, 29.82, 29.59, 29.48, 28.84, 28.74, 28.64, 28.20, 25.11, 24.93, 24.92, 24.83, 24.73, 24.67, 23.87, 23.21, 22.77.

HRMS (ESI) *m*/*z*: $[M+Na+H]^{2+}$ calcd for 1004.6006, found: 1004.5971.

3. Supplementary Figures

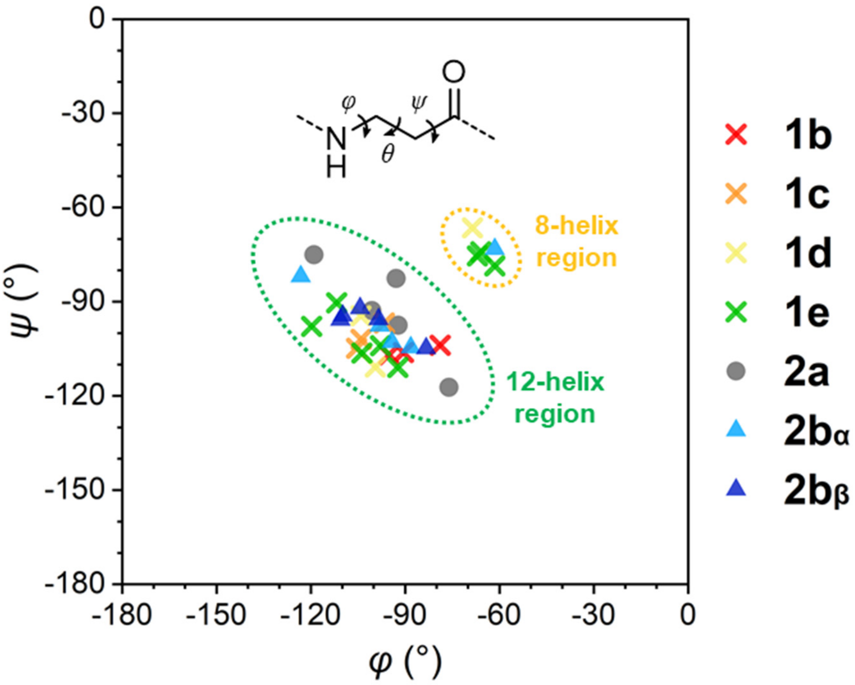

Figure S1. Backbone torsion angle plot found in crystal structures of β-tetra and β-hexapeptide series. Two non-helical *C*-terminus residues were omitted.

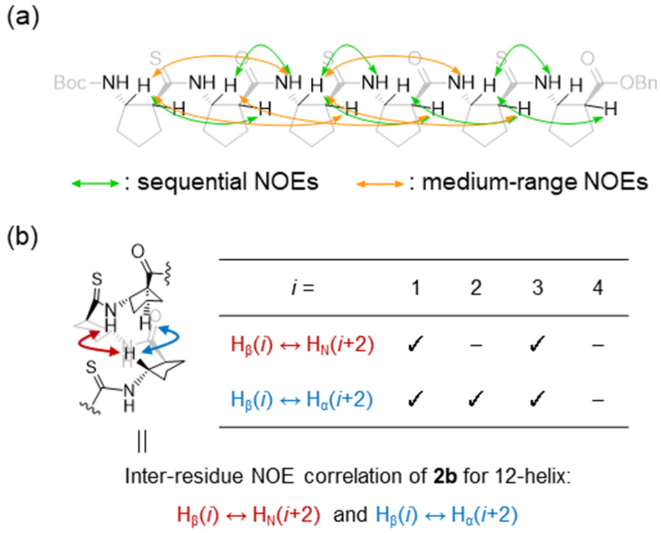

Figure S2. (a) Observed sequential and medium two-dimensional (2D) nuclear Overhauser effect (NOE) signals for **2b** in pyridine-d_5. (b) Cartoon showing characteristic inter-residue NOE correlation ($H_\beta(i)$↔$H_N(i+2)$ and $H_\beta(i)$↔$H_\alpha(i+2)$) found for conventional 12-helical β-peptides. The table shows observed NOE signals. For a detailed assignment of 2D NOE analysis, see the Supporting Information section 6.2.

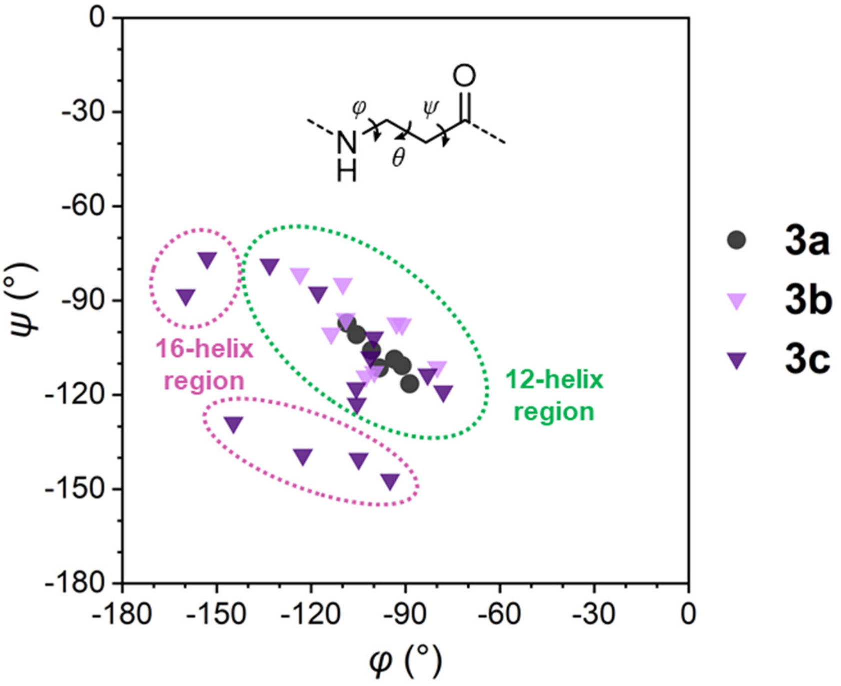

Figure S3. Backbone torsion angle plot found in crystal structures of β-octapeptide series. Two non-helical *C*-terminus residues were omitted.

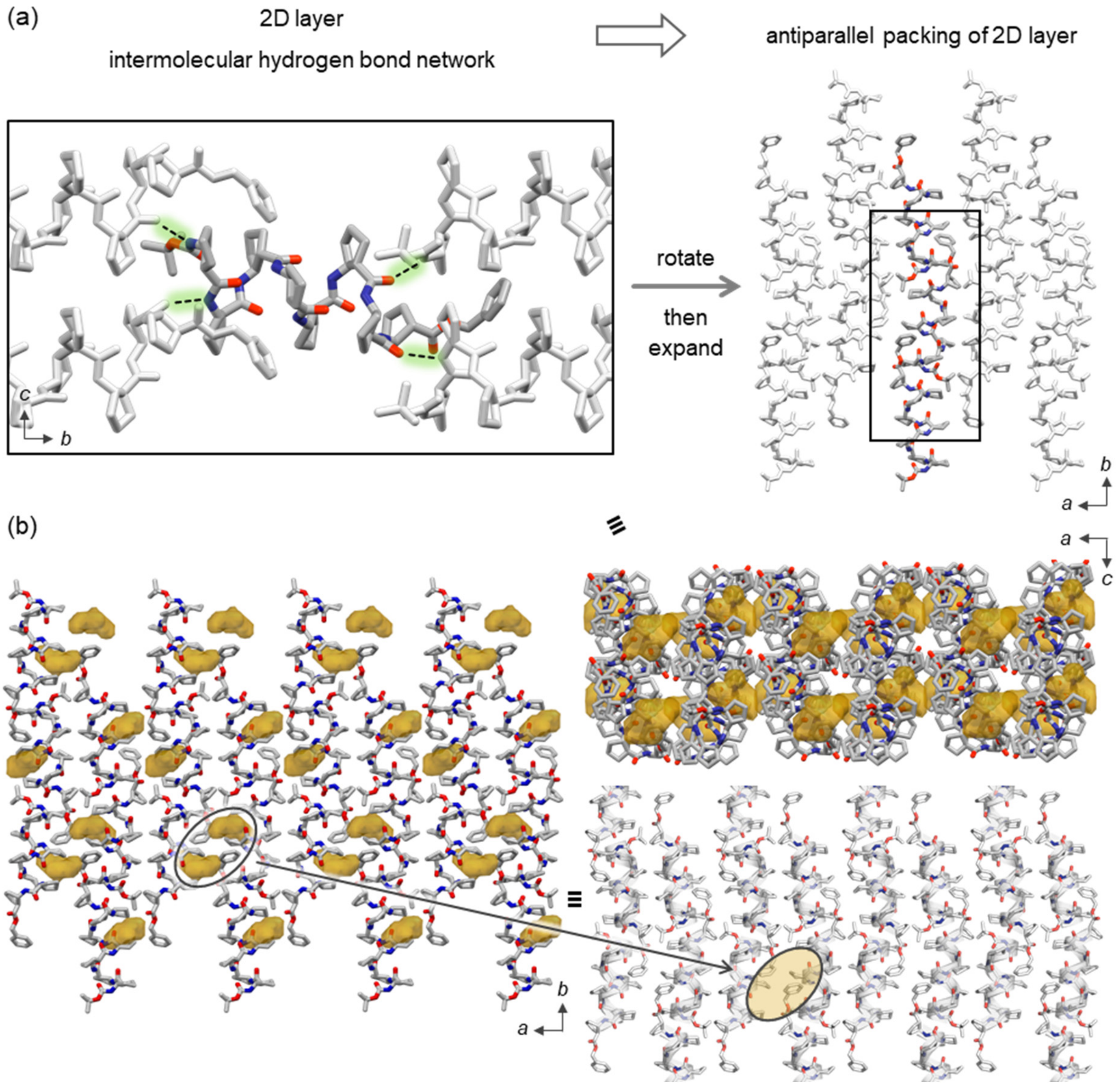

Figure S4. (a) Molecular packing mode of **3a**. The black dotted lines represent intermolecular hydrogen bonds. Disorders were omitted for clarity. (b) Expanded packing structure and void space (3.0% porosity) observed from the crystal structure of **3a**. Yellow surfaces represent void spaces.

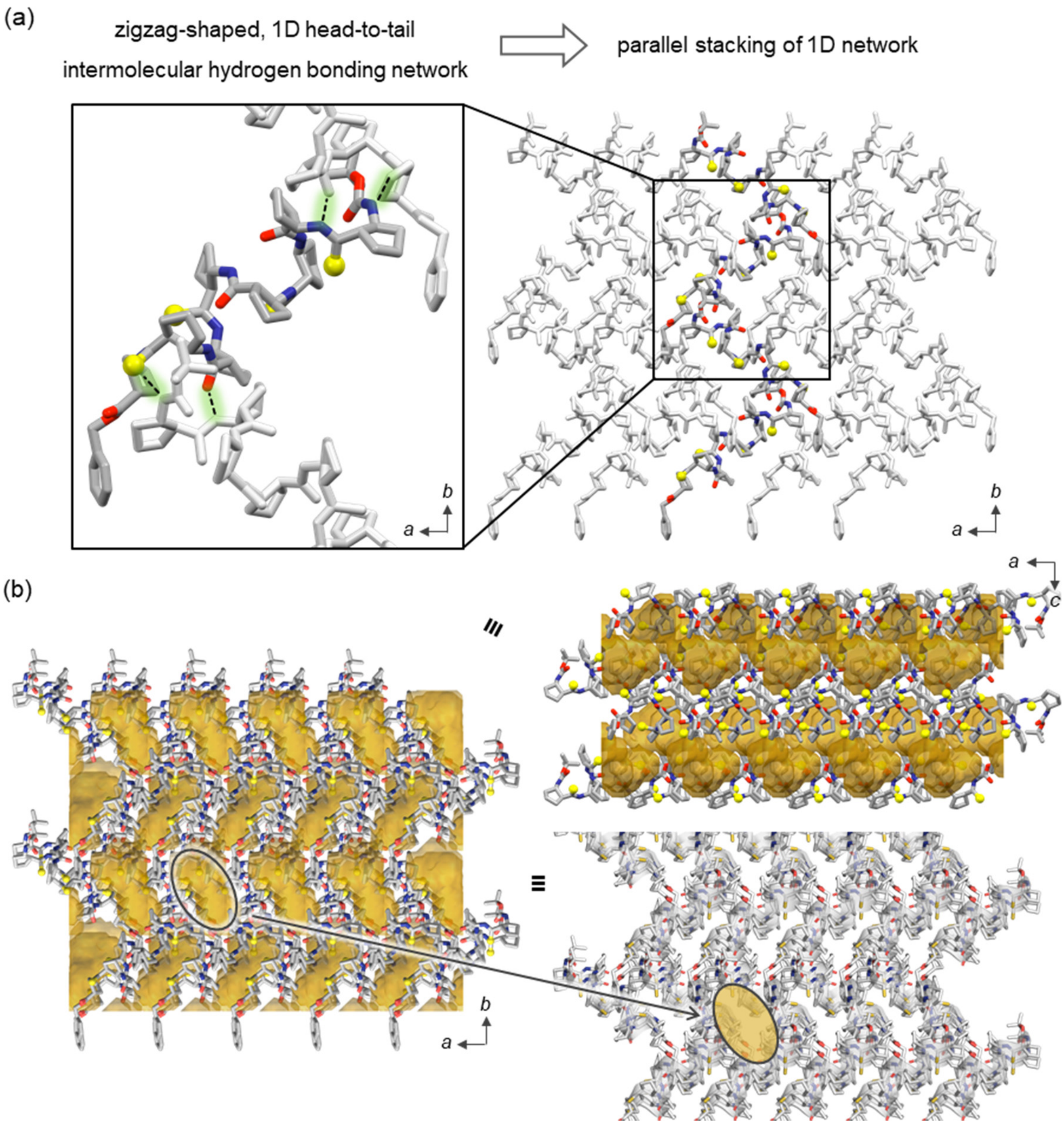

Figure S5. (a) Molecular packing mode of **3b**. The black dotted lines represent intermolecular hydrogen bonds. Disorders and solvent molecules were omitted for clarity. (b) Expanded packing structure and void space (16.1% porosity) observed from the crystal structure of **3b**. Yellow surfaces represent void spaces.

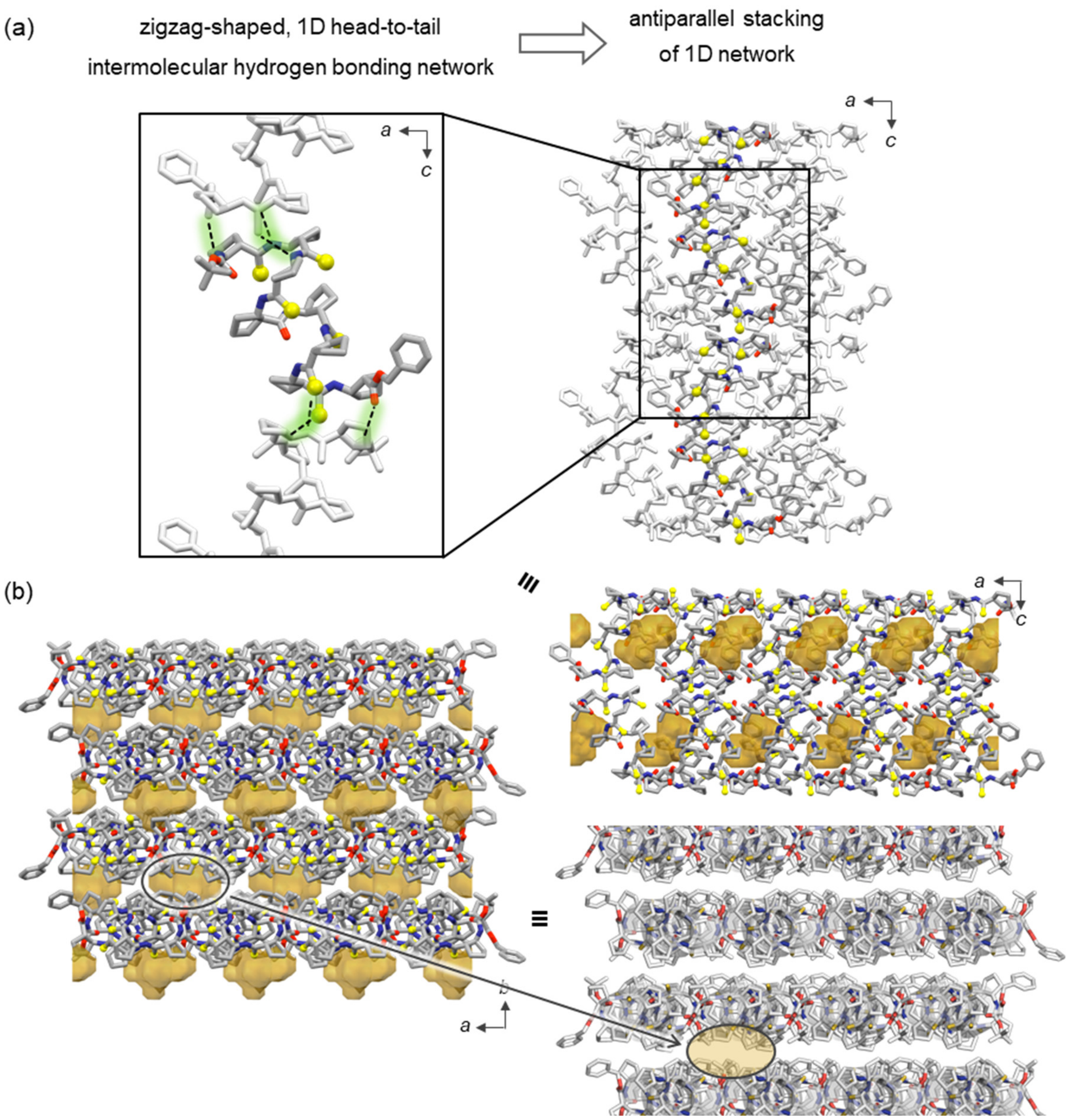

Figure S6. Molecular packing structure of **3c**. The black dotted lines represent intermolecular hydrogen bonds. Disorders and solvent molecules were omitted for clarity. (b) Expanded packing structure and void space (3.6% porosity) observed from the crystal structure of **3b**. Yellow surfaces represent void spaces.

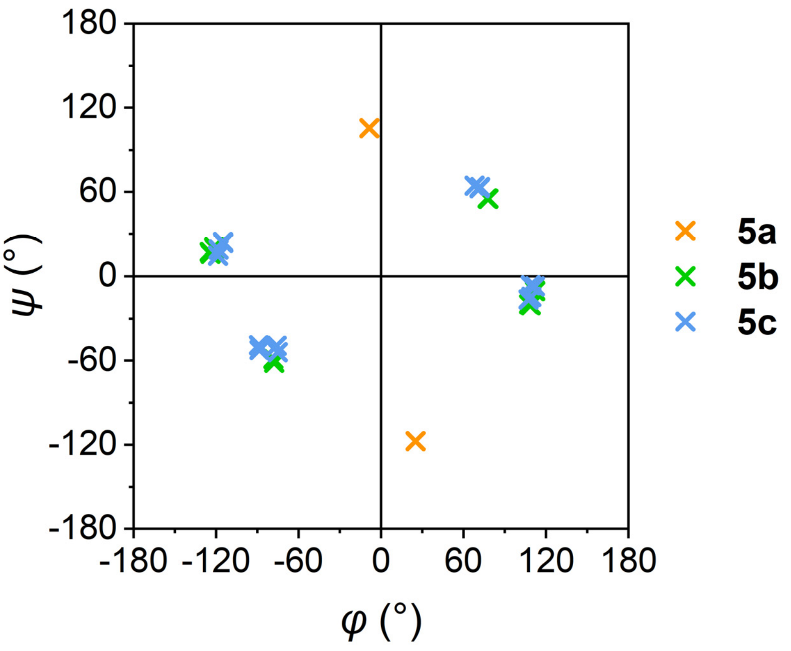

Figure S7. Backbone torsion angle plot found in the crystal structures of cyclic thioamide β-peptide series **5a**–**5c**.

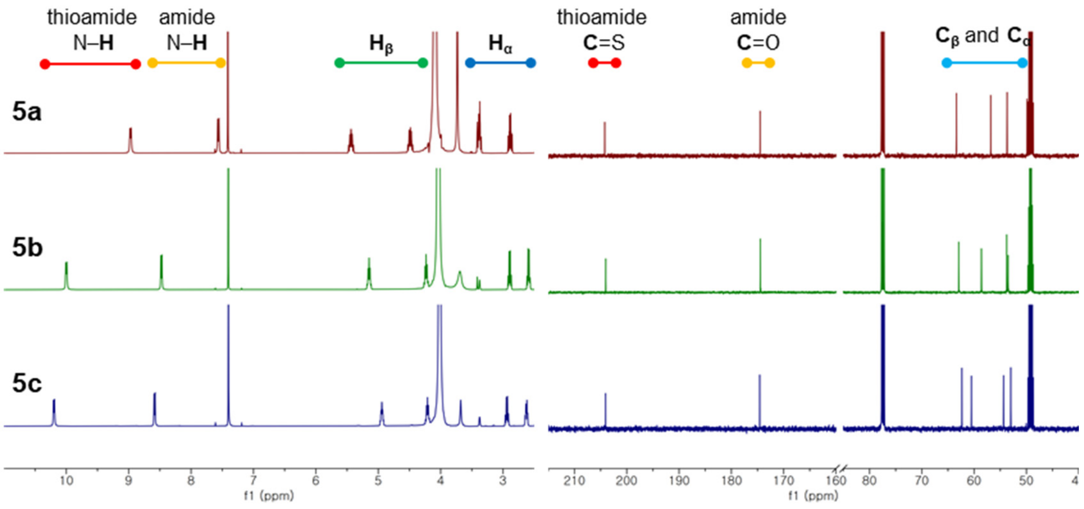

Figure S8. Partial ^{1}H (left) and ^{13}C (right) NMR spectra of **5a**–**5c** (4/1 $CDCl_3$/CD_3OH solution, v/v).

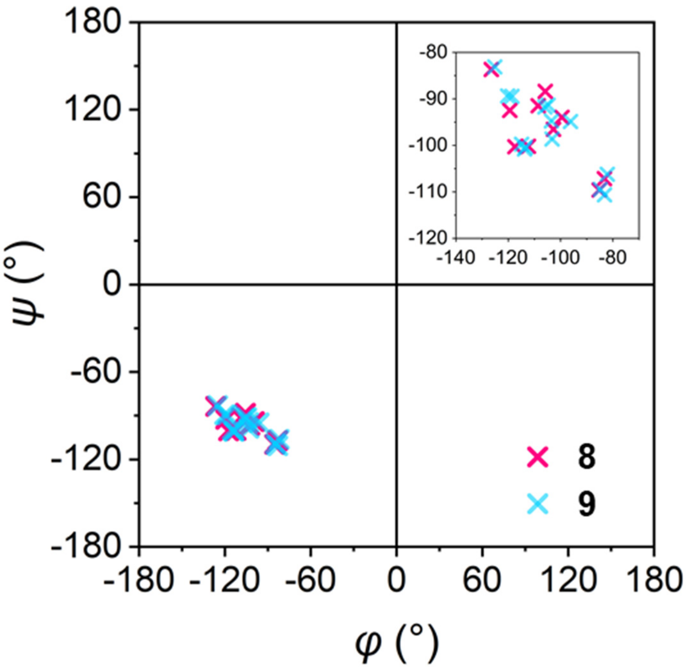

Figure S9. Backbone torsion angle plot found in the crystal structures of long thioamide β-peptides 12-mer **8** and 16-mer **9**. Two non-helical *C*-terminus residues were omitted. (inset) Expanded backbone torsion angle plot. The unit cell of the crystal structure contains a pair of enantiomeric molecules related by inversion symmetry(space group $P2_1/c$). Torsion angles are reported only for the **8** and **9**.

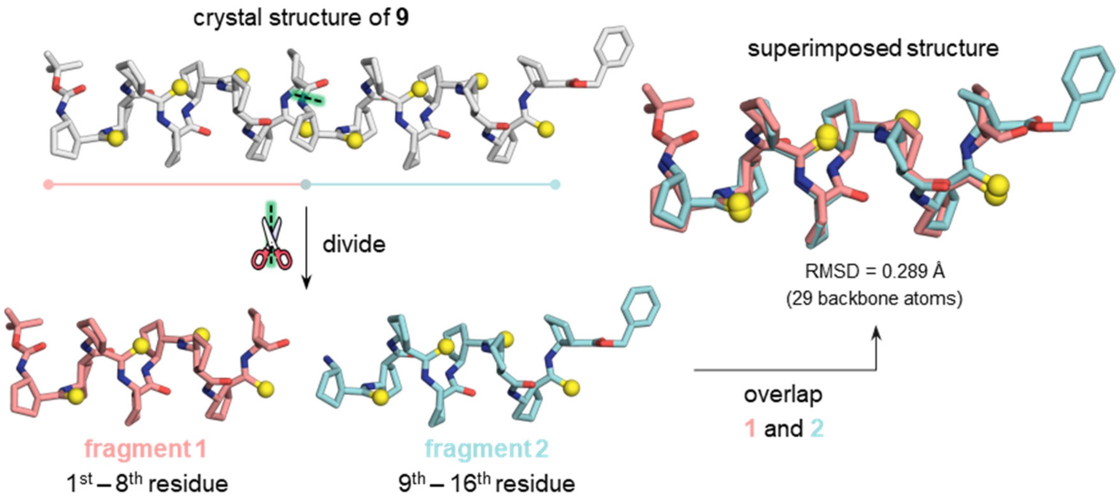

Figure S10. Superimposed structure between two fragments observed in the crystal structure of 16-mer **9**.

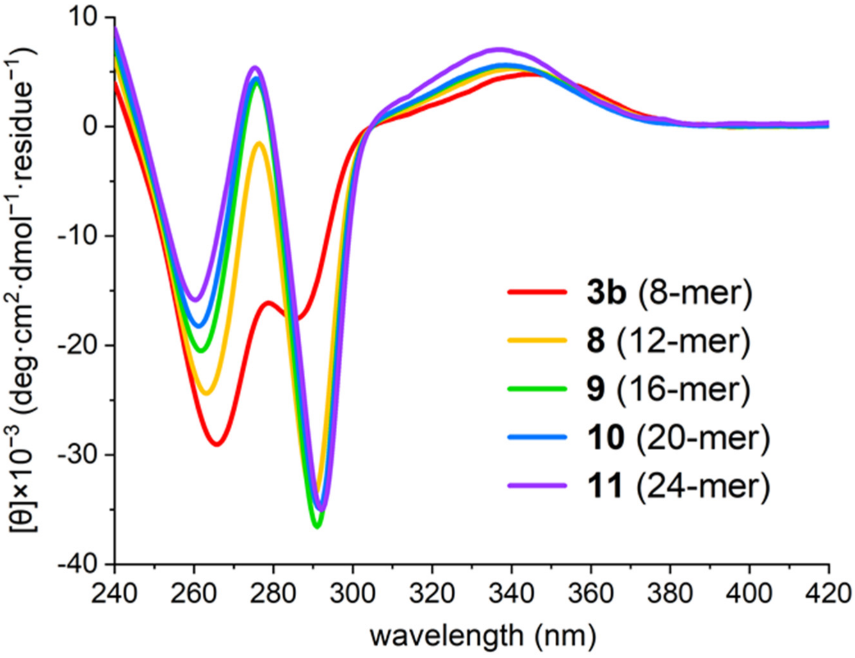

Figure S11. CD profiles for the long thioamide β-peptides (0.2 mM $CHCl_3$ solution).

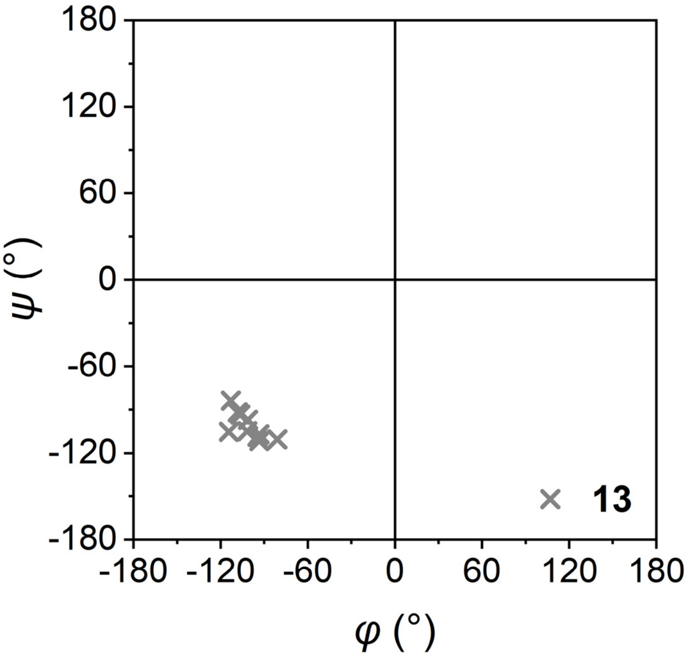

Figure S12. Backbone torsion angle plot found in the crystal structure of long β-peptide 12-mer **13**. Two non-helical *C*-terminus residues were omitted. (inset) Expanded backbone torsion angle plot. The unit cell of the crystal structure contains a pair of enantiomeric molecules related by inversion symmetry(space group *P*−1). Torsion angles are reported only for the **13**.

4. Supplementary Tables

Table S1. Optimization of amide coupling conditions for thioamide-containing fragments.

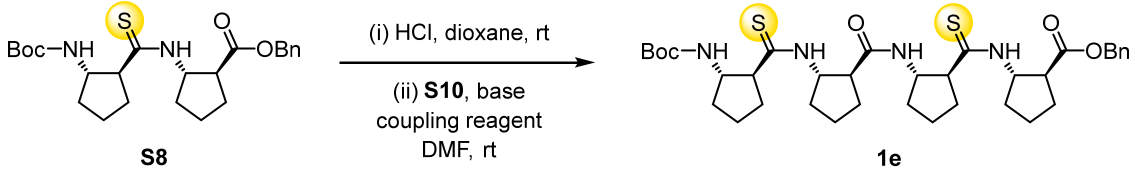

Entry [a]	coupling reagents (equiv.)	base (equiv.)	yield (2 steps, %) [b]
1	EDCI·HCl (1.3), HOBt·H_2O (1.3)	*i*-Pr_2NEt (3.0)	61
2	DEPBT (1.2)	*i*-Pr_2NEt (2.5)	51
3	CDMT (1.3)	NMM (4.0)	62
4[c]	NHS (1.3), HATU (1.3)	DMAP (0.1), *i*-Pr_2NEt (3.0)	9
5	PyBOP (1.2)	*i*-Pr_2NEt (2.5)	75
6	PyBOP (1.2)	collidine (3.0)	73
7	PyBOP (1.5)	*i*-Pr_2NEt (3.0)	44
8	PyBOP (1.1)	*i*-Pr_2NEt (2.5)	89

[a] Reaction conditions: (i) **S8** (0.05 mmol, 1.0 equiv.), 4.0 N HCl in dioxane (1.0 mL);
(ii) **S10** (1.1 equiv.), coupling reagent, base, DMF (1.0 mL), rt.

[b] Isolated yield. [c] CH_2Cl_2 was used as a co-solvent.

DEPBT = 3-(diethoxyphosphoryloxy)-1,2,3-benzotriazin-4(3*H*)-one;
CDMT = 2-chloro-4,5-dimethoxy-1,3,5-triazine;
NHS = *N*-hydroxysuccinimide;
HATU = 1-[bis(dimethylamino)methylene]-1*H*-1,2,3-triazolo[4,5-*b*]pyridinium-3-oxide hexafluorophosphate.

Table S2. Backbone torsion angle of β-tetrapeptides **1a**–**1e** and β-hexapeptides **2a**–**2b**.

peptide	residue	φ (°)	θ (°)	ψ (°)
**1a**[a]	1st	−83.29	87.02	−126.56
	2nd	−86.71	101.10	−104.38
	3rd	−145.63	115.78	−87.53
	4th	−166.92	88.99	−156.66
**1b**	1st	−95.10	89.98	−106.94
	2nd	−90.53	93.09	−106.19
	3rd	−78.87	95.43	−103.89
	4th	−145.88	95.38	−157.39
**1c**	1st	−104.03	87.78	−101.97
	2nd	−105.45	113.13	−104.84
	3rd	−96.59	97.99	−96.80
	4th	−162.43	125.17	−165.64
**1d**	1st	−99.47	87.23	−110.81
	2nd	−104.07	115.34	−94.49
	3rd	−68.59	131.76	−66.45
	4th	−95.46	73.63	−83.62
**1e**[b]	1-1st	−97.80	85.39	−104.02
	1-2nd	−111.79	105.27	−90.36
	1-3rd	−65.91	127.46	−74.10
	1-4th	−100.48	81.40	−100.45
	2-1st	−119.72	67.68	−97.84
	2-2nd	−85.69	112.70	−111.91
	2-3rd	−61.48	131.44	−78.60
	2-4th	−95.84	72.57	−99.38
	3-1st	−103.74	82.30	−106.35
	3-2nd	−92.33	109.94	−110.95
	3-3rd	−66.99	132.05	−75.42
	3-4th	−91.45	76.71	−100.29

peptide	residue	φ (°)	θ (°)	ψ (°)
**2a**	1st	−119.03	87.59	−75.10
	2nd	−92.25	77.85	−97.53
	3rd	−92.89	105.96	−82.63
	4th	−100.56	79.66	−92.88
	5th	−76.04	82.49	−117.25
	6th	−124.66	86.95	40.95
**2b**$_\alpha$	1st	−110.59	82.40	−95.79
	2nd	−104.32	108.47	−92.08
	3rd	−83.30	78.39	−104.87
	4th	−109.85	101.00	−94.43
	5th	−98.43	100.96	−95.56
	6th	−152.89	108.21	−164.48
**2b**$_\beta$	1st	−88.19	108.92	−104.53
	2nd	−123.21	105.14	−81.91
	3rd	−98.08	89.73	−97.62
	4th	−94.22	105.31	−102.87
	5th	−61.50	122.99	−73.27
	6th	−109.39	98.34	−84.04

[a] Reference 10. [b] Backbone torsion angles of three molecules in the asymmetric unit were displayed.

Table S3. Backbone torsion angle of β-octapeptides **3a**–**3c**.

peptide	residue	φ (°)	θ (°)	ψ (°)
**3a**	1st	−88.67	98.78	−116.45
	2nd	−93.44	88.59	−108.70
	3rd	−100.74	101.60	−105.92
	4th	−105.53	95.01	−100.85
	5th	−98.32	92.52	−111.42
	6th	−91.05	88.20	−110.77
	7th	−108.55	111.11	−97.15
	8th	−78.84	154.81	−165.28
**3b**[a]	1-1st	−102.63	83.16	−114.10
	1-2nd	−91.06	119.41	−97.57
	1-3rd	−109.89	89.36	−84.64
	1-4th	−108.95	98.34	−95.84
	1-5th	−79.88	88.75	−111.10
	1-6th	−123.61	111.73	−81.49
	1-7th	−113.57	98.75	−100.47
	1-8th	−80.41	151.96	95.30
	2-1st	−99.91	85.26	−112.66
	2-2nd	−92.83	119.17	−97.24
	2-3rd	−107.83	87.37	−88.69
	2-4th	−108.03	100.50	−92.67
	2-5th	−82.96	84.95	−107.83
	2-6th	−120.89	117.29	−84.31
	2-7th	−116.58	100.05	−99.09
	2-8th	−82.64	157.57	98.77

peptide	residue	φ (°)	θ (°)	ψ (°)
**3c**	1-1st	−104.87	127.17	−140.33
	1-2nd	−159.85	96.86	−88.20
	1-3rd	−122.60	97.61	−139.07
	1-4th	−105.45	82.62	−122.77
	1-5th	−82.95	102.77	−113.47
	1-6th	−117.77	102.87	−87.41
	1-7th	−100.01	89.58	−101.71
	1-8th	−147.38	105.59	−141.83
	2-1st	−94.89	111.59	−146.94
	2-2nd	−152.99	98.71	−76.43
	2-3rd	−144.64	97.67	−128.82
	2-4th	−105.65	81.63	−117.84
	2-5th	−77.98	102.74	−118.88
	2-6th	−133.16	105.81	−78.53
	2-7th	−101.14	85.23	−108.01
	2-8th	−152.55	120.82	−159.59

[a] Backbone torsion angles of two molecules in the asymmetric unit were displayed.

Table S4. Hydrogen bond lengths (distance between carbonyl O/S and N atom) found in the crystal structures of **3a**–**3c**.

peptide	hydrogen bond	bond length (Å)
**3a**	C=O(0)↔H–N(3)	2.83
	C=O(1)↔H–N(4)	2.75
	C=O(2)↔H–N(5)	2.79
	C=O(3)↔H–N(6)	2.80
	C=O(4)↔H–N(7)	2.78
	C=O(5)↔H–N(8)	2.83
**3b**-1 [a]	C=O(0)↔H–N(3)	2.95
	C=S(1)↔H–N(4)	3.37
	C=O(2)↔H–N(5)	2.82
	C=S(3)↔H–N(6)	3.47
	C=O(4)↔H–N(7)	2.80
	C=S(5)↔H–N(8)	3.42
**3b**-2 [a]	C=O(0)↔H–N(3)	2.95
	C=S(1)↔H–N(4)	3.38
	C=O(2)↔H–N(5)	2.83
	C=S(3)↔H–N(6)	3.44
	C=O(4)↔H–N(7)	2.82
	C=S(5)↔H–N(8)	3.39

peptide	hydrogen bond	bond length (Å)
**3c**-1 [a]	C=O(0)↔H–N(4)	2.90
	C=S(1)↔H–N(5)	3.49
	C=S(3)↔H–N(5)	3.46
	C=O(4)↔H–N(7)	2.82
	C=S(5)↔H–N(8)	3.37
**3c**-2 [a]	C=O(0)↔H–N(4)	2.86
	C=S(1)↔H–N(5)	3.58
	C=S(3)↔H–N(5)	3.30
	C=O(4)↔H–N(7)	2.94
	C=S(5)↔H–N(8)	3.36

[a] Hydrogen bond lengths of two molecules in the asymmetric unit were displayed.

Table S5. Backbone torsion angles of **5a**–**5c**.

peptide	residue	φ (°)	θ (°)	ψ (°)
**5a**	1st	−8.49	−67.74	105.48
	2nd	25.10	62.01	−117.46
	3rd	−8.49	−67.74	105.48
	4th	25.10	62.01	−117.46
**5b**[a]	1-1st	−124.10	72.09	15.89
	1-2nd	78.14	−124.67	55.14
	1-3rd	−121.02	71.94	19.55
	1-4th	108.78	−66.68	−20.41
	1-5th	−77.80	137.70	−61.56
	1-6th	111.85	−68.96	−10.18
	1-1st	−121.37	72.17	20.43
	1-2nd	107.74	−66.69	−19.87
	1-3rd	−78.44	136.99	−58.91
	1-4th	112.14	−68.45	−10.46
	1-5th	−124.61	72.71	17.11
	1-6th	77.38	−126.03	55.33

peptide	residue	φ (°)	θ (°)	ψ (°)
**5c**[a]	1-1st	−75.89	137.31	−49.54
	1-2nd	107.32	−68.38	−16.52
	1-3rd	−114.78	64.03	23.62
	1-4th	72.00	−126.17	63.36
	1-5th	−88.84	132.73	−49.17
	1-6th	108.80	−71.94	−6.43
	1-7th	−118.70	63.15	14.55
	1-8th	71.65	−125.23	62.78
	2-1st	−75.19	137.93	−54.01
	2-2nd	109.26	−69.43	−14.64
	2-3rd	−115.08	63.79	24.30
	2-4th	71.58	−128.68	62.45
	2-5th	−88.46	135.41	−52.54
	2-6th	111.88	−70.49	−7.18
	2-7th	−118.29	61.27	18.87
	2-8th	68.03	−125.84	64.32

[a] Backbone torsion angles of two molecules in the asymmetric unit were displayed.

Table S6. Backbone torsion angles of *rac*-**8** and *rac*-**9**.

peptide	residue	φ (°)	θ (°)	ψ (°)
rac-**8**	1st	−102.71	93.10	−96.54
	2nd	−105.84	102.37	−88.40
	3rd	−83.22	81.06	−107.16
	4th	−112.32	113.78	−100.20
	5th	−108.56	100.92	−91.47
	6th	−119.41	99.10	−92.49
	7th	−85.21	92.81	−109.59
	8th	−117.44	106.58	−100.29
	9th	−99.49	98.88	−93.97
	10th	−126.49	107.24	−83.68
	11th	−84.44	85.38	−113.89
	12th	−75.86	132.77	−176.11

peptide	residue	φ (°)	θ (°)	ψ (°)
rac-**9**	1st	−103.25	95.22	−98.63
	2nd	−104.76	103.50	−91.35
	3rd	−82.12	82.28	−106.23
	4th	−114.89	111.31	−99.76
	5th	−103.49	98.24	−94.75
	6th	−118.60	101.19	−89.46
	7th	−84.80	87.76	−109.48
	8th	−113.42	106.83	−100.40
	9th	−105.99	101.94	−91.74
	10th	−120.23	99.91	−89.37
	11th	−83.18	90.94	−110.65
	12th	−113.80	104.91	−100.80
	13th	−96.21	99.90	−94.87
	14th	−125.17	106.30	−83.12
	15th	−85.66	84.90	−109.39
	16th	−69.53	121.31	−179.44

Note: The unit cell of the crystal structure contains a pair of enantiomeric molecules related by inversion symmetry(space group $P2_1/c$). Torsion angles are reported only for the ***8*** *and* ***9****.*

Table S7. Backbone torsion angles of *rac*-**13**.

peptide	residue	φ (°)	θ (°)	ψ (°)
rac-**13**	1st	−101.07	93.23	−97.09
	2nd	−80.83	77.52	−110.59
	3rd	−112.94	107.38	−83.80
	4th	−101.61	80.19	−104.97
	5th	−93.24	99.04	−107.01
	6th	−108.19	98.44	−91.91
	7th	−106.56	87.22	−93.42
	8th	−114.23	92.02	−105.33
	9th	−93.31	102.38	−111.71
	10th	−94.60	85.30	−108.75
	11th	−109.60	111.20	−86.21
	12th	−86.77	137.88	−163.11

Note: The unit cell of the crystal structure contains a pair of enantiomeric molecules related by inversion symmetry(space group P−1). Torsion angles are reported only for the ***13****.*

5. Crystallography

5.1. Crystallization Methods

Single crystals suitable for X-ray diffraction experiments were grown by the following methods. Afforded single crystals were mounted in inert oil and transferred to the cold gas stream of the diffractometer.

Compound	Crystallization Method	CCDC Number
**S8**	recrystallized from CH_2Cl_2/Et_2O/hexane	2447589
**S10**	slow evaporation of MeOH/CH_2Cl_2 solution	2447572
**1b**	vapor diffusion of hexane toward EtOAc solution	2004043
**1c**	recrystallized from EtOAc	1967170
**1d**	recrystallized from MeOH	2002611
**1e**	recrystallized from MeOH	1975177
**2a**	slow evaporation from H_2O/MeOH solution	2129890
**2b**$_\alpha$	vapor diffusion of pentane toward EtOAc solution	2021289
**2b**$_\beta$	vapor diffusion of pentane toward $CHCl_3$ solution	2041888
**3a**	slow evaporation from MeOH/CH_2Cl_2 solution	2129889
**3b**	vapor diffusion of pentane toward EtOAc solution	2048422
**3c**	slow evaporation from EtOAc/CH_2Cl_2 solution	2048513
**5a**	slow evaporation from MeOH/CH_2Cl_2 solution	2350730
**5b**	recrystallized from $CHCl_3$/Et_2O/heptane	2435172
**5c**	vapor diffusion of pentane toward $CHCl_3$ solution	2447552
rac-**8**	vapor diffusion of Et_2O toward $CHCl_3$ solution	2213750
rac-**9**	vapor diffusion of Et_2O toward $CHCl_3$ solution	2256299
rac-**13**	slow evaporation from MeOH/CH_2Cl_2 solution	2476877

*Note: Crystal structures of (S,S)-ACPC$_6$ **2a** and (S,S)-ACPC$_8$ **3a** were newly analyzed since the known crystal structure of ACPC$_6$ and ACPC$_8$ were based on the (R,R)-enantiomer. No significant structural deviations were observed, aside from the handedness of the helix, which is determined by the opposite chirality of the ACPC monomer. Single crystals of rac-**8**, rac-**9**, and rac-**13** suitable for X-ray crystallography were grown by utilizing the racemic crystallography technique, from a 1:1 mixture of **8** with ent-**8**, **9** with ent-**9**, and **13** with ent-**13**, respectively.*

5.2. Crystallographic Information

Compound	**S8**	**S10**	**1b**	**1c**	**1d**	**1e**	**2a**	**2b**$_{\alpha}$	**2b**$_{\beta}$
Identification code (CCDC)	2447589	2447572	2004043	1967170	2002611	1975177	2129890	2021289	2041888
Empirical formula	$C_{24}H_{34}N_2O_4S$	C17H28N_2O_4S	$C_{36}H_{52}N_4O_6S$	$C_{36}H_{52}N_4O_4S_3$	$C_{36}H_{52}N_4O_6S$	$C_{36}H_{52}N_4O_4S_2$	$C_{48}H_{70}N_6O_9$	$C_{48}H_{70}N_6O_6S_3$	$C_{49}H_{71}Cl_3N_6O_6S_3$
Formula weight	446.59	356.47	668.87	700.99	668.87	684.93	875.10	923.28	1042.64
Temperature (K)	100	100	100.0	293(2)	293(2)	100.0	123	100.0	100.0
Crystal system	Monoclinic	Orthorhombic	Orthorhombic	Orthorhombic	Trigonal	Monoclinic	Triclinic	Triclinic	Orthorhombic
Space group	$P2_1$	$P2_12_12_1$	$P2_12_12_1$	$P2_12_12_1$	$P3_2$	$P2_1$	$P1$	$P1$	$P2_12_12_1$
a (Å)	5.1670(10)	10.183(2)	10.339(2)	9.1680(18)	10.5601(15)	12.572(3)	8.7845(18)	10.014(2)	11.369(2)
b (Å)	20.496(4)	11.467(2)	16.776(3)	20.033(4)	10.5601(15)	17.979(4)	11.580(2)	11.338(2)	18.996(4)
c (Å)	11.435(2)	15.889(3)	20.237(4)	20.387(4)	28.584(6)	24.677(5)	12.975(3)	13.992(3)	24.197(5)
α (˚)	90	90	90	90	90	90	69.83(3)	88.00(3)	90
β (˚)	100.32(3)	90	90	90	90	103.73(3)	82.36(3)	73.92(3)	90
γ (˚)	90	90	90	90	120	90	89.35(3)	63.96(3)	90
Volume (Å^3)	1191.4(4)	1855.3(6)	3510.0(12)	3744.3(13)	2760.5(10)	5418(2)	1227.1(5)	1364.6(6)	5225.7(18)
Z	2	4	4	4	3	6	1	1	4
ρ_{calc} (g/cm^3)	1.245	1.276	1.266	1.244	1.207	1.259	1.184	1.124	1.325
μ (mm^{-1})	0.162	0.190	0.128	0.232	0.101	0.187	0.082	0.177	0.335
F(000)	480.0	768.0	1440	1504.0	1080	2208.0	472	496.0	2216.0
Radiation (Å)	synchrotron (λ = 0.700)	synchrotron (λ = 0.700)	synchrotron (λ = 0.67748)	synchrotron (λ = 0.700)	synchrotron (λ = 0.630)	synchrotron (λ = 0.69999)	0.71073 Å	synchrotron (λ = 0.700)	synchrotron (λ = 0.700)
2Θ range for data collection (°)	4.068–49.998	4.68–59.492	3.006–55.95	2.808–59.672	4.144–67.03	1.674–49	5.964–50.000	2.998–59.998	2.684–59.998
Index ranges	−6 ≤ h ≤ 6<br>−23 ≤ k ≤ 23<br>−13 ≤ l ≤ 13	−13 ≤ h ≤ 13<br>−16 ≤ k ≤ 16<br>−22 ≤ l ≤ 22	−12 ≤ h ≤ 12<br>−18 ≤ k ≤ 18<br>−24 ≤ l ≤ 24	−13 ≤ h ≤ 13<br>−28 ≤ k ≤ 28<br>−28 ≤ l ≤ 28	−16 ≤ h ≤ 16<br>−18 ≤ k ≤ 18<br>−47 ≤ l ≤ 47	−14 ≤ h ≤ 14<br>−21 ≤ k ≤ 21<br>−29 ≤ l ≤ 29	−9 ≤ h ≤ 10<br>−13 ≤ k ≤ 13<br>−15 ≤ l ≤ 15	−14 ≤ h ≤ 14<br>−16 ≤ k ≤ 16<br>−19 ≤ l ≤ 19	−16 ≤ h ≤ 16<br>−27 ≤ k ≤ 27<br>−34 ≤ l ≤34
Reflections collected	8059	19598	23836	38944	35513	31203	9786	76418	54599
Independent reflections	4215<br>R_{int} = 0.0482,<br>R_{sigma} = 0.1243	5399<br>R_{int} = 0.0426,<br>R_{sigma} = 0.0358	7381<br>R_{int} = 0.0846,<br>R_{sigma} = 0.0731	11231<br>R_{int} = 0.0962,<br>R_{sigma} = 0.0627	17371<br>R_{int} = 0.0681,<br>R_{sigma} = 0.0631	18348<br>R_{int} = 0.1665,<br>R_{sigma} = 0.1670	7273<br>R_{int} = 0.1056<br>R_{sigma} = 0.1630	16120<br>R_{int} = 0.0826,<br>R_{sigma} = 0.0554	15940<br>R_{int} = 0.1190,<br>R_{sigma} = 0.0872
Goodness-of-fit on F^2	1.061	1.123	1.033	1.043	1.127	1.045	1.033	1.003	1.084
Final R indexes [I>=2σ (I)]	R_1 = 0.0482,<br>wR_2 = 0.1243	R_1 = 0.0383,<br>wR_2 = 0.0959	R_1 = 0.0561,<br>wR_2 = 0.1448	R_1 = 0.0394,<br>wR_2 = 0.1025	R_1 = 0.0604,<br>wR_2 = 0.1761	R_1 = 0.1141,<br>wR_2 = 0.2853	R_1 = 0.1021,<br>wR_2 = 0.2460	R_1 = 0.0604,<br>wR_2 = 0.1660	R_1 = 0.0624,<br>wR_2 = 0.1629
Final R indexes [all data]	R_1 = 0.0509,<br>wR_2 = 0.1264	R_1 = 0.0398,<br>wR_2 = 0.0974	R_1 = 0.0743,<br>wR_2 = 0.1587	R_1 = 0.0405,<br>wR_2 = 0.1034	R_1 = 0.0719,<br>wR_2 = 0.1864	R_1 = 0.1421,<br>wR_2 = 0.3296	R_1 = 0.2033,<br>wR_2 = 0.3155	R_1 = 0.0877,<br>wR_2 = 0.1841	R_1 = 0.0746,<br>wR_2 = 0.1704
Largest diff. peak/hole (e·Å^{-3})	0.53/−0.47	0.39/−0.27	0.33/−0.51	0.65/−0.34	0.52/−0.45	1.53/−0.74	0.281/−0.258	0.46/−0.47	0.78/−0.79
Flack parameter	0.08(5)	−0.02(2)	0.06(5)	−0.019(16)	−0.05(4)	0.02(7)	–	0.052(18)	0.03(2)

*Note: For **2a**, the Flack check could not be completed due to an insufficient number of Bijvoet pairs in the dataset, resulting from very weak diffraction of the crystal.*

Compound	**3a**	**3b**	**3c**	**5a**	**5b**	**5c**	*rac*-**8**	*rac*-**9**	*rac*-**13**
Identification code (CCDC)	2129889	2048422	2048513	2350730	2435172	2447552	2213750	2256299	2476877
Empirical formula	$C_{60}H_{88}N_8O_{11}$	$C_{62.5}H_{94}N_8O_7S_4$	$C_{62}H_{92}N_8O_6S_6$	$C_{24}H_{36}N_4O_2S_2$	$C_{38}H_{56}Cl_6N_6O_3S_3$	$C_{49}H_{73}Cl_3N_8O_4S_4$	$C_{84}H_{124}N_{12}O_9S_6$	$C_{108}H_{160}N_{16}O_{11}S_8$	$C_{87}H_{136}N_{12}O_{18}$
Formula weight	1097.39	1197.69	1237.79	476.69	953.73	1072.74	1638.30	2114.99	1638.07
Temperature (K)	173	100.0	100.0	100.0	100.0	100.00	105.00	100.00	100.00
Crystal system	Orthorhombic	Monoclinic	Monoclinic	Orthorhombic	Monoclinic	Monoclinic	Monoclinic	Monoclinic	Triclinic
Space group	$P2_12_12$	$P2_1$	$P2_1$	$P2_12_12$	$P2_1$	$P2_1$	$P2_1/c$	$P2_1/c$	$P{-}1$
a (Å)	20.350(4)	11.112(2)	11.734(2)	12.225(2)	15.328(3)	9.1600(18)	13.925(3)	13.659(3)	9.818(2)
b (Å)	30.195(6)	22.557(5)	19.654(4)	12.375(2)	18.521(4)	17.641(4)	13.817(3)	13.943(3)	21.698(4)
c (Å)	10.106(2)	28.385(6)	28.128(6)	7.5530(15)	16.192(3)	32.686(7)	50.538(10)	66.950(13)	23.182(5)
α (˚)	90	90	90	90	90	90	90	90	115.89(3)
β (˚)	90	90.07(3)	90.57(3)	90	90.03(3)	90.14(3)	97.72(3)	92.20(3)	97.68(3)
γ (˚)	90	90	90	90	90	90	90	90	91.85(3)
Volume ($Å^3$)	6210(2)	7115(2)	6487(2)	1142.7(4)	4596.7(16)	5281.8(18)	9635(3)	12741(4)	4380.3(18)
Z	4	4	4	2	4	4	4	4	2
ρ_{calc} (g/cm^3)	1.174	1.118	1.267	1.385	1.378	1.349	1.129	1.103	1.242
μ (mm^{-1})	0.081	0.178	0.256	0.254	0.531	0.369	0.191	0.190	0.084
F(000)	2368	2580.0	2656.0	512.0	2000.0	2280.0	3520.0	4544.0	1772.0
Radiation (Å)	0.71073 Å	synchrotron (λ = 0.700)	synchrotron (λ = 0.700)	synchrotron (λ = 0.700)	synchrotron (λ = 0.700)	synchrotron (λ = 0.700)	synchrotron (λ = 0.700)	synchrotron (λ = 0.700)	synchrotron (λ = 0.700)
2Θ range for data collection (°)	6.054–50.052	1.414–50	1.426–60	4.612–59.576	2.478–48.498	2.454–48.5	1.602–57	2.398–48	3.414–51.998
Index ranges	−24 ≤ h ≤ 24<br>−35 ≤ k ≤ 35<br>−12 ≤ l ≤ 11	−13 ≤ h ≤ 13<br>−27 ≤ k ≤ 27<br>−34 ≤ l ≤ 34	−16 ≤ h ≤ 16<br>−27 ≤ k ≤ 27<br>−37 ≤ l ≤ 38	−17 ≤ h ≤ 17<br>−17 ≤ k ≤ 17<br>−10 ≤ l ≤ 10	−17 ≤ h ≤ 17<br>−21 ≤ k ≤ 21<br>−19 ≤ l ≤ 19	−10 ≤ h ≤ 10<br>−20 ≤ k ≤ 20<br>−38 ≤ l ≤ 38	−18 ≤ h ≤ 18<br>−18 ≤ k ≤ 18<br>−65 ≤ l ≤ 65	−15 ≤ h ≤ 15<br>−16 ≤ k ≤ 16<br>−75 ≤ l ≤ 75	−12 ≤ h ≤ 12<br>−27 ≤ k ≤ 27<br>−29 ≤ l ≤ 29
Reflections collected	49407	44696	61428	11756	98158	32458	82719	70973	35704
Independent reflections	10929<br>R_{int} = 0.1106<br>R_{sigma} = 0.1315	25059<br>R_{int} = 0.0879,<br>R_{sigma} = 0.1162	36192<br>R_{int} = 0.1252,<br>R_{sigma} = 0.1381	3420<br>R_{int} = 0.1094,<br>R_{sigma} = 0.0752	15287<br>R_{int} = 0.1782,<br>R_{sigma} = 0.0788	17844<br>R_{int} = 0.1546,<br>R_{sigma} = 0.1927	24391<br>R_{int} = 0.0828,<br>R_{sigma} = 0.0553	20778<br>R_{int} = 0.1166,<br>R_{sigma} = 0.1222	17948<br>R_{int} = 0.0288,<br>R_{sigma} = 0.0386
Goodness-of-fit on F^2	1.019	1.019	1.121	1.147	1.103	1.029	1.250	0.936	1.070
Final R indexes [I>=2σ (I)]	R_1 = 0.1108,<br>wR_2 = 0.2817	R_1 = 0.0710,<br>wR_2 = 0.1888	R_1 = 0.1023,<br>wR_2 = 0.2614	R_1 = 0.0640,<br>wR_2 = 0.1797	R_1 = 0.1225,<br>wR_2 = 0.2946	R_1 = 0.0908,<br>wR_2 = 0.2030	R_1 = 0.1031,<br>wR_2 = 0.3194	R_1 = 0.0921,<br>wR_2 = 0.2633	R_1 = 0.1137,<br>wR_2 = 0.3264
Final R indexes [all data]	R_1 = 0.1939,<br>wR_2 = 0.3395	R_1 = 0.0990,<br>wR_2 = 0.2118	R_1 = 0.1377,<br>wR_2 = 0.3140	R_1 = 0.0753,<br>wR_2 = 0.1949	R_1 = 0.1246,<br>wR_2 = 0.2974	R_1 = 0.1685,<br>wR_2 = 0.2457	R_1 = 0.1217,<br>wR_2 = 0.3454	R_1 = 0.1724,<br>wR_2 = 0.2972	R_1 = 0.1347,<br>wR_2 = 0.3460
Largest diff. peak/hole ($e{\cdot}Å^{-3}$)	0.390/−0.295	0.45/−0.50	0.84/−0.72	0.85/−0.89	0.63/−0.72	0.80/−0.43	0.94/−0.75	0.75/−0.58	0.67/−0.78
Flack parameter	−0.2(8)	−0.02(3)	−0.06(3)	0.04(4)	0.11(2)	0.14(8)	–	–	–

*Note: No Flack parameter is reported for rac-**8**, rac-**9**, and rac-**13**, due to the centrosymmetric space group $P2_1/c$ (for rac-**8** and rac-**9**) and P−1 (for rac-**13**).*

6. NMR Spectra of Newly Synthesized Compounds

6.1. 1D NMR Spectra

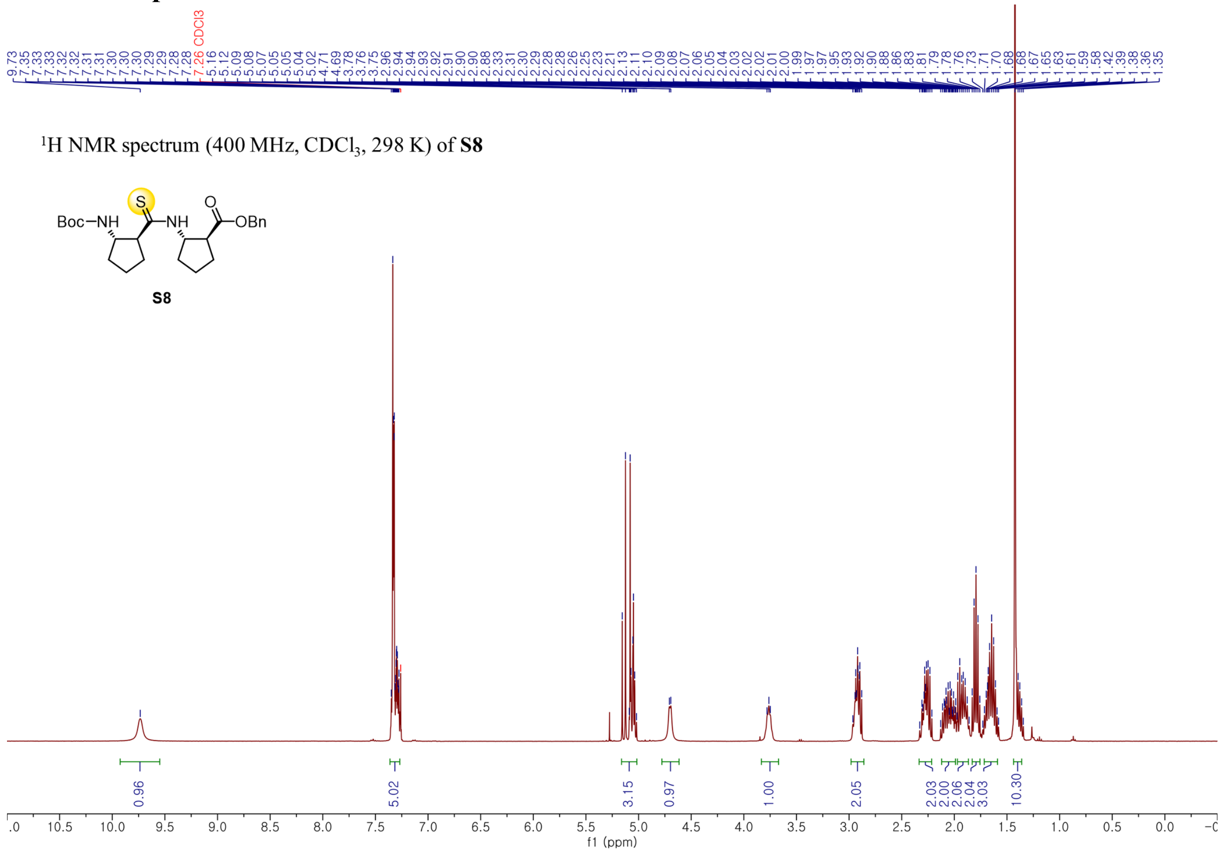

^{1}H NMR spectrum (400 MHz, $CDCl_3$, 298 K) of **S8**

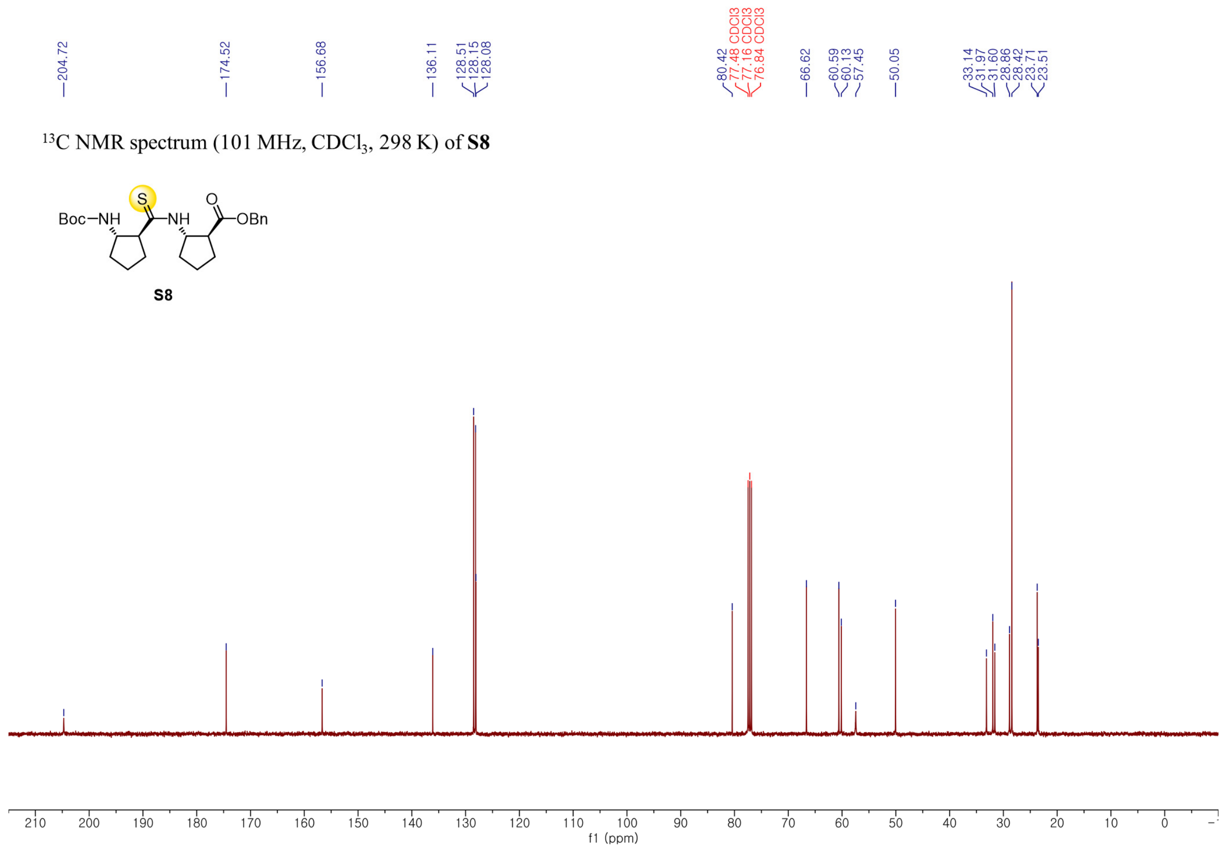

^{13}C NMR spectrum (101 MHz, $CDCl_3$, 298 K) of **S8**

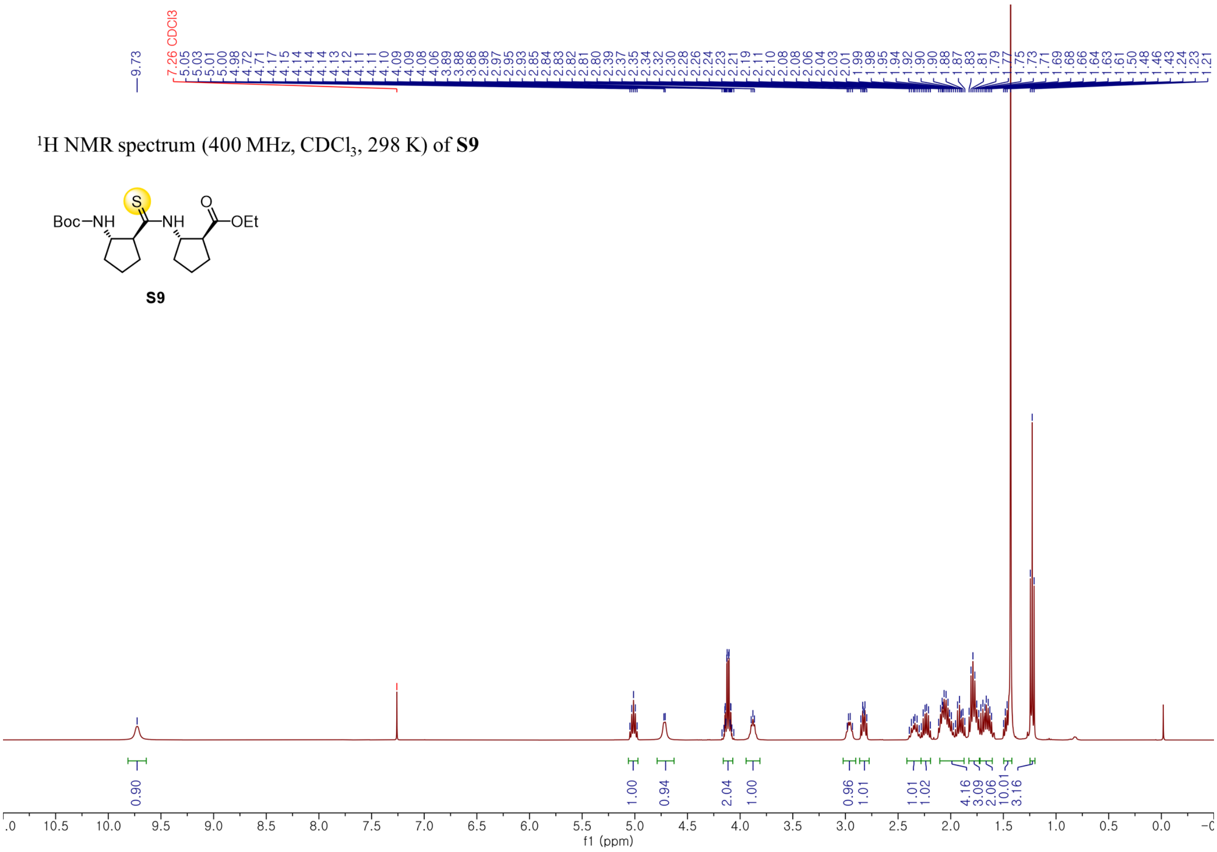

^{1}H NMR spectrum (400 MHz, $CDCl_3$, 298 K) of **S9**

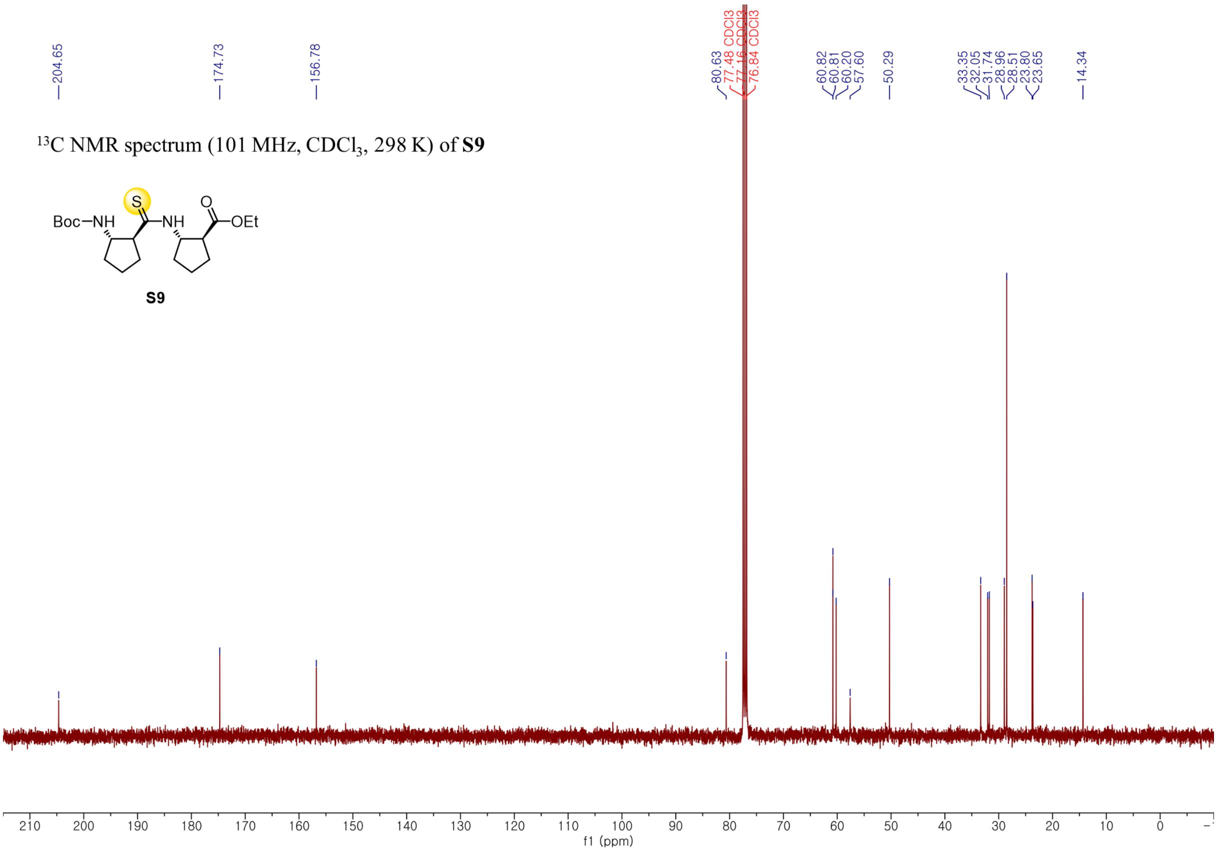

^{13}C NMR spectrum (101 MHz, $CDCl_3$, 298 K) of **S9**

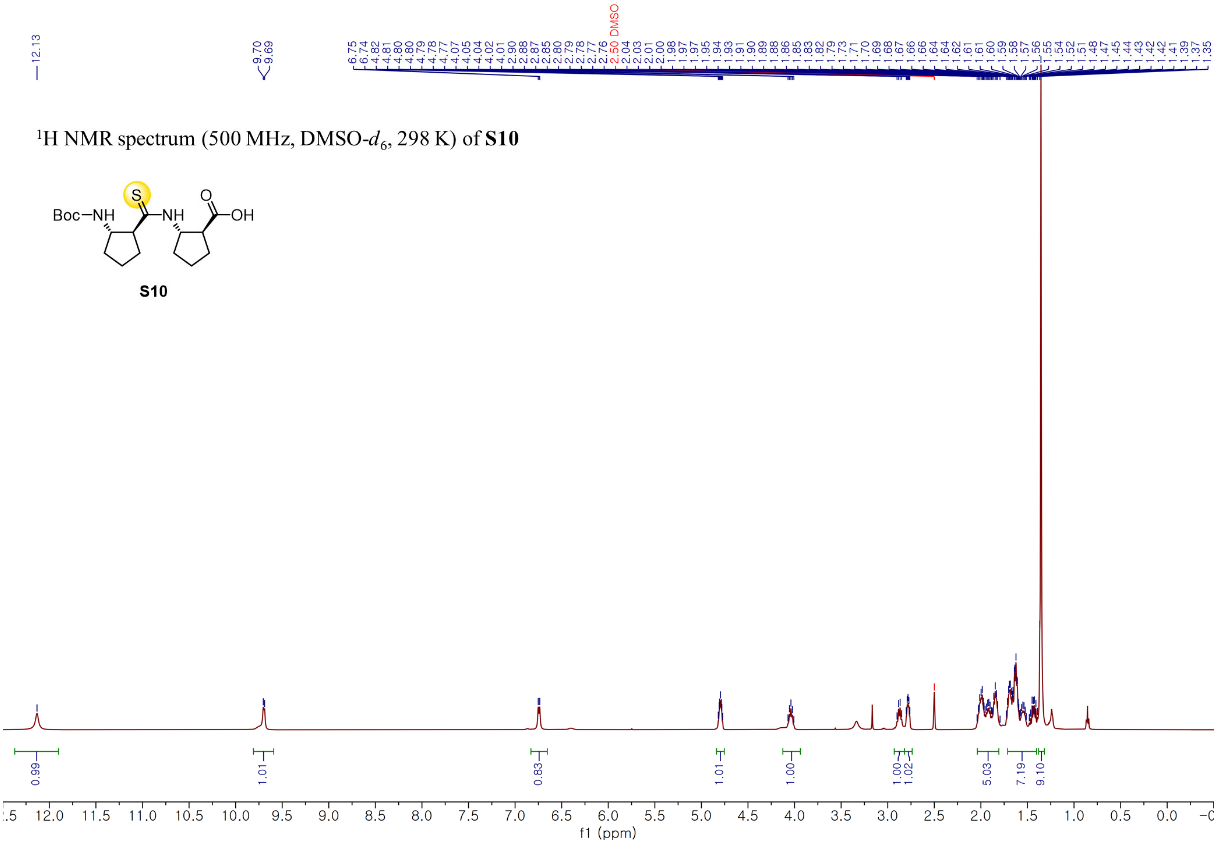

^{1}H NMR spectrum (500 MHz, DMSO-d_6, 298 K) of **S10**

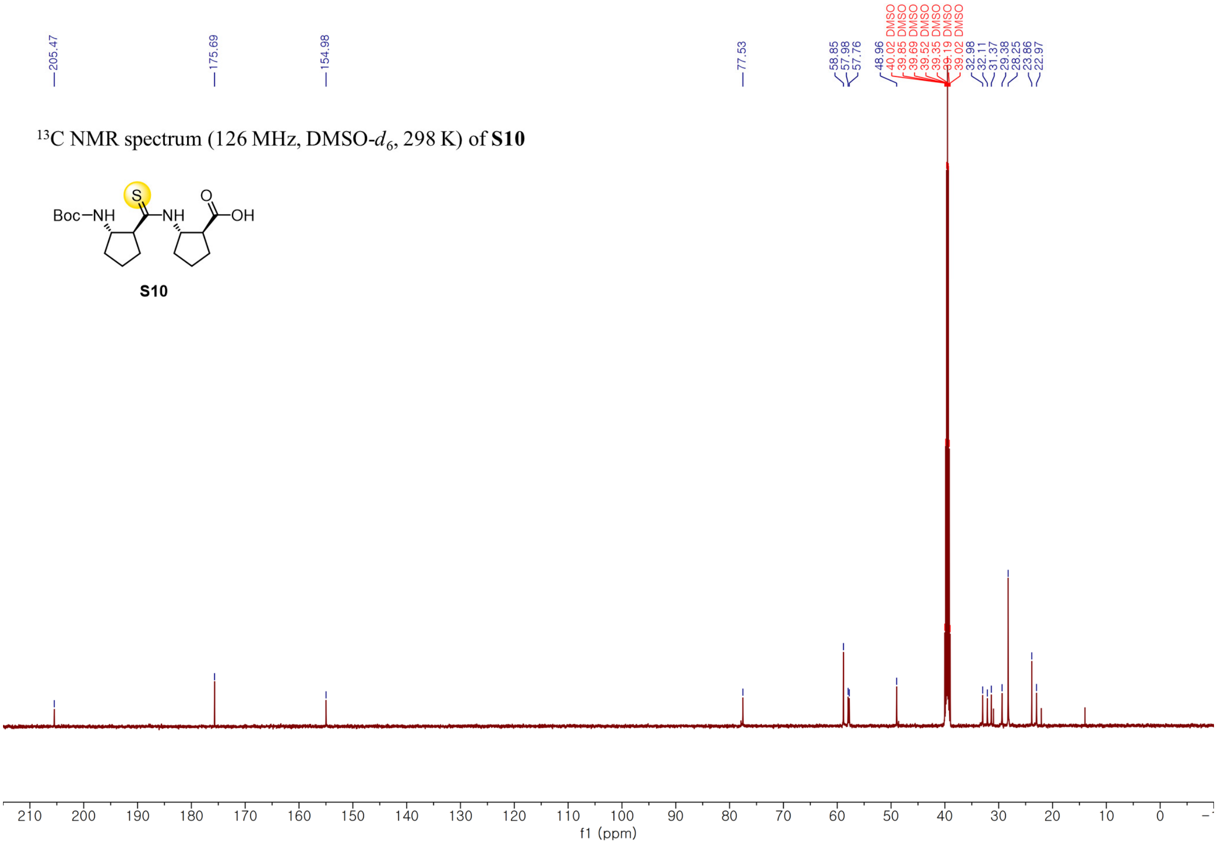

^{13}C NMR spectrum (126 MHz, DMSO-d_6, 298 K) of **S10**

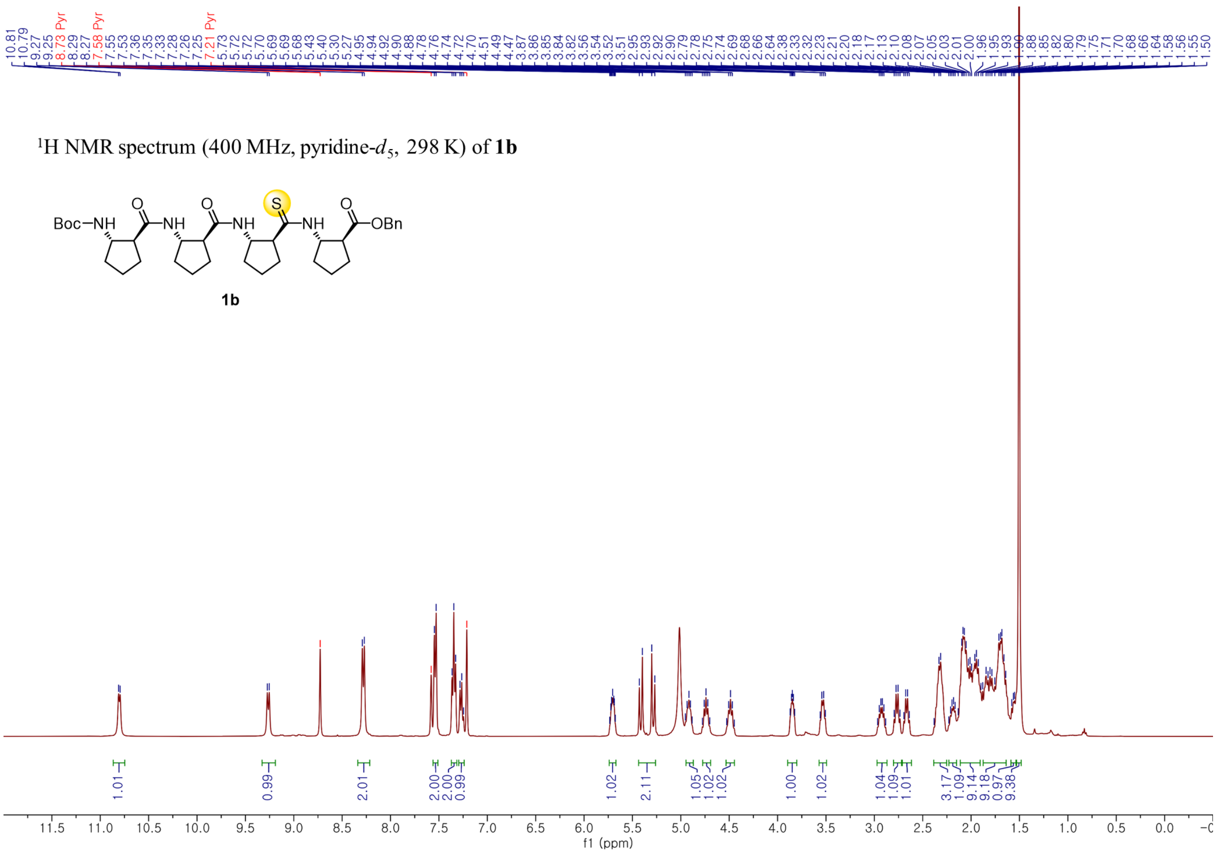

^{1}H NMR spectrum (400 MHz, pyridine-d_5, 298 K) of **1b**

1b

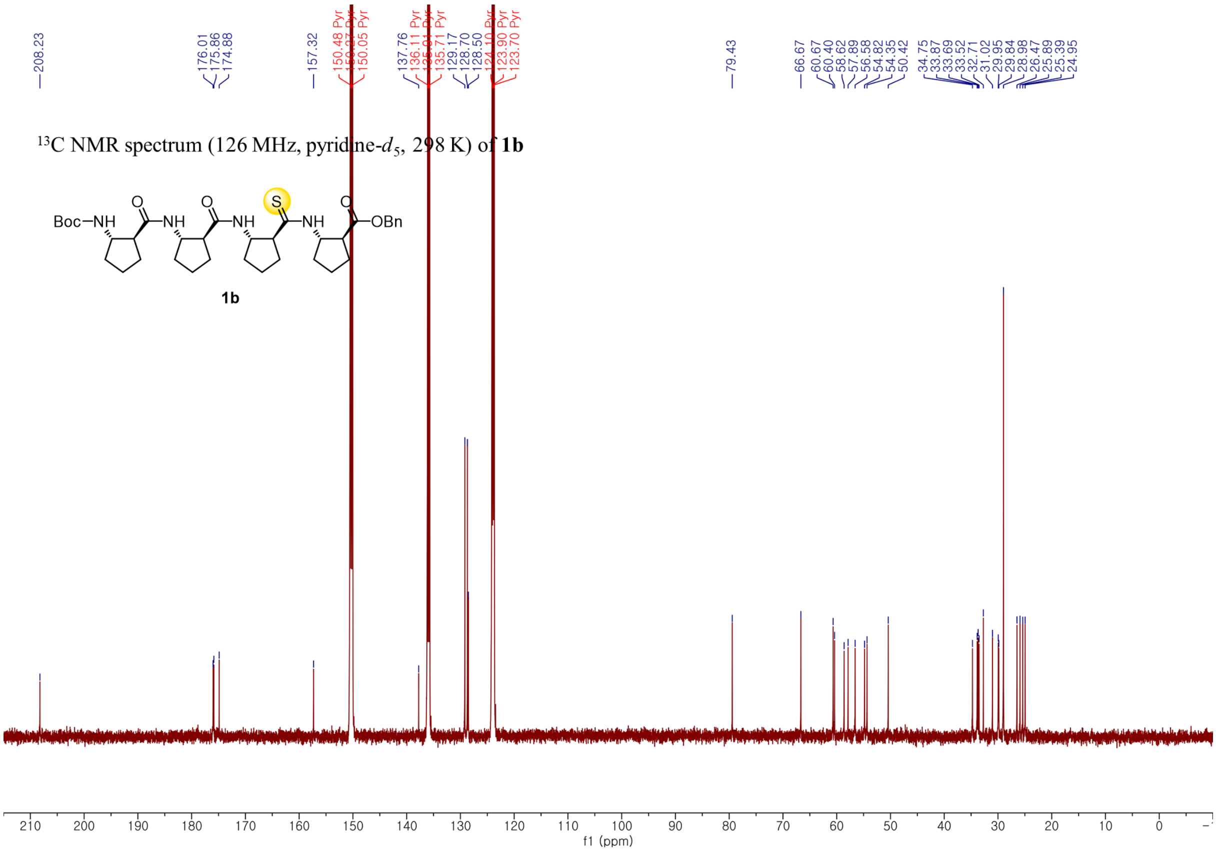

^{13}C NMR spectrum (126 MHz, pyridine-d_5, 298 K) of **1b**

1b

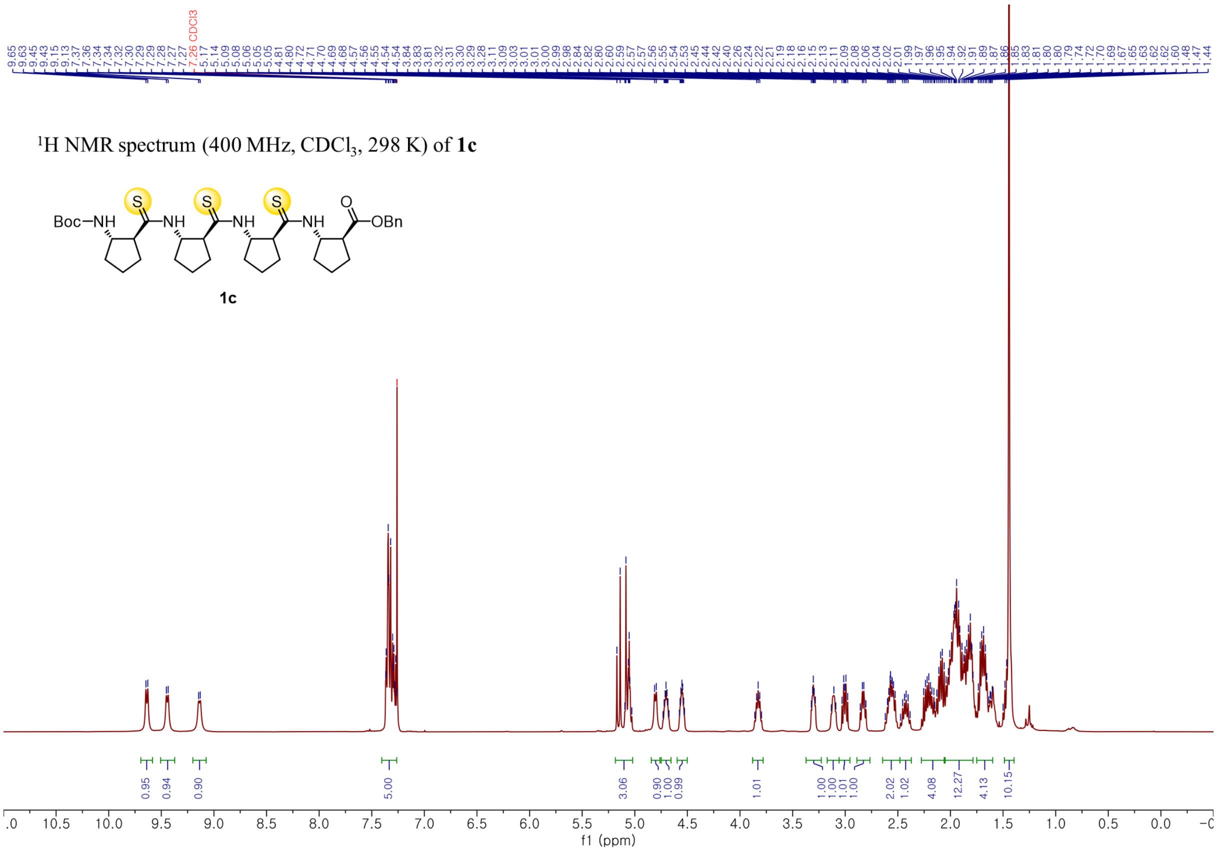

^{1}H NMR spectrum (400 MHz, $CDCl_3$, 298 K) of **1c**

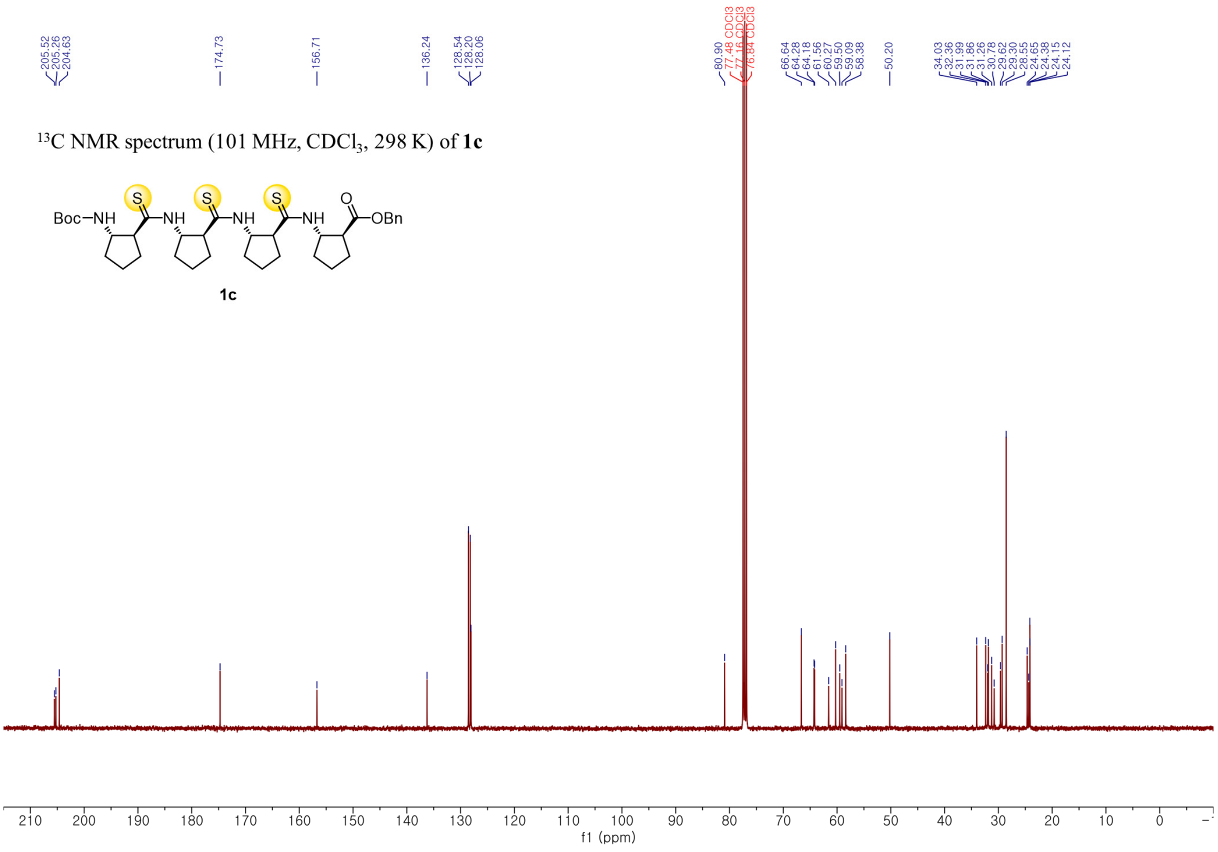

^{13}C NMR spectrum (101 MHz, $CDCl_3$, 298 K) of **1c**

^{1}H NMR spectrum (500 MHz, $CDCl_3$, 298 K) of **1d**

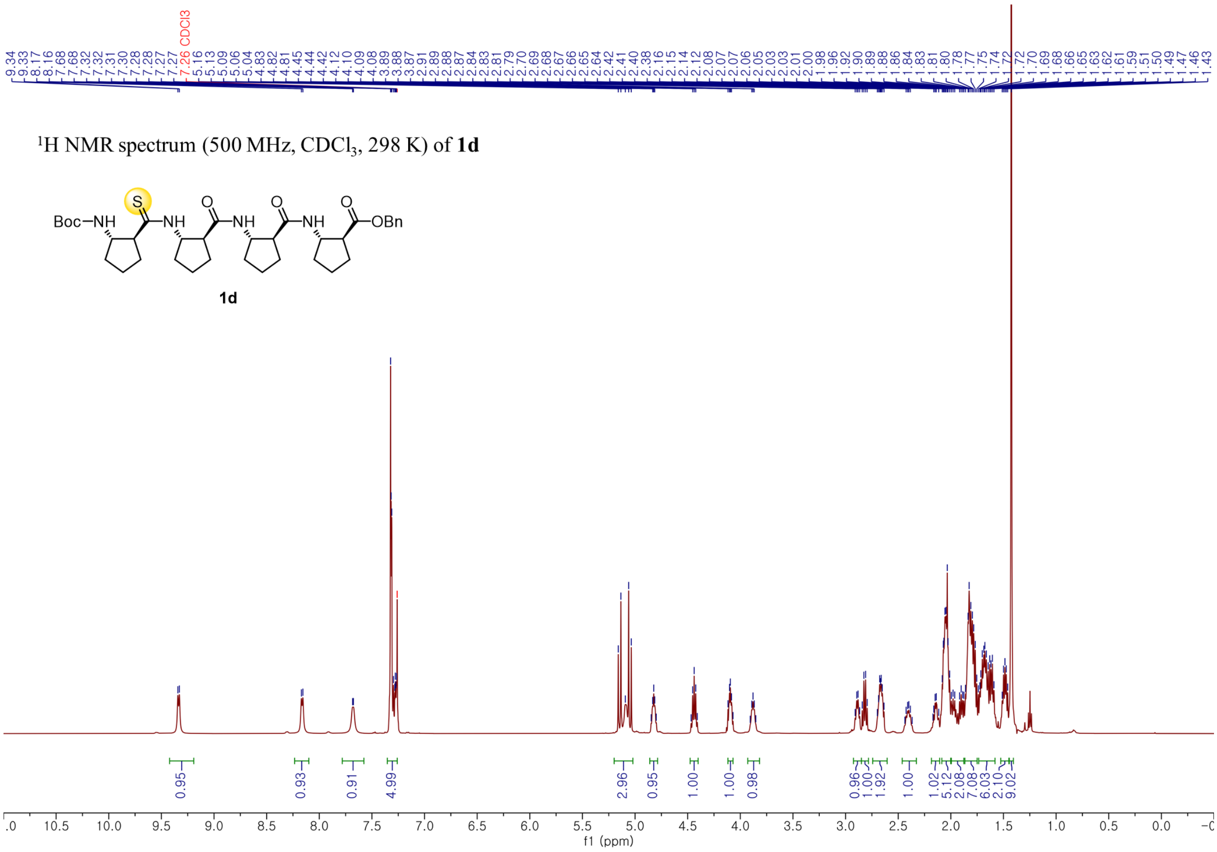

^{13}C NMR spectrum (126 MHz, $CDCl_3$, 298 K) of **1d**

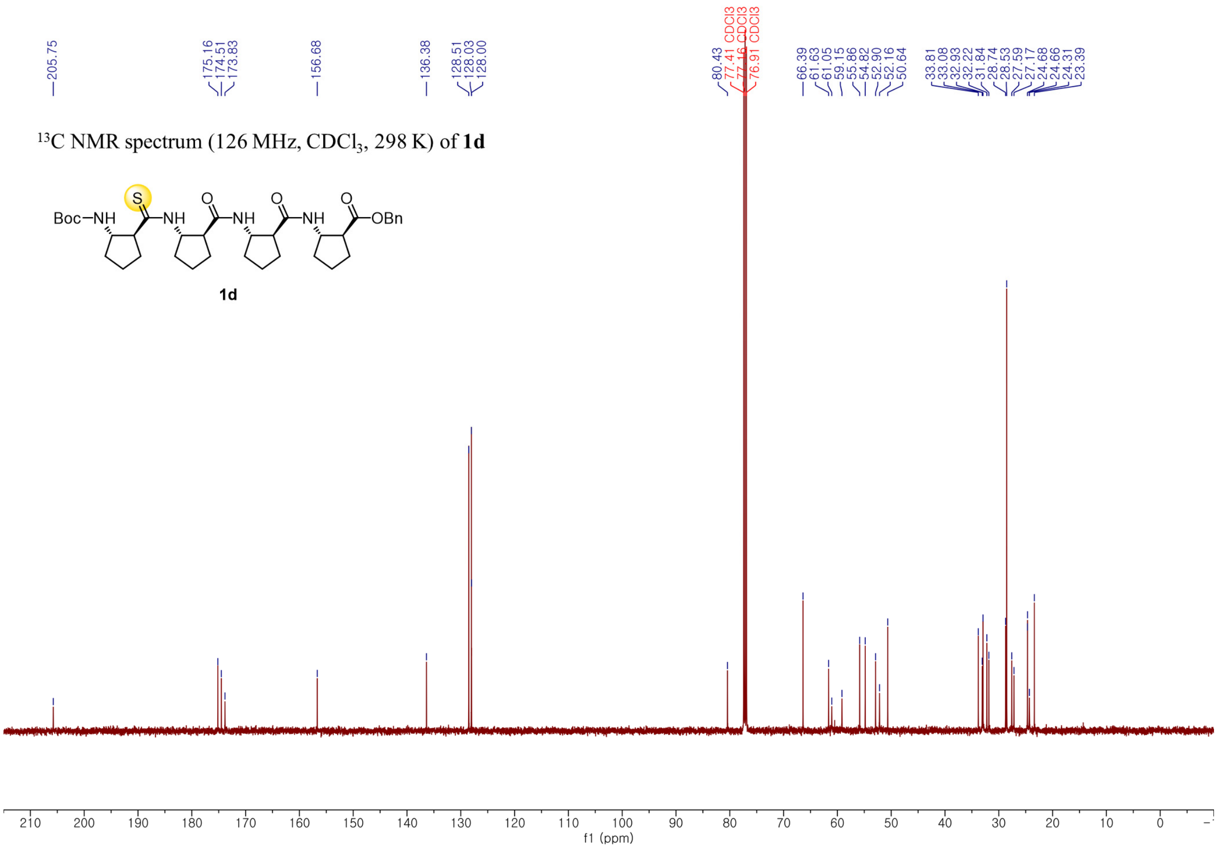

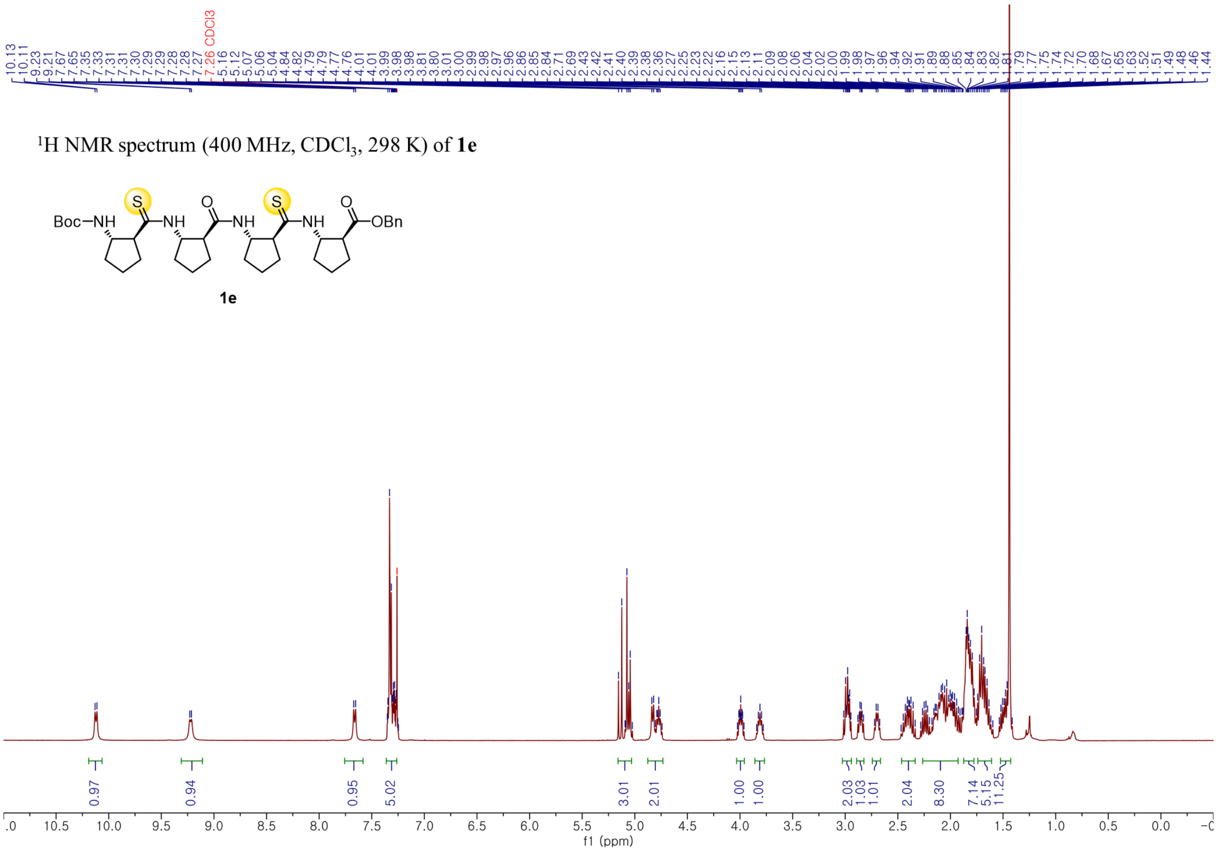

^{1}H NMR spectrum (400 MHz, $CDCl_3$, 298 K) of **1e**

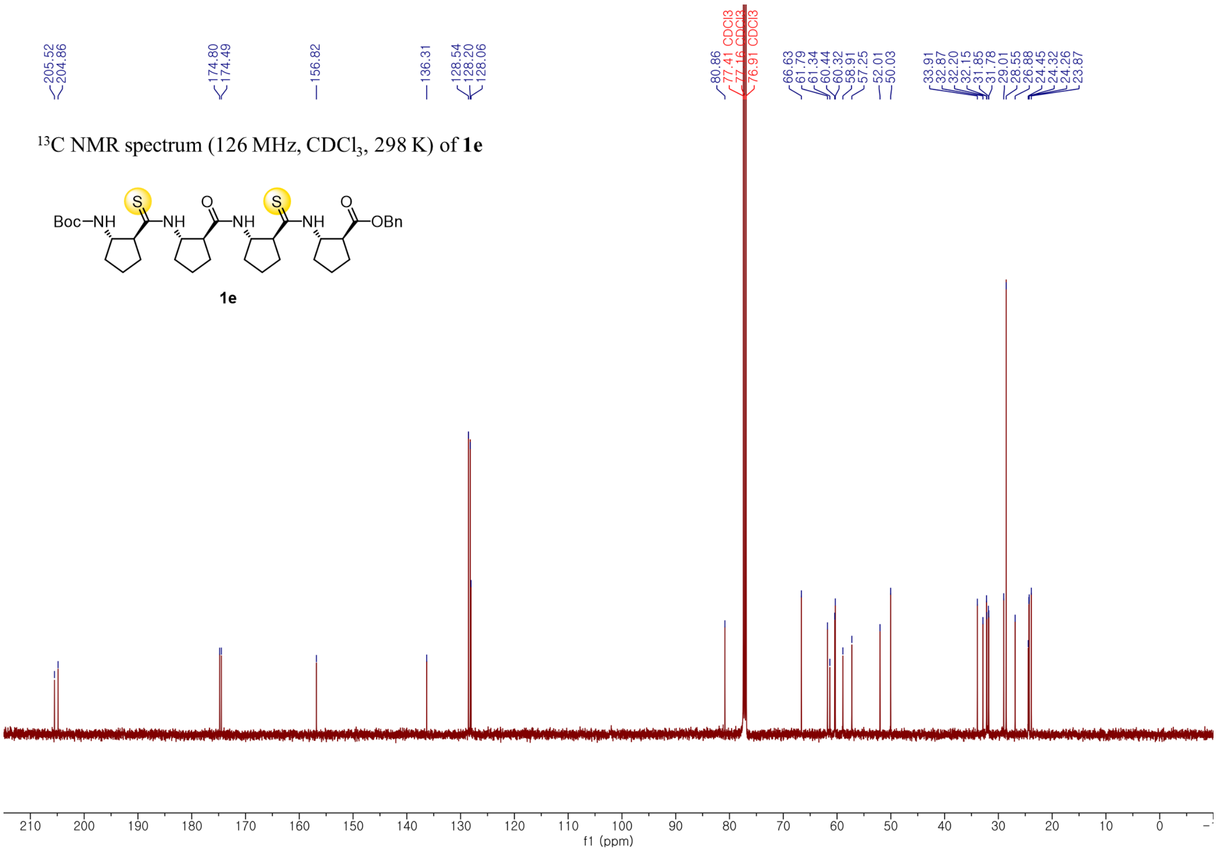

^{13}C NMR spectrum (126 MHz, $CDCl_3$, 298 K) of **1e**

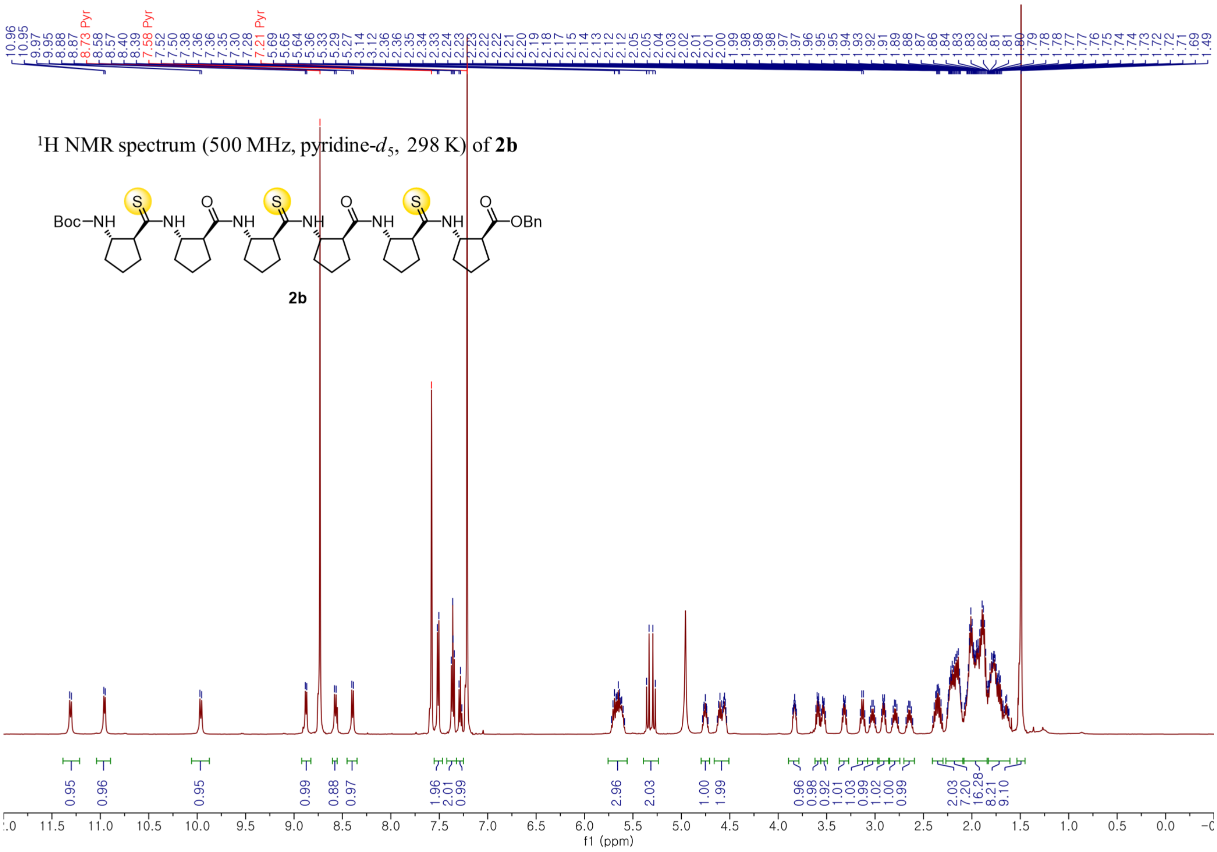
¹H NMR spectrum (500 MHz, pyridine-d_5, 298 K) of **2b**
Boc–NH
NH
OBn
2b
f1 (ppm)

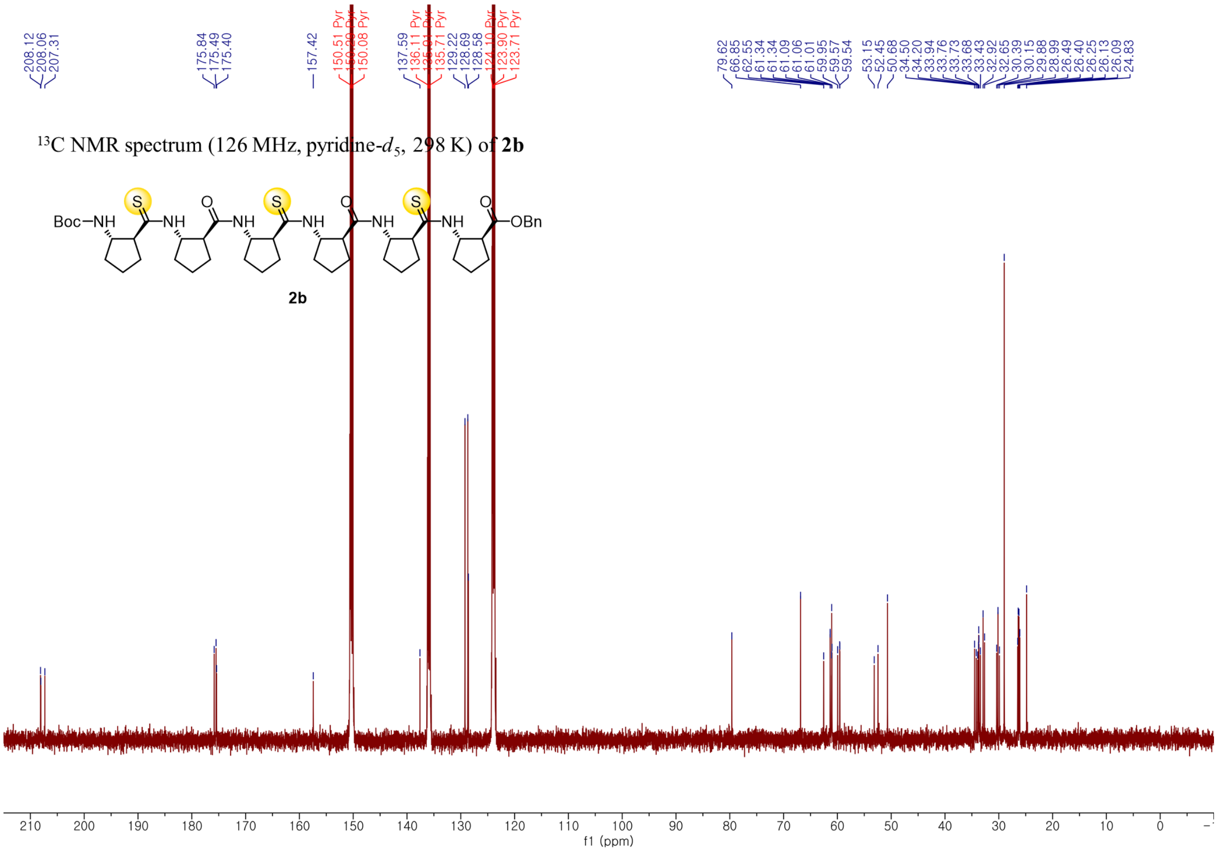
¹³C NMR spectrum (126 MHz, pyridine-d_5, 298 K) of **2b**
Boc–NH
NH
OBn
2b
f1 (ppm)

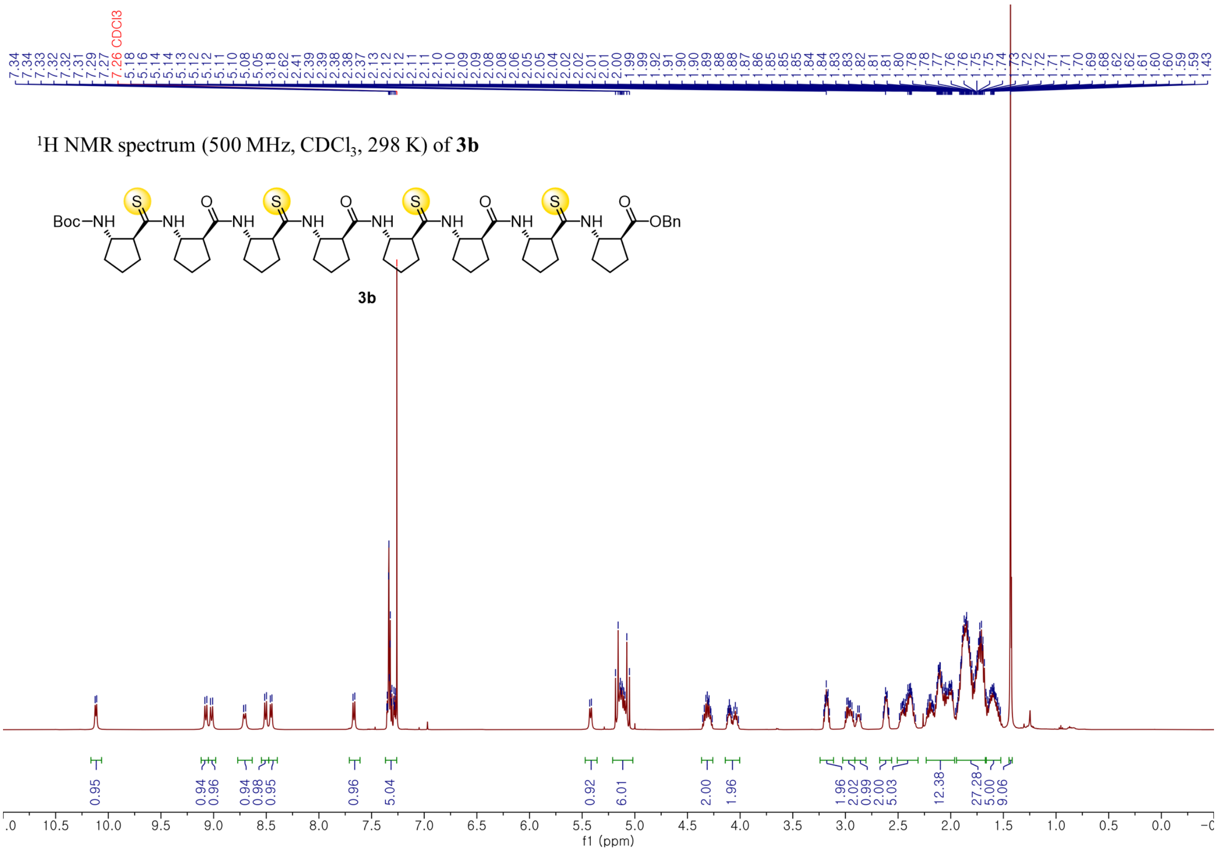

^{1}H NMR spectrum (500 MHz, $CDCl_3$, 298 K) of **3b**

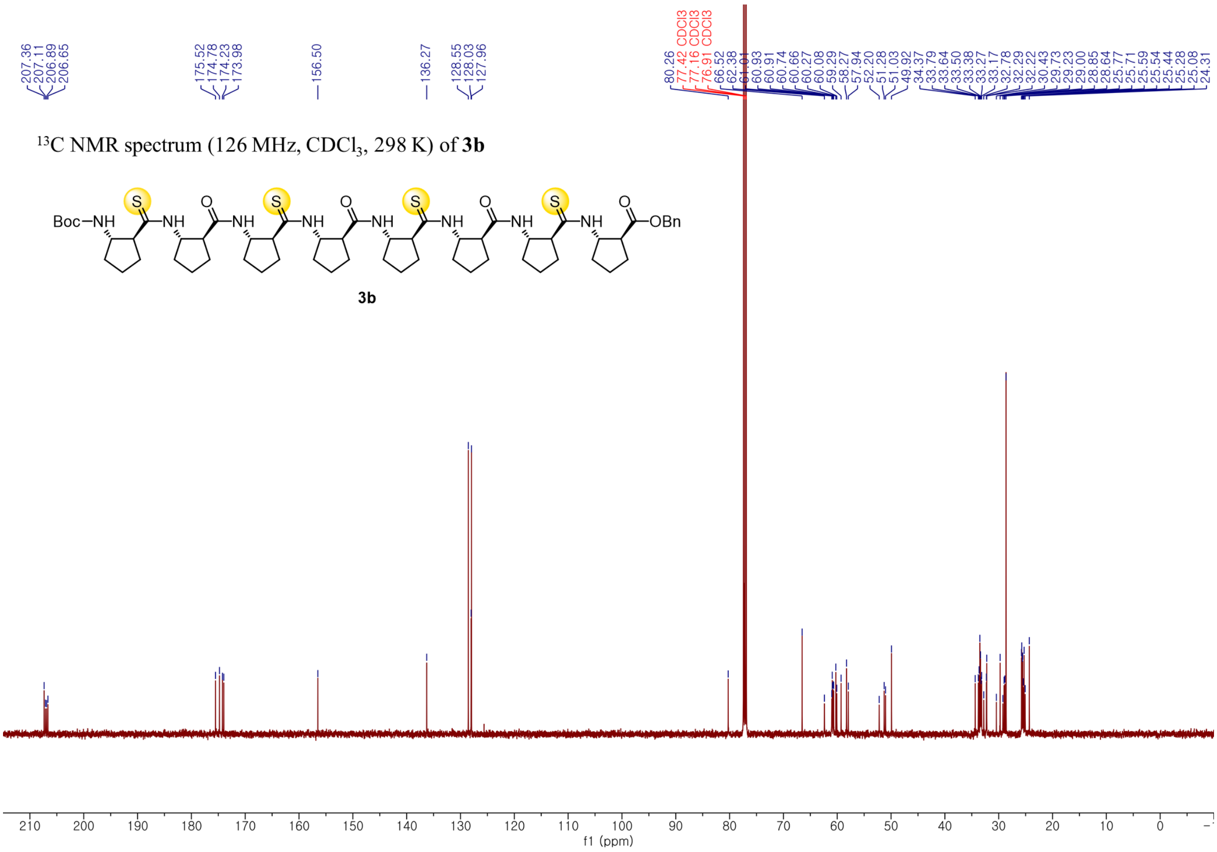

^{13}C NMR spectrum (126 MHz, $CDCl_3$, 298 K) of **3b**

^{1}H NMR spectrum (500 MHz, $CDCl_3$, 298 K) of **S11**

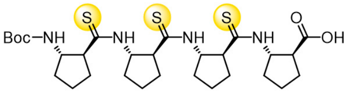

S11

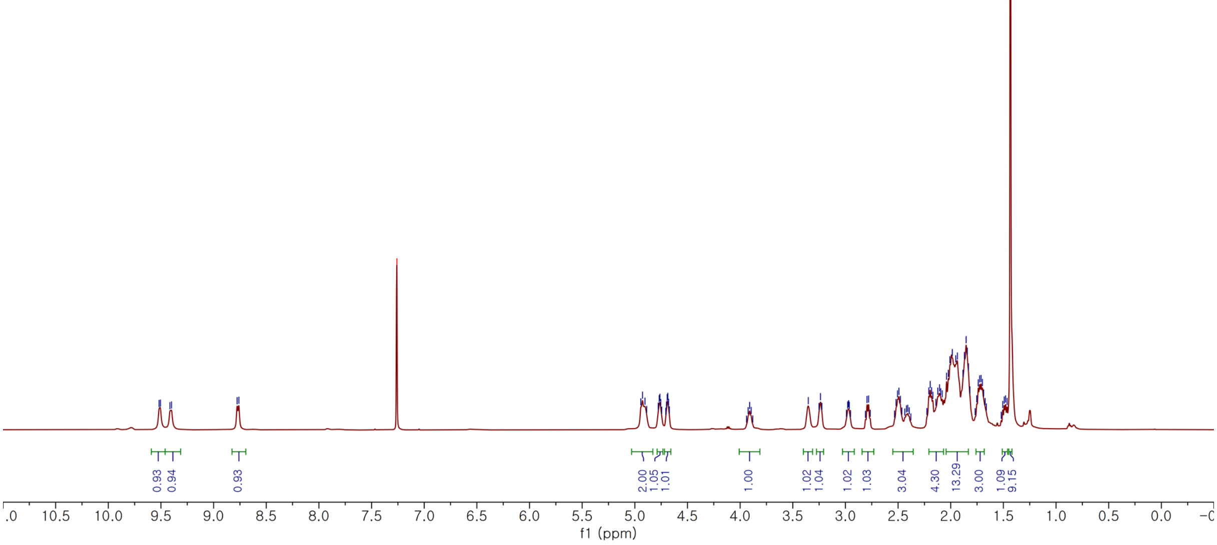

^{13}C NMR spectrum (126 MHz, $CDCl_3$, 298 K) of **S11**

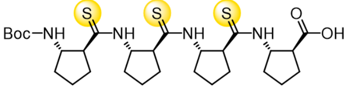

S11

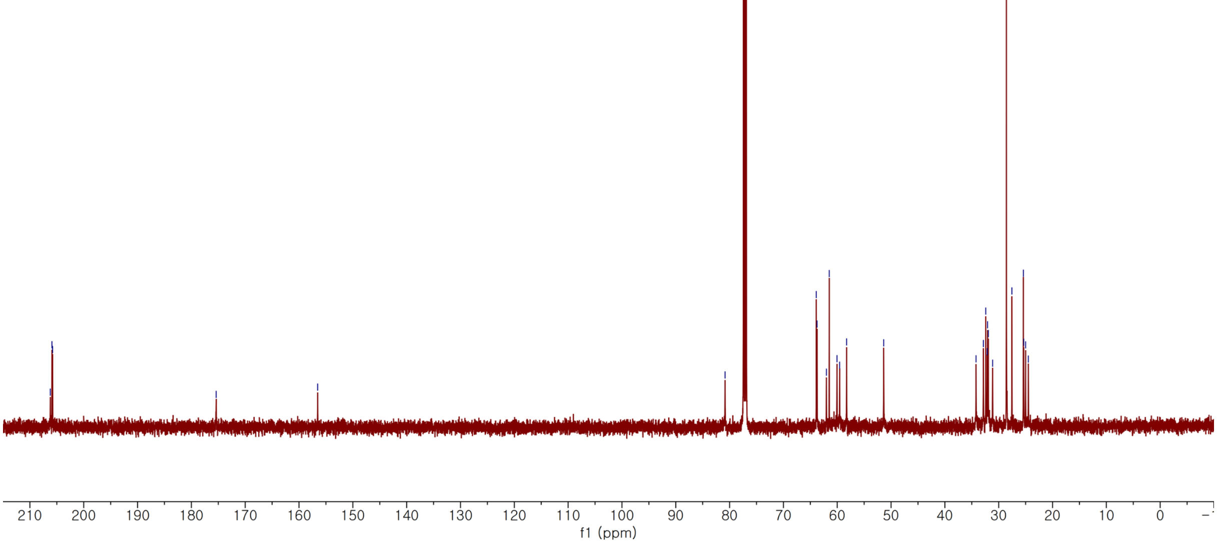

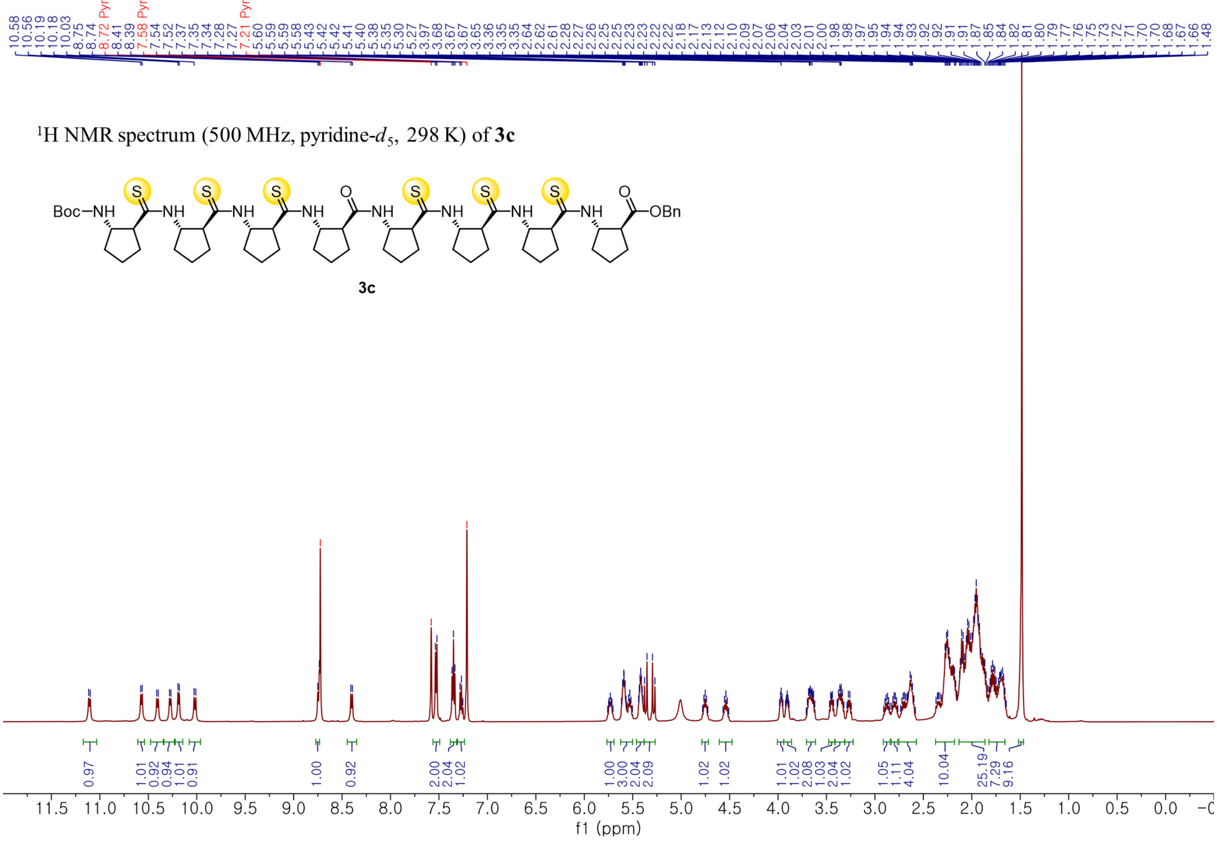

^{1}H NMR spectrum (500 MHz, pyridine-d_5, 298 K) of **3c**

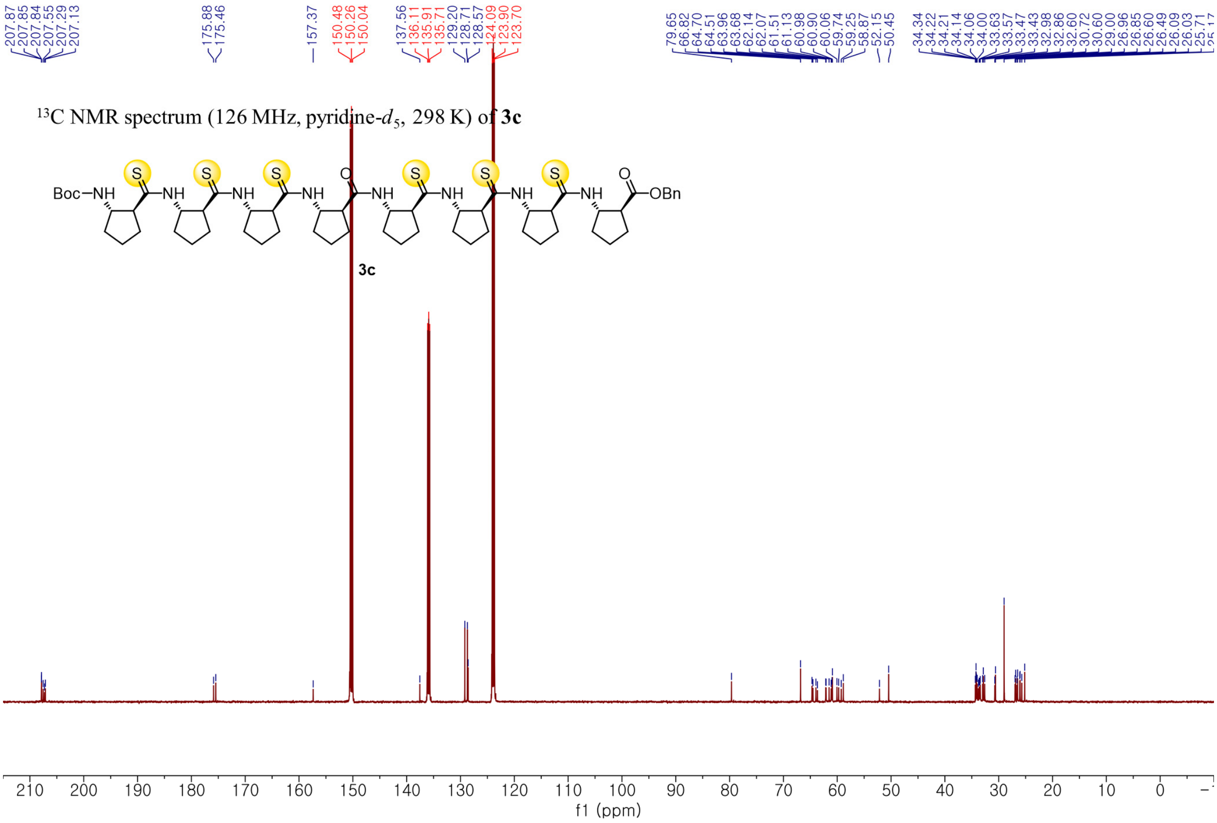

^{13}C NMR spectrum (126 MHz, pyridine-d_5, 298 K) of **3c**

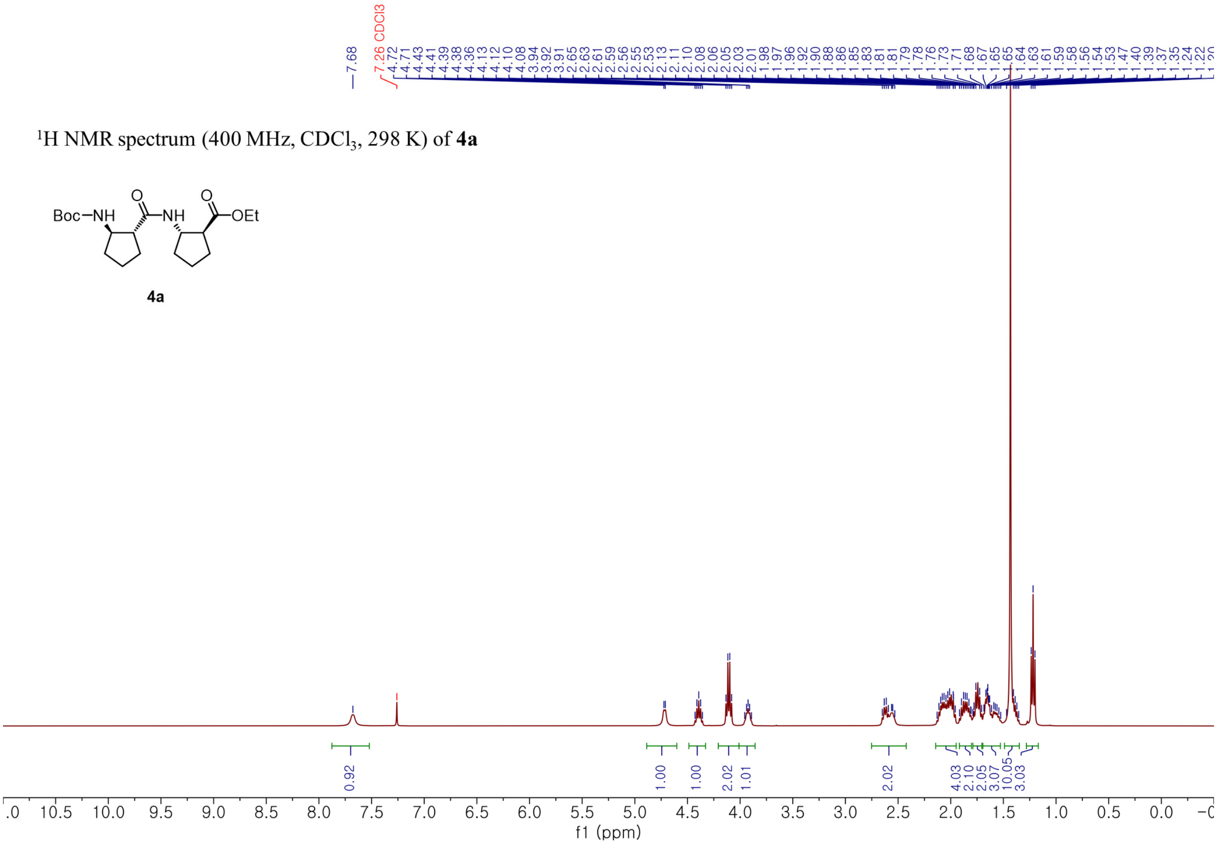

^{1}H NMR spectrum (400 MHz, $CDCl_3$, 298 K) of **4a**

4a

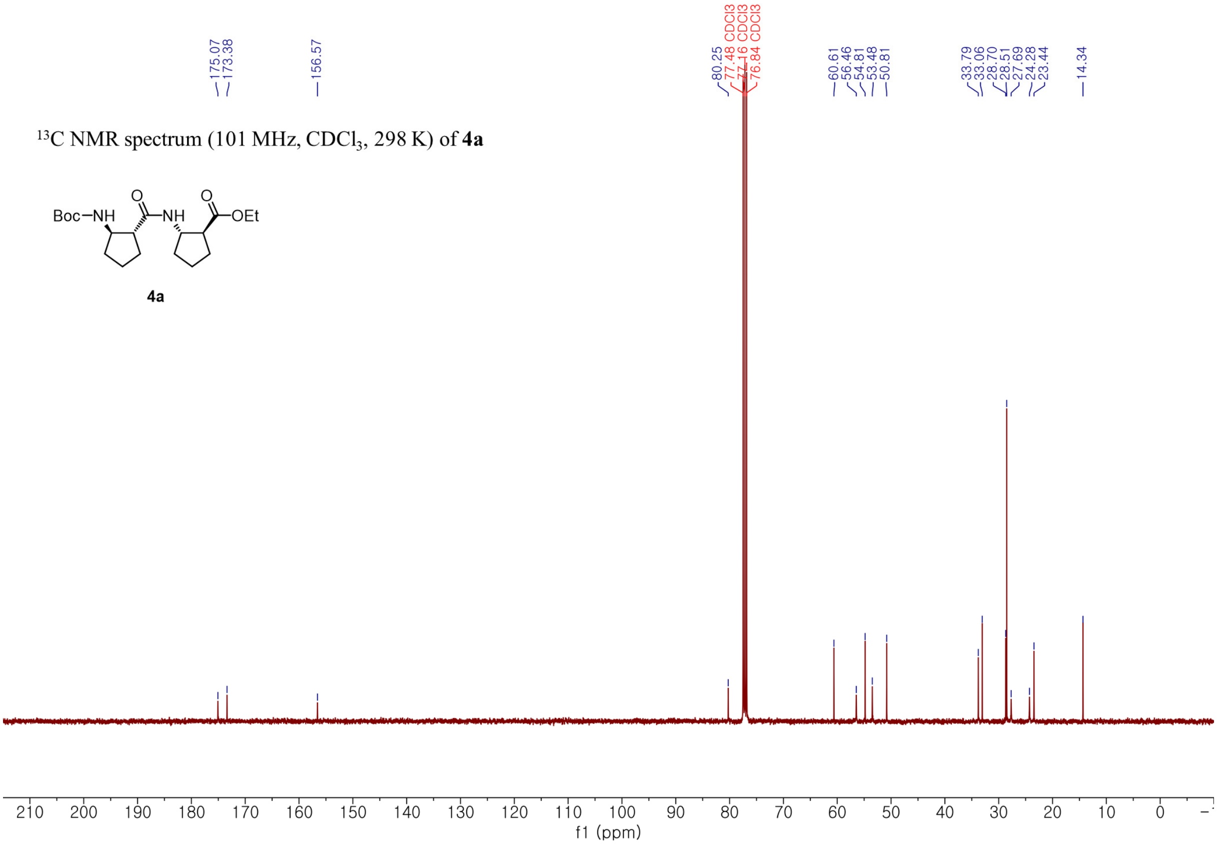

^{13}C NMR spectrum (101 MHz, $CDCl_3$, 298 K) of **4a**

4a

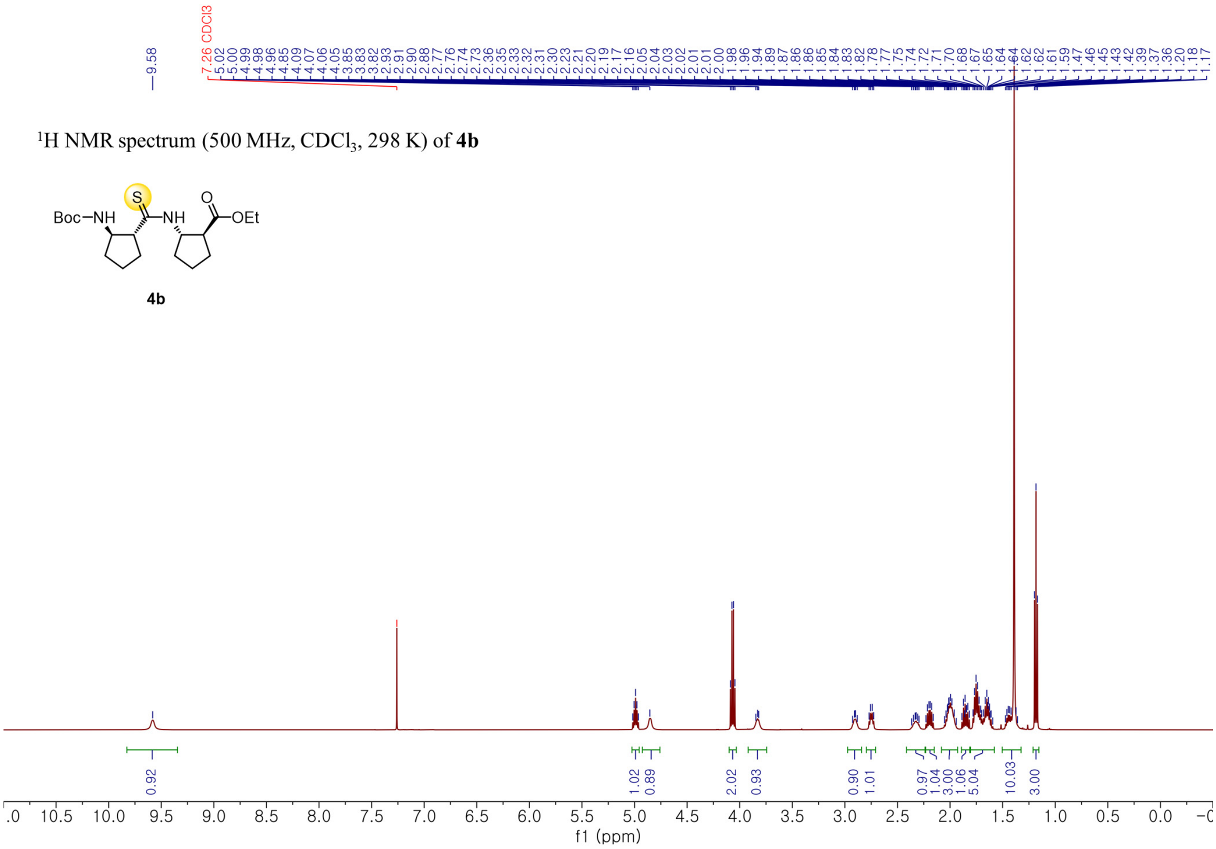

^{1}H NMR spectrum (500 MHz, $CDCl_3$, 298 K) of **4b**

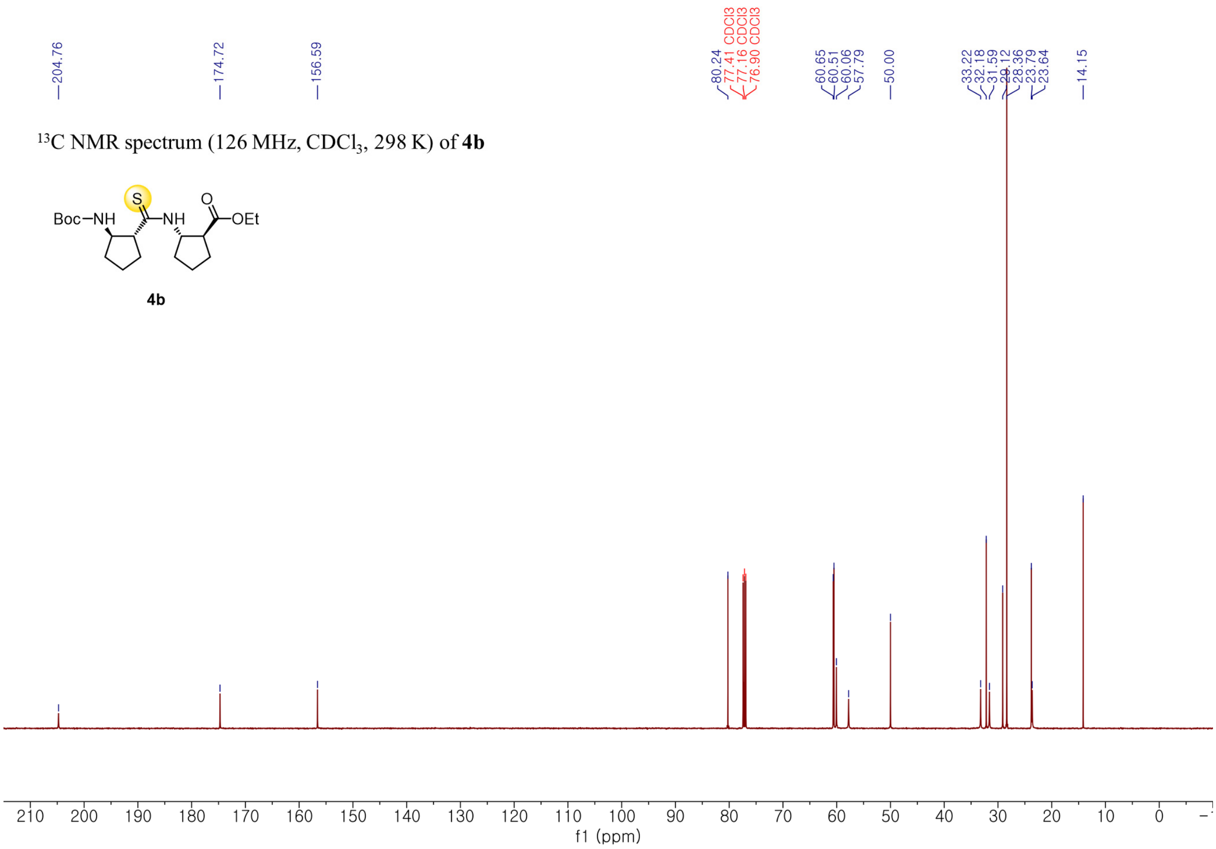

^{13}C NMR spectrum (126 MHz, $CDCl_3$, 298 K) of **4b**

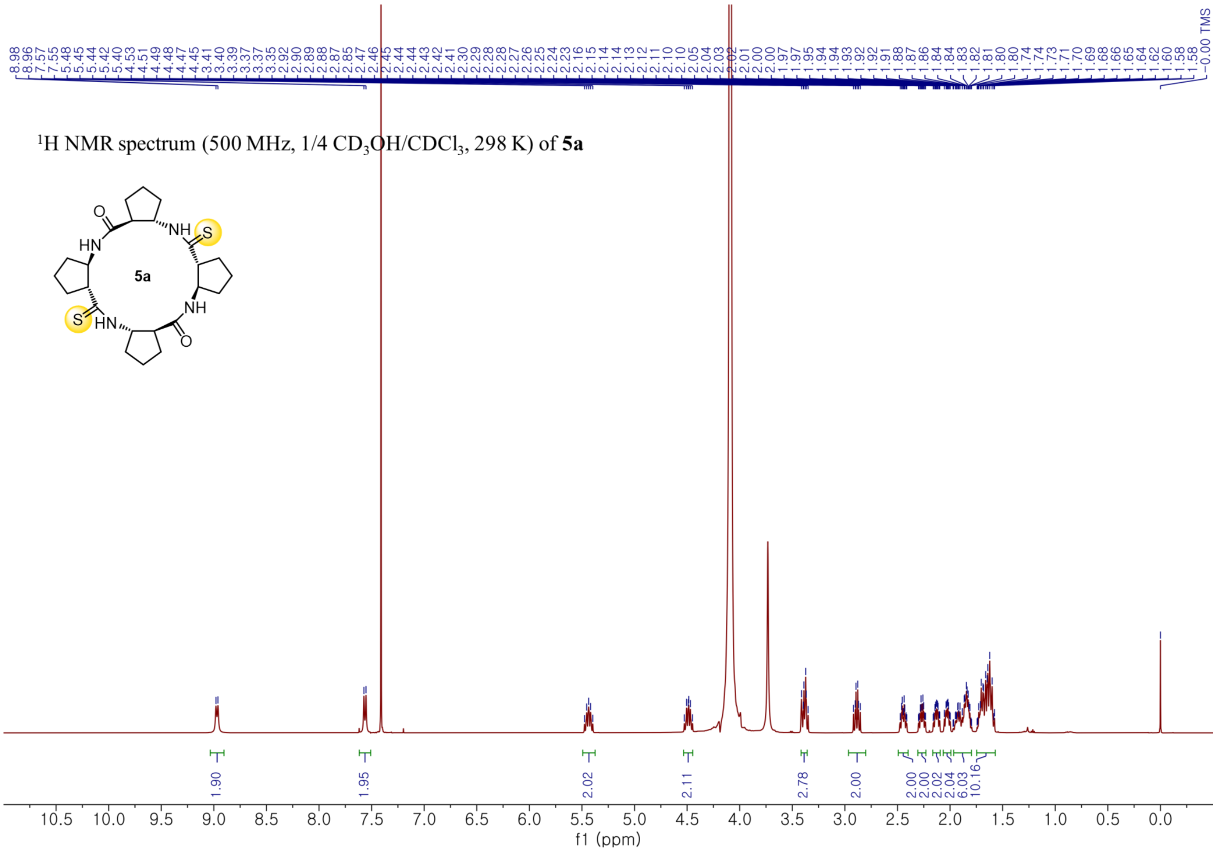

^{1}H NMR spectrum (500 MHz, 1/4 $CD_3OH/CDCl_3$, 298 K) of **5a**

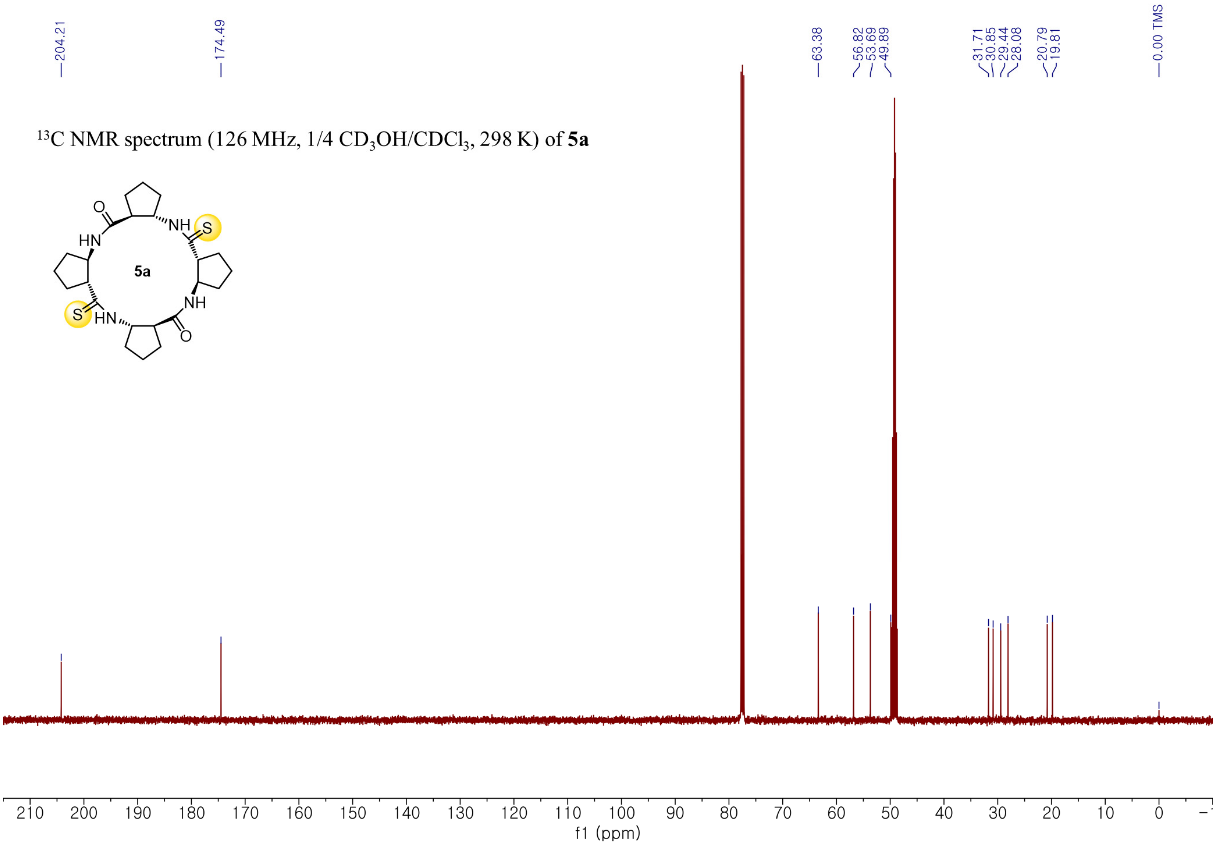

^{13}C NMR spectrum (126 MHz, 1/4 $CD_3OH/CDCl_3$, 298 K) of **5a**

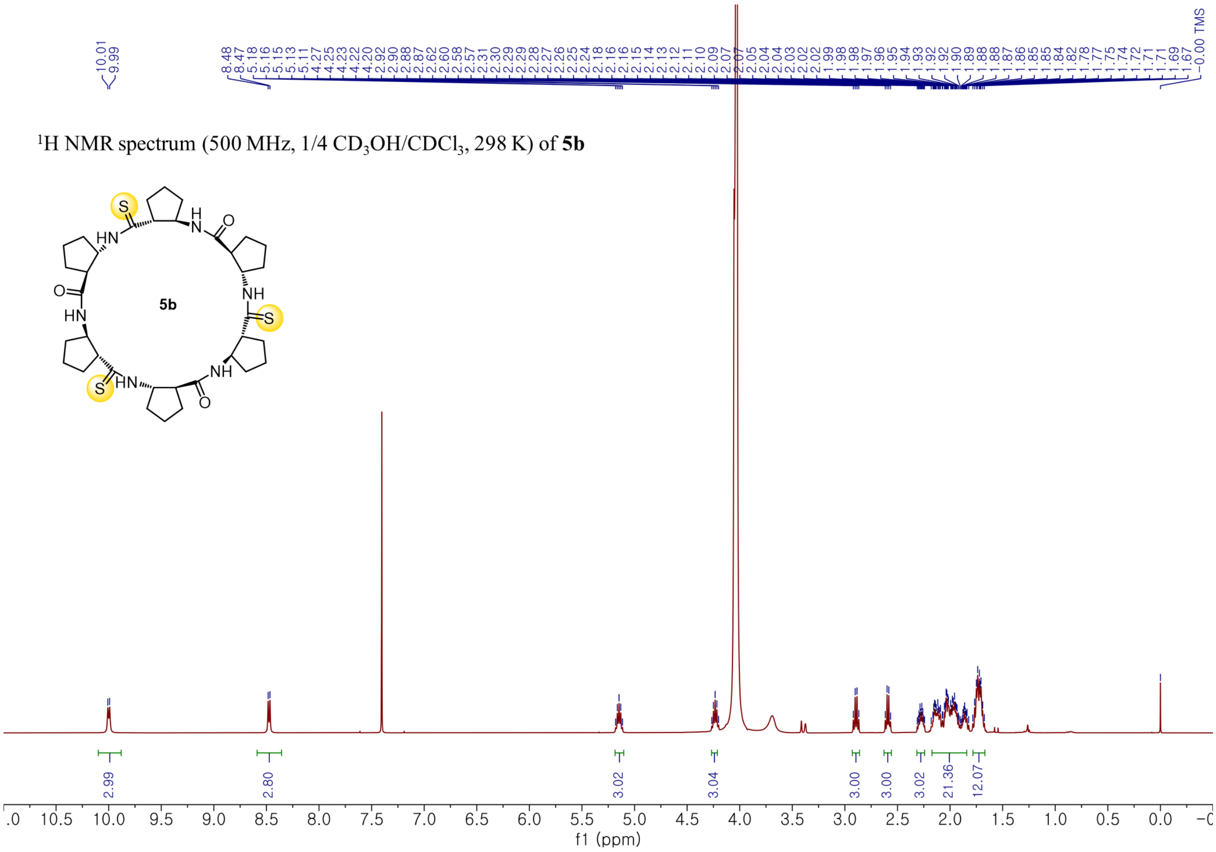

^{1}H NMR spectrum (500 MHz, 1/4 $CD_3OH/CDCl_3$, 298 K) of **5b**

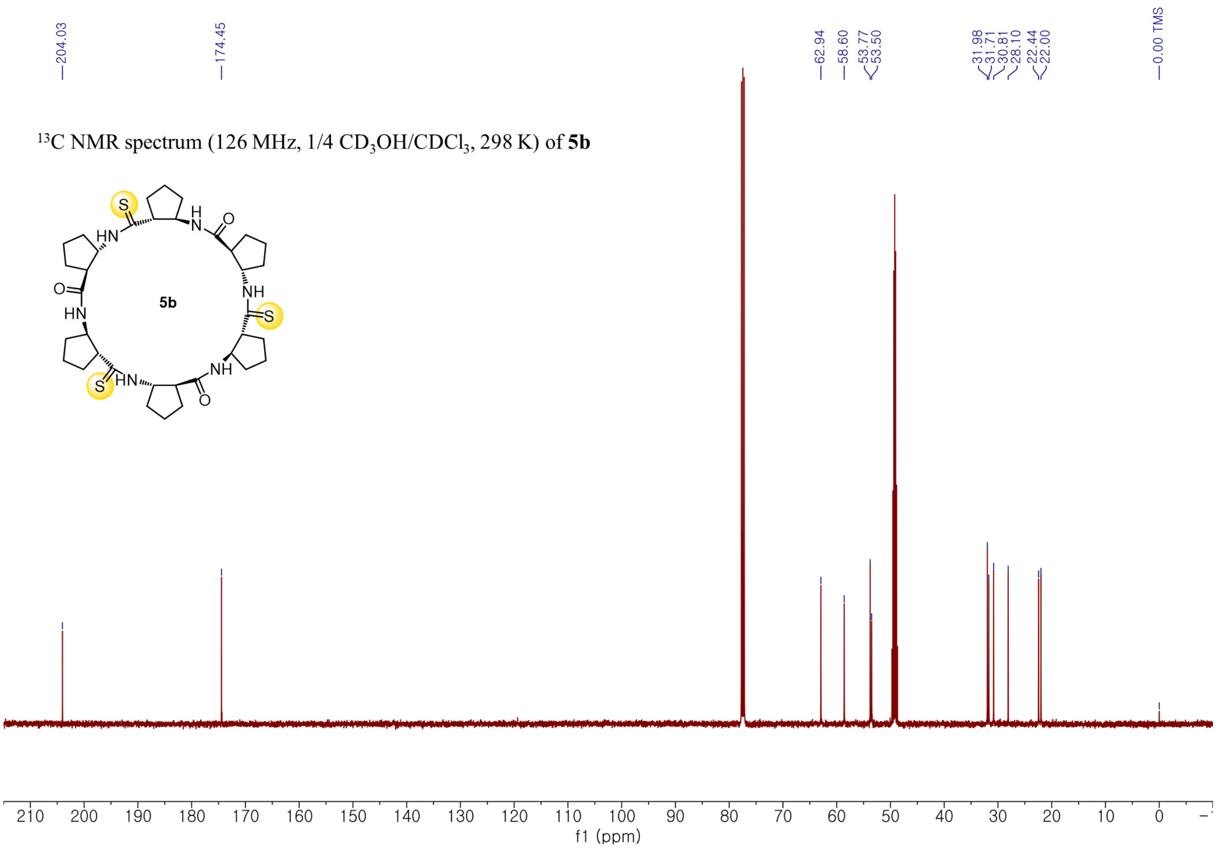

^{13}C NMR spectrum (126 MHz, 1/4 $CD_3OH/CDCl_3$, 298 K) of **5b**

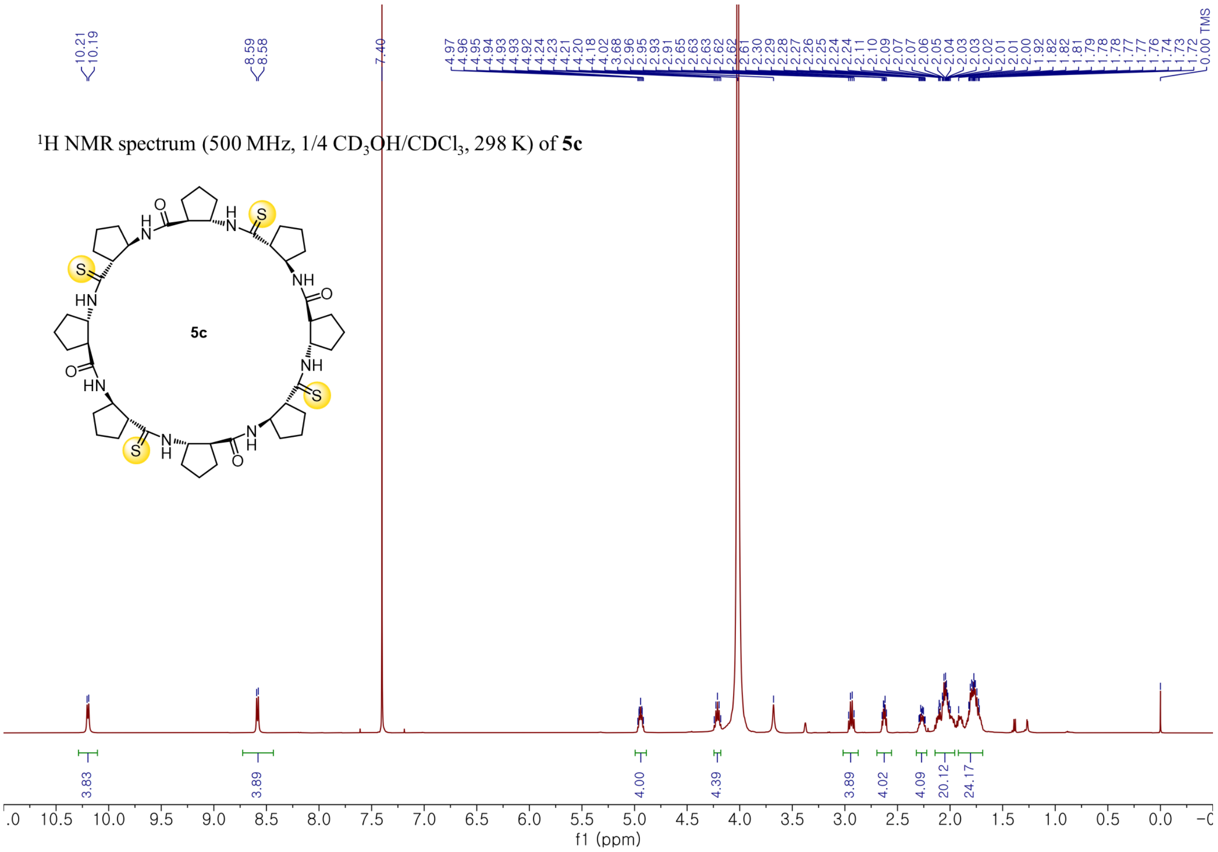

^{1}H NMR spectrum (500 MHz, 1/4 $CD_3OH/CDCl_3$, 298 K) of **5c**

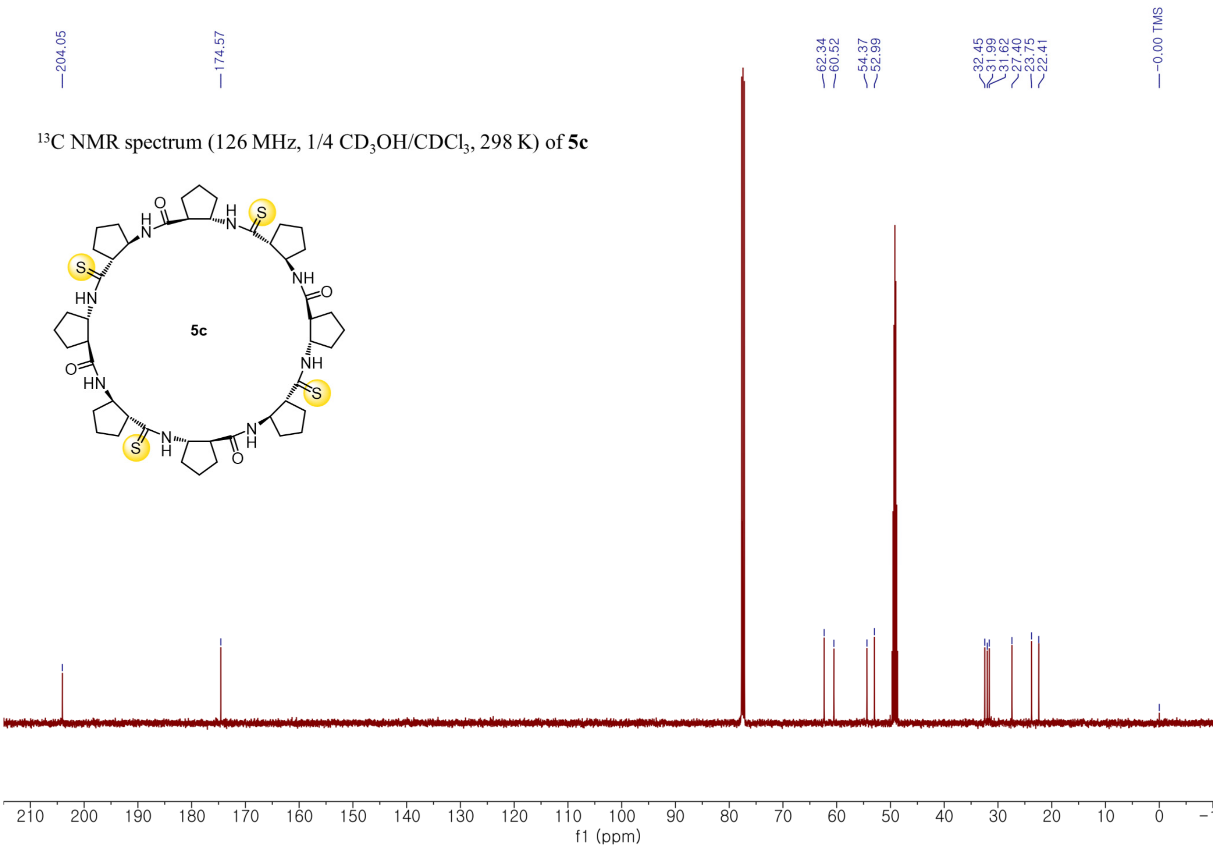

^{13}C NMR spectrum (126 MHz, 1/4 $CD_3OH/CDCl_3$, 298 K) of **5c**

^{1}H NMR spectrum (500 MHz, DMSO-d_6, 298 K) of **6**

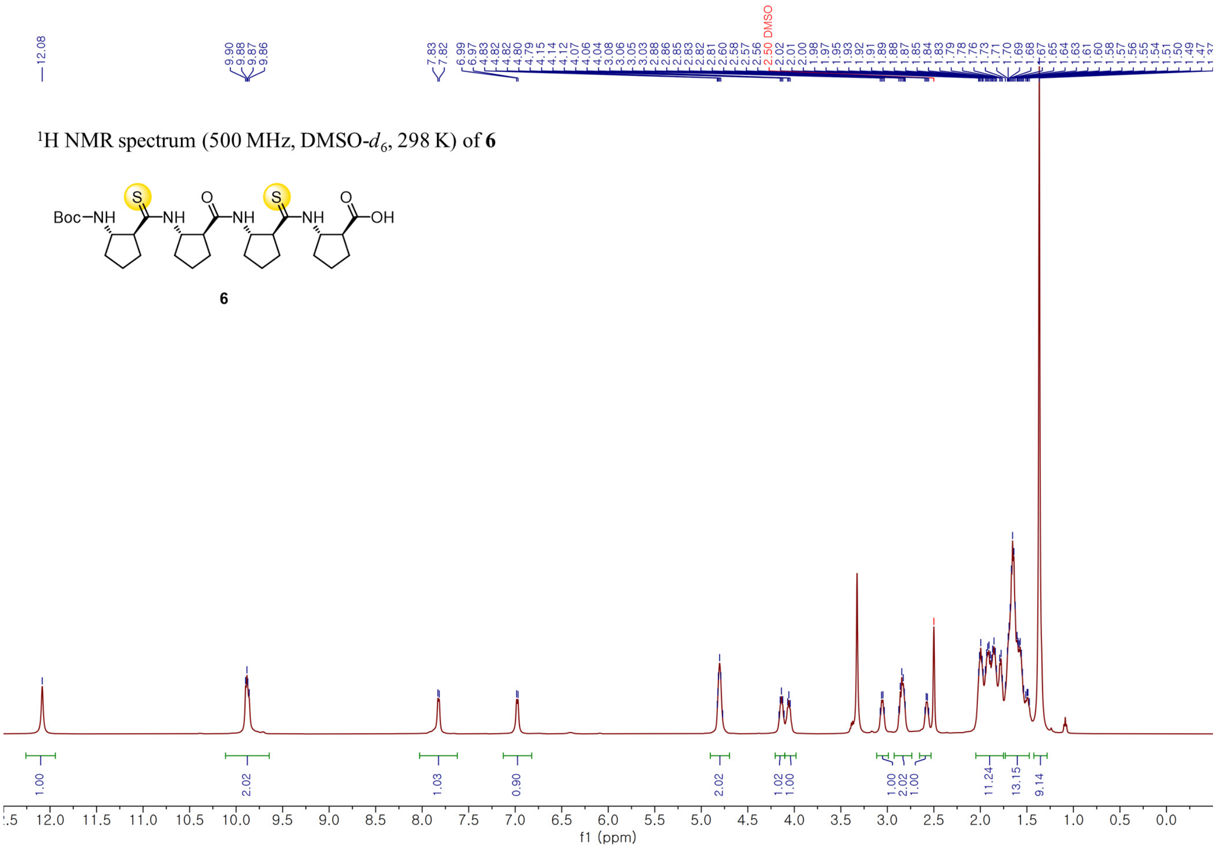

^{13}C NMR spectrum (126 MHz, DMSO-d_6, 298 K) of **6**

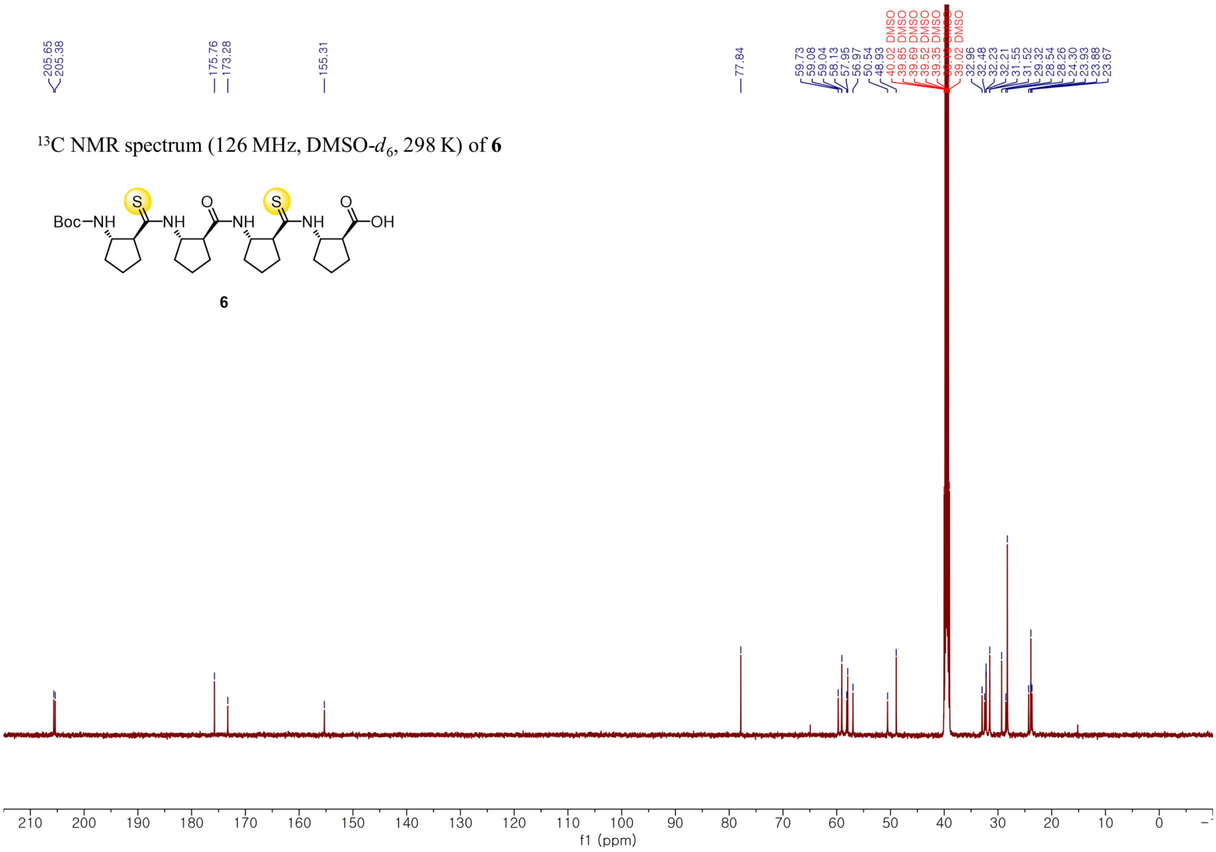

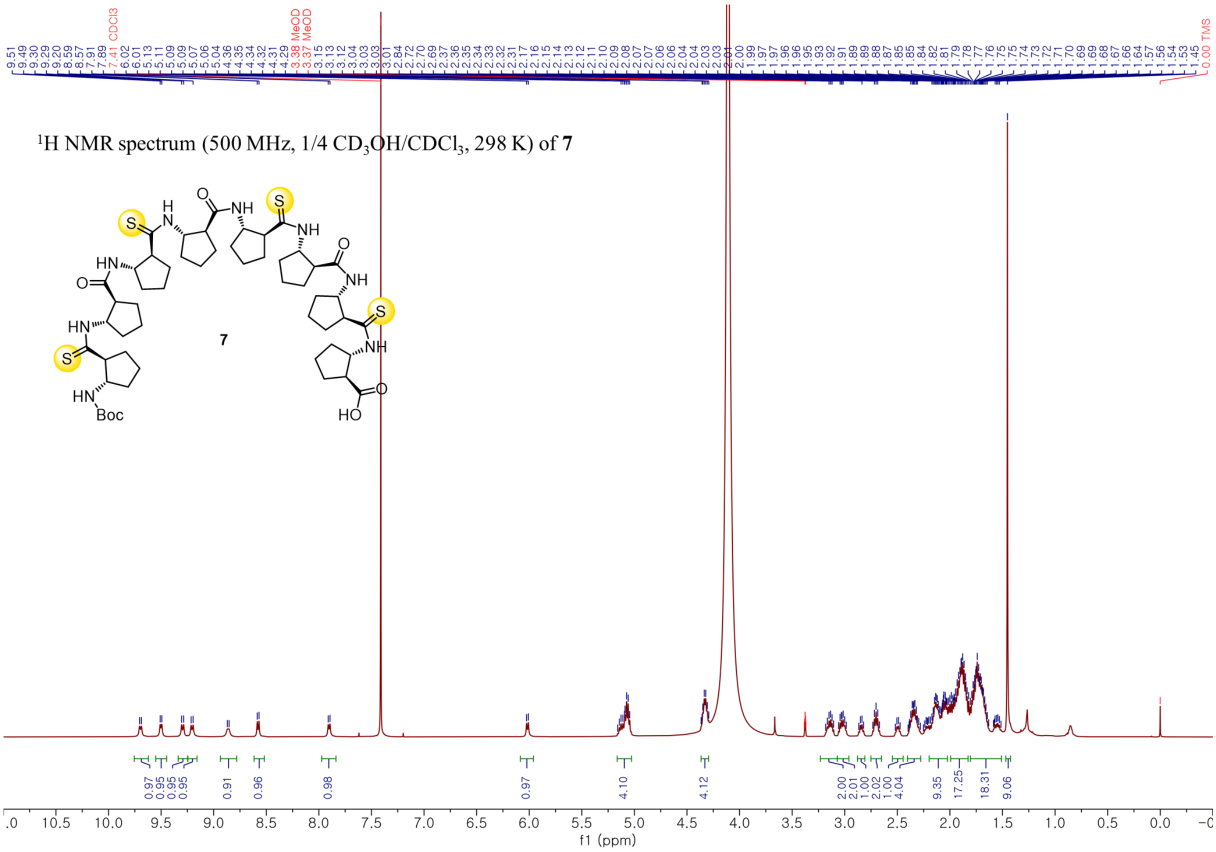

^1H NMR spectrum (500 MHz, 1/4 $CD_3OH/CDCl_3$, 298 K) of **7**

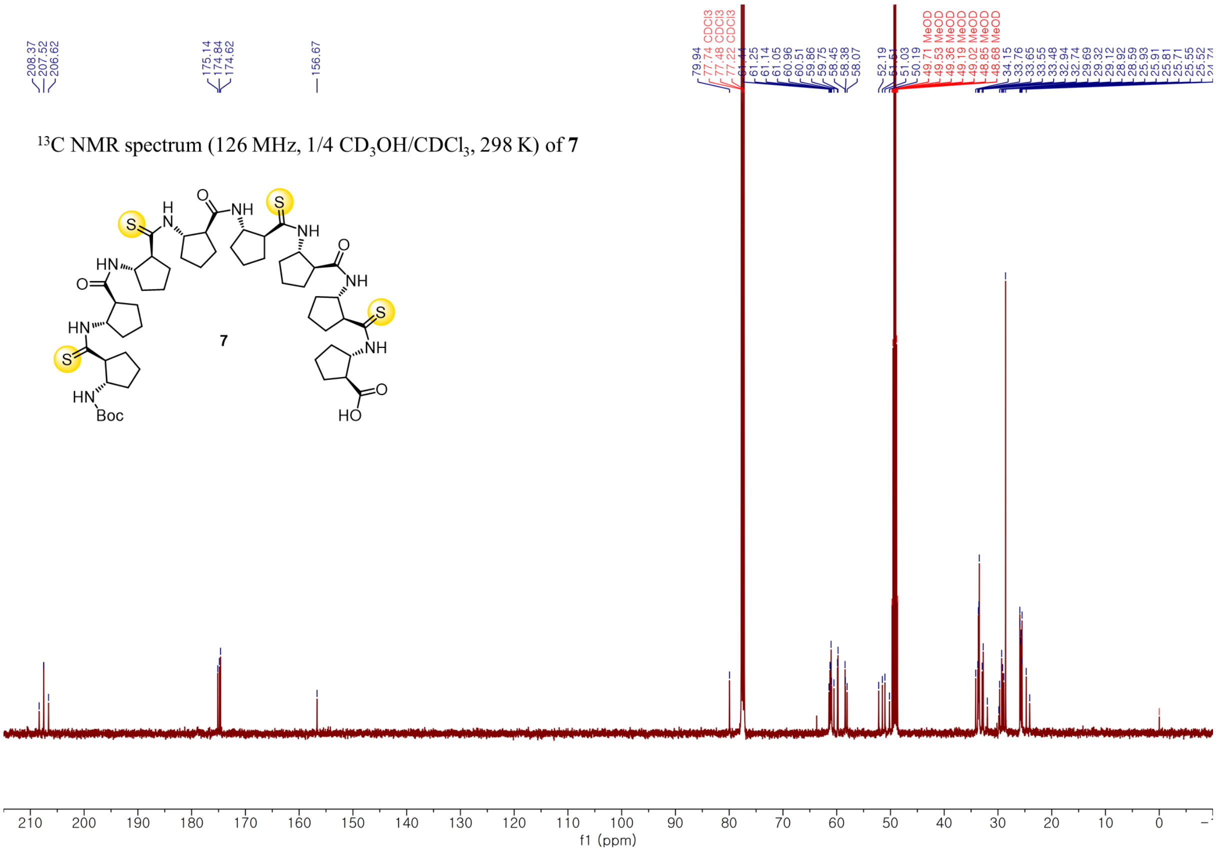

^{13}C NMR spectrum (126 MHz, 1/4 $CD_3OH/CDCl_3$, 298 K) of **7**

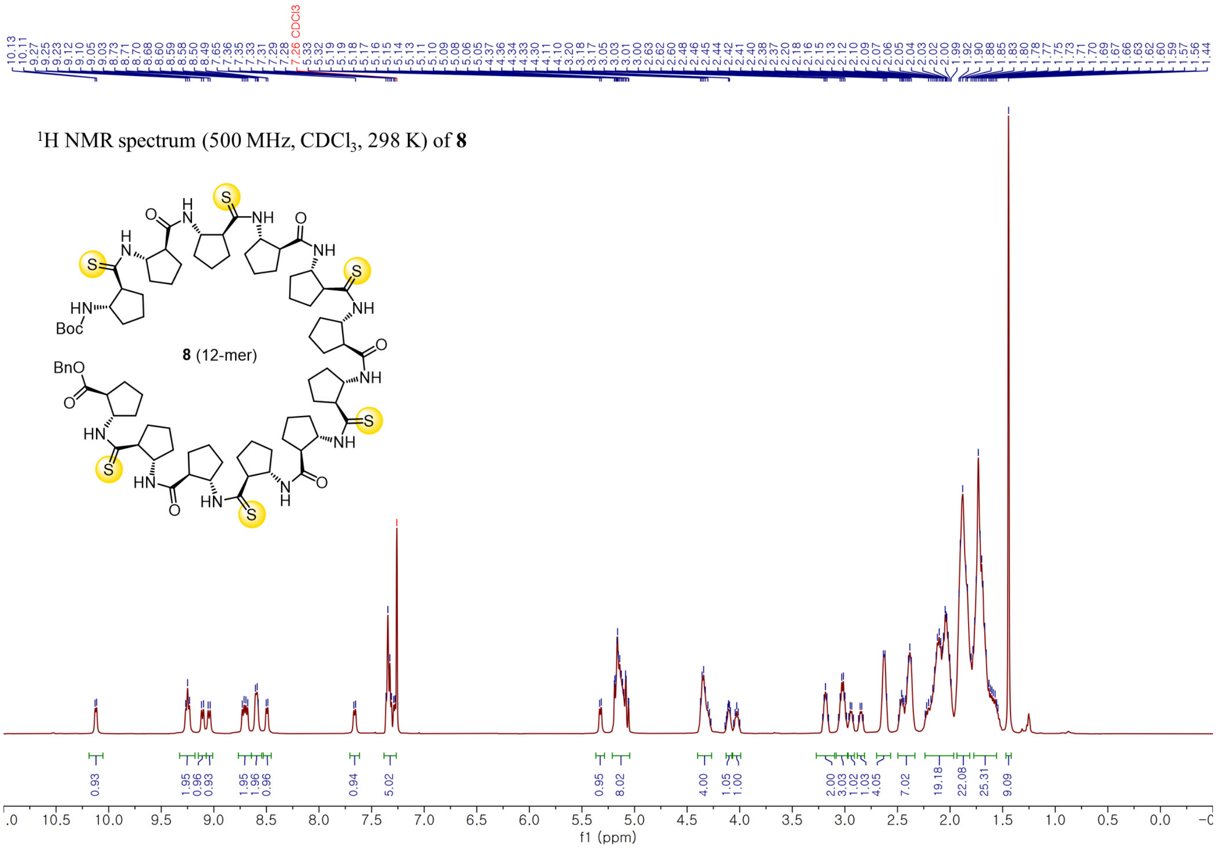

^{1}H NMR spectrum (500 MHz, $CDCl_3$, 298 K) of **8**

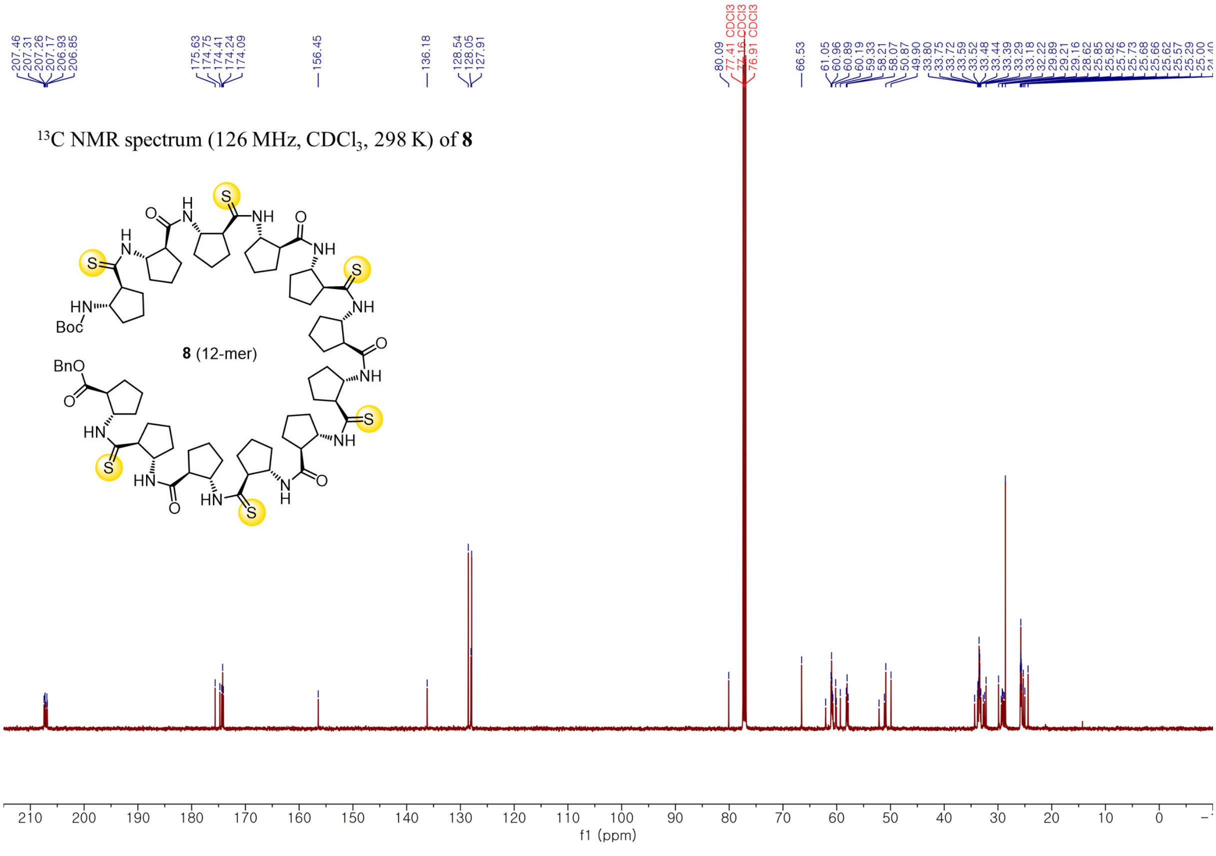

^{13}C NMR spectrum (126 MHz, $CDCl_3$, 298 K) of **8**

^{1}H NMR spectrum (500 MHz, $CDCl_3$, 298 K) of **9**

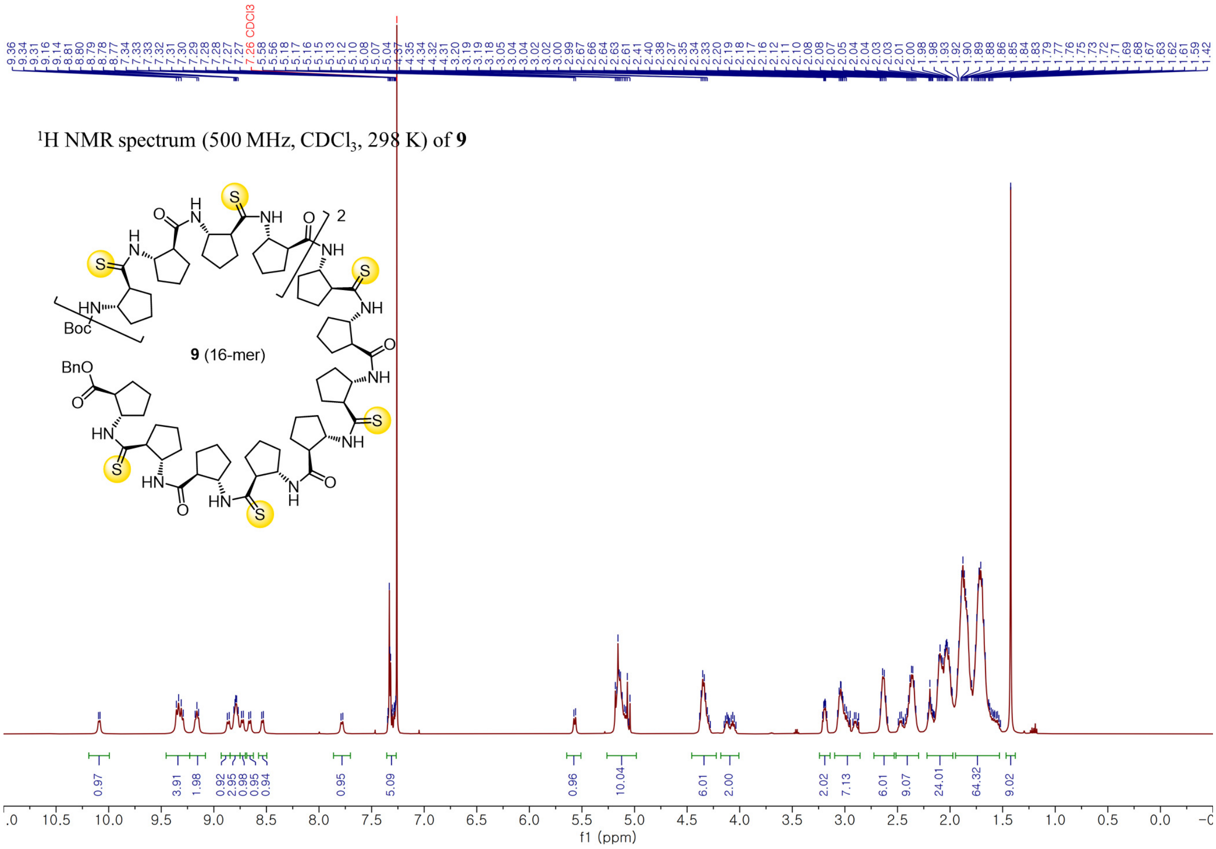

^{13}C NMR spectrum (126 MHz, $CDCl_3$, 298 K) of **9**

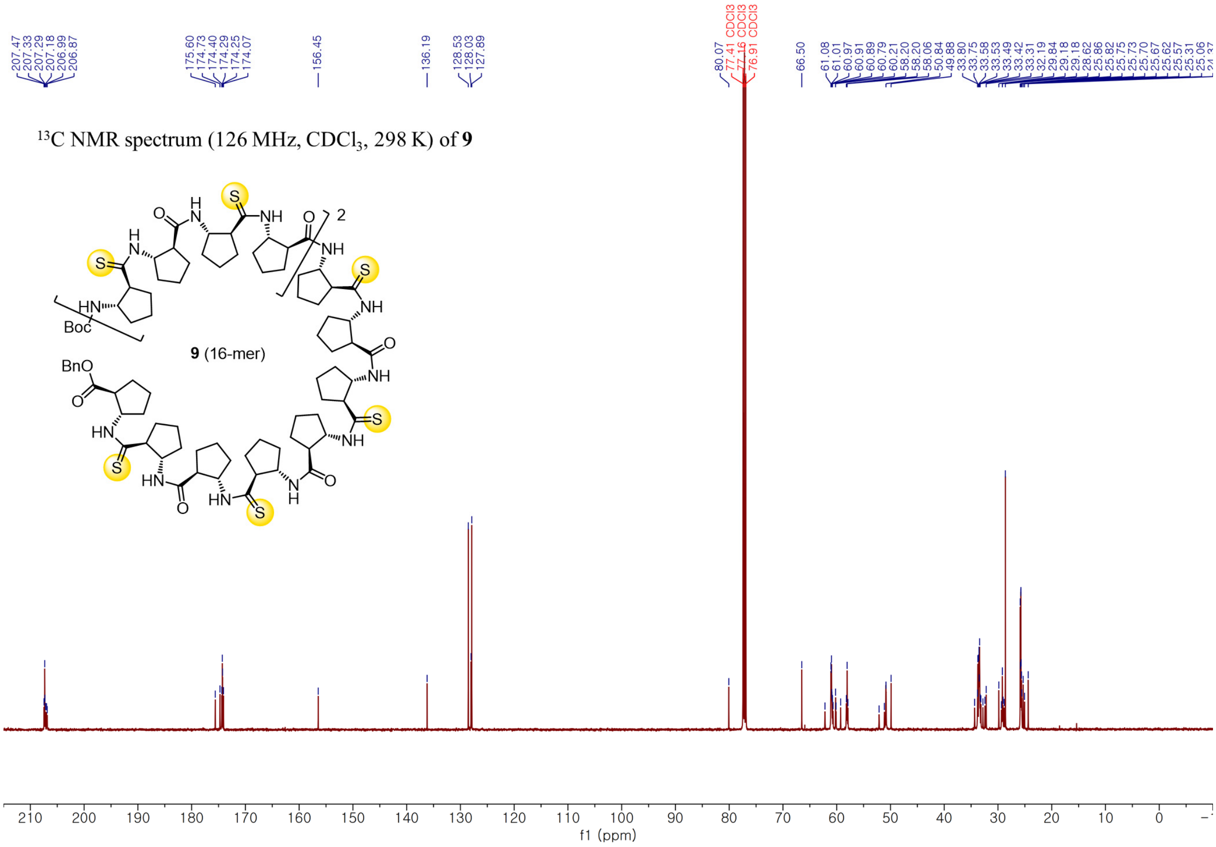

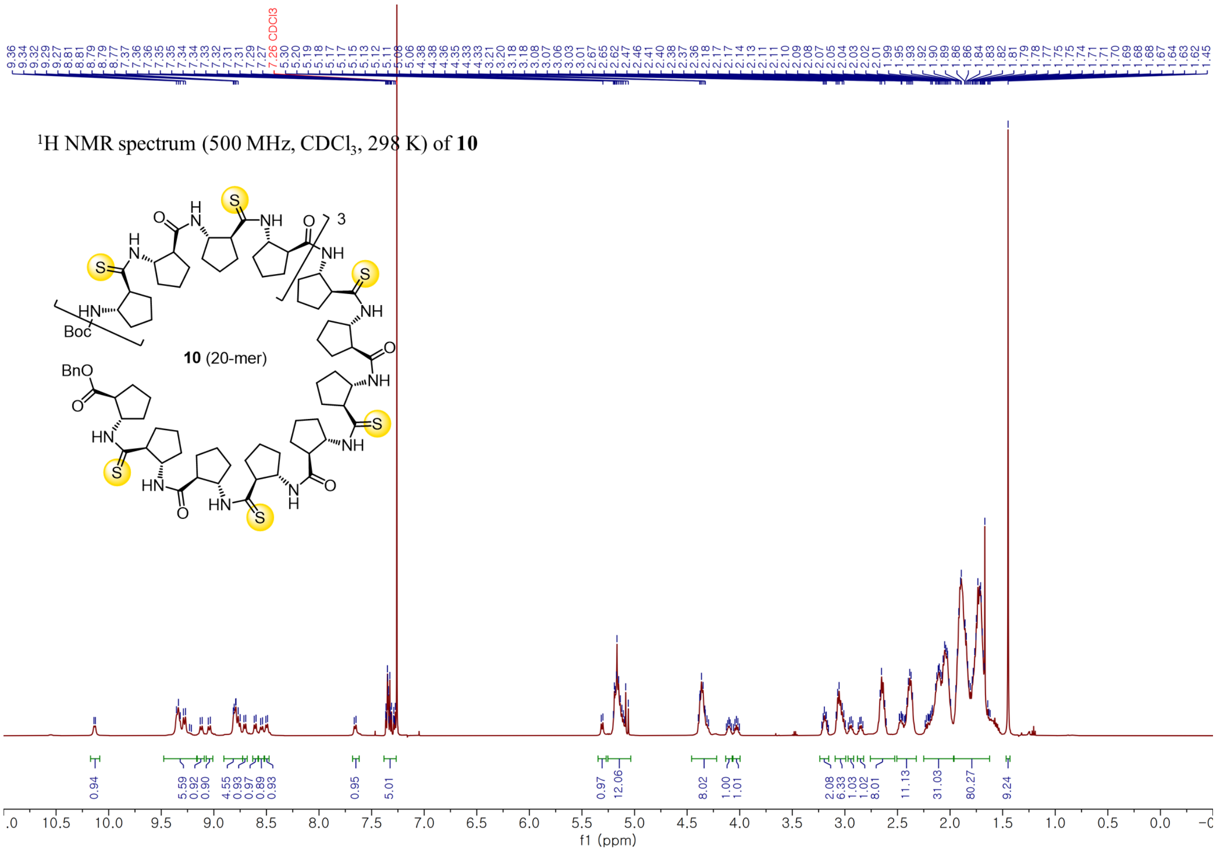

^{1}H NMR spectrum (500 MHz, $CDCl_3$, 298 K) of **10**

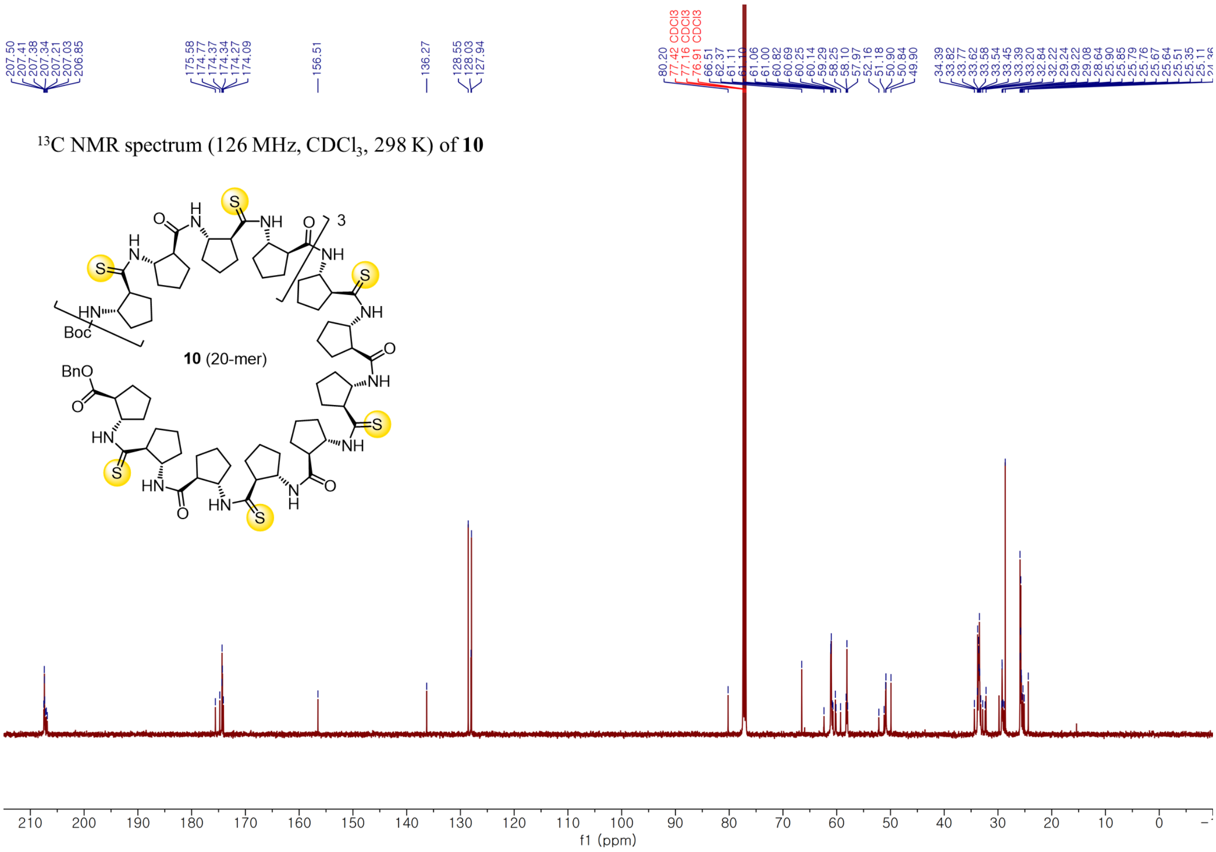

^{13}C NMR spectrum (126 MHz, $CDCl_3$, 298 K) of **10**

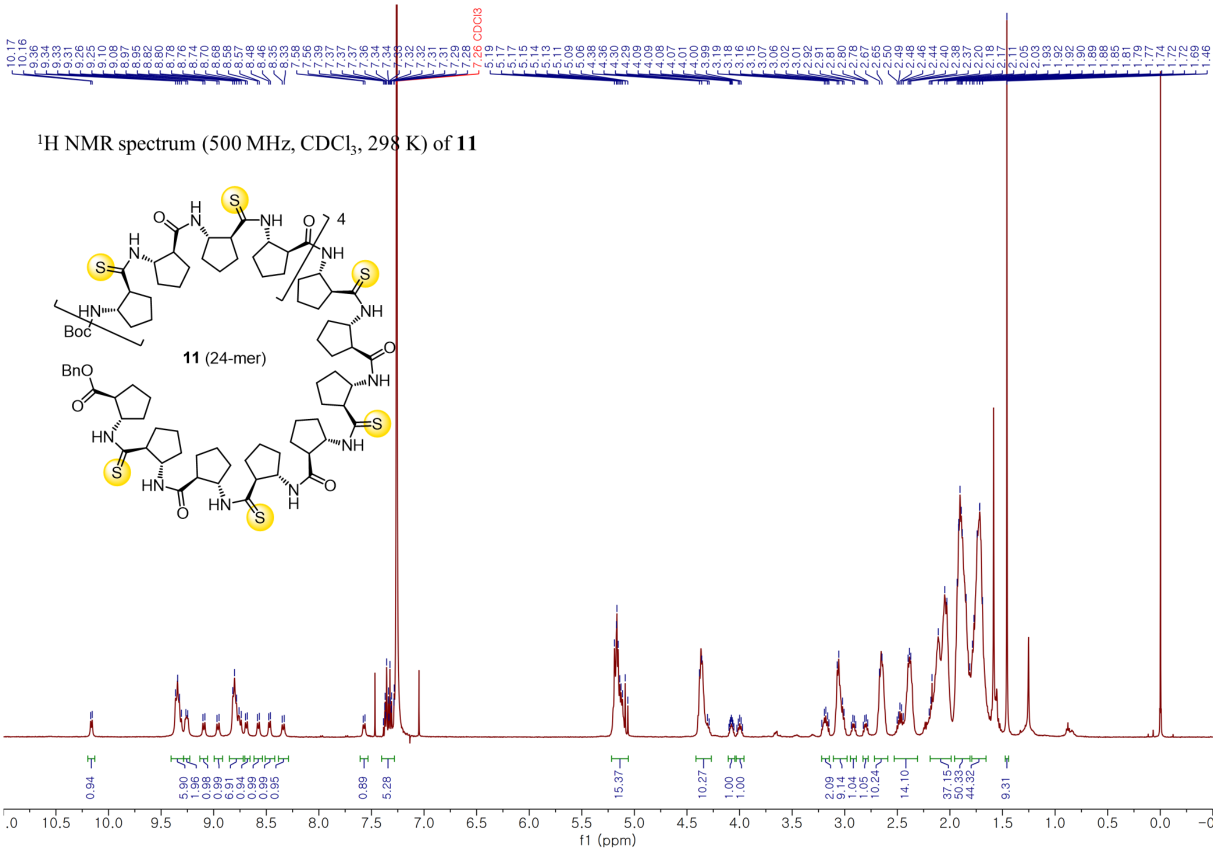

^{1}H NMR spectrum (500 MHz, $CDCl_3$, 298 K) of **11**

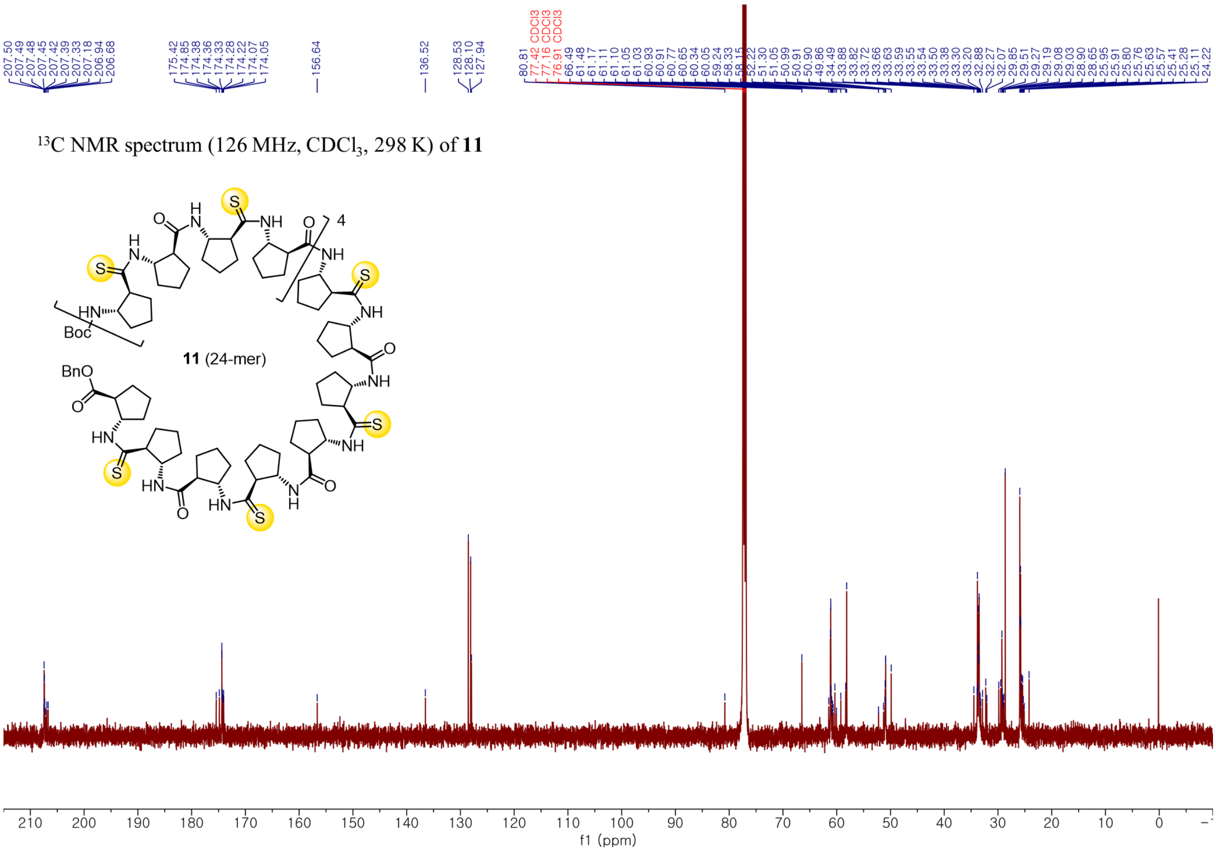

^{13}C NMR spectrum (126 MHz, $CDCl_3$, 298 K) of **11**

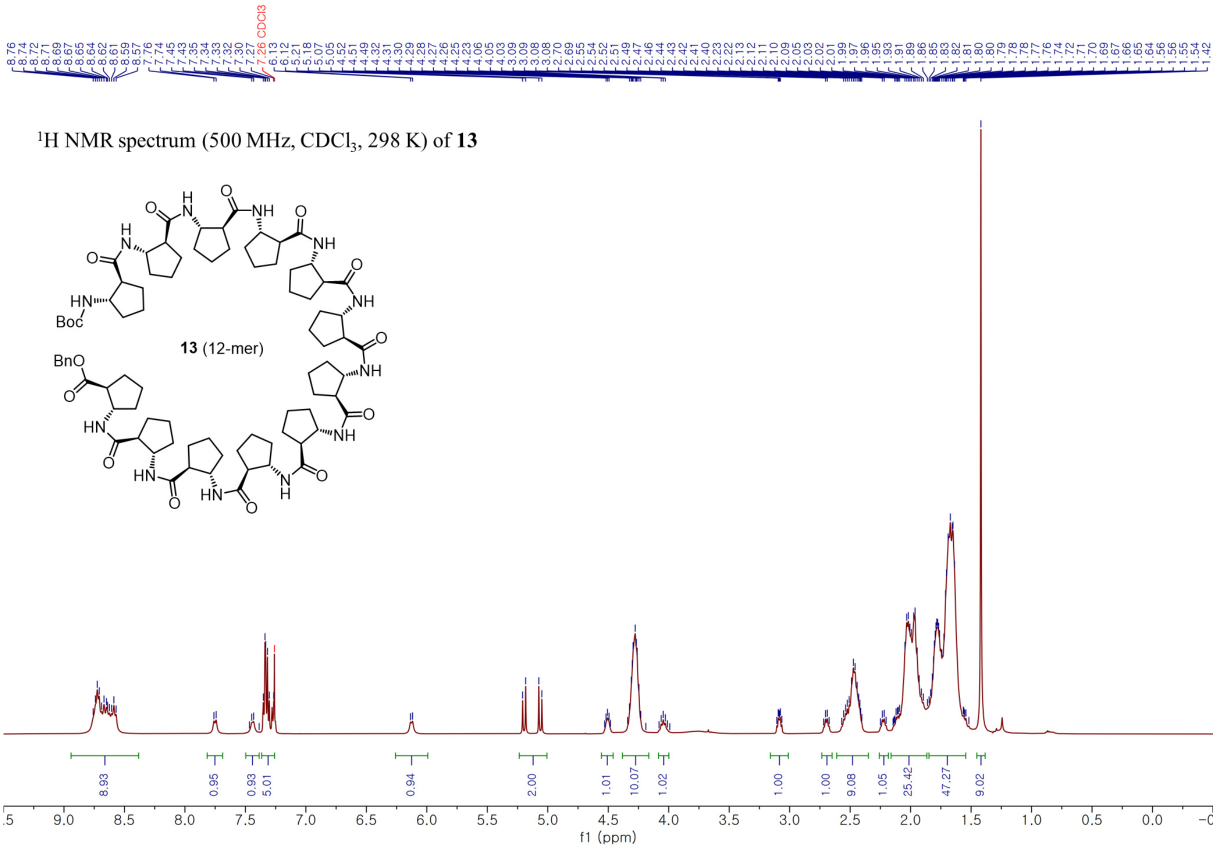

^{1}H NMR spectrum (500 MHz, $CDCl_3$, 298 K) of **13**

13 (12-mer)

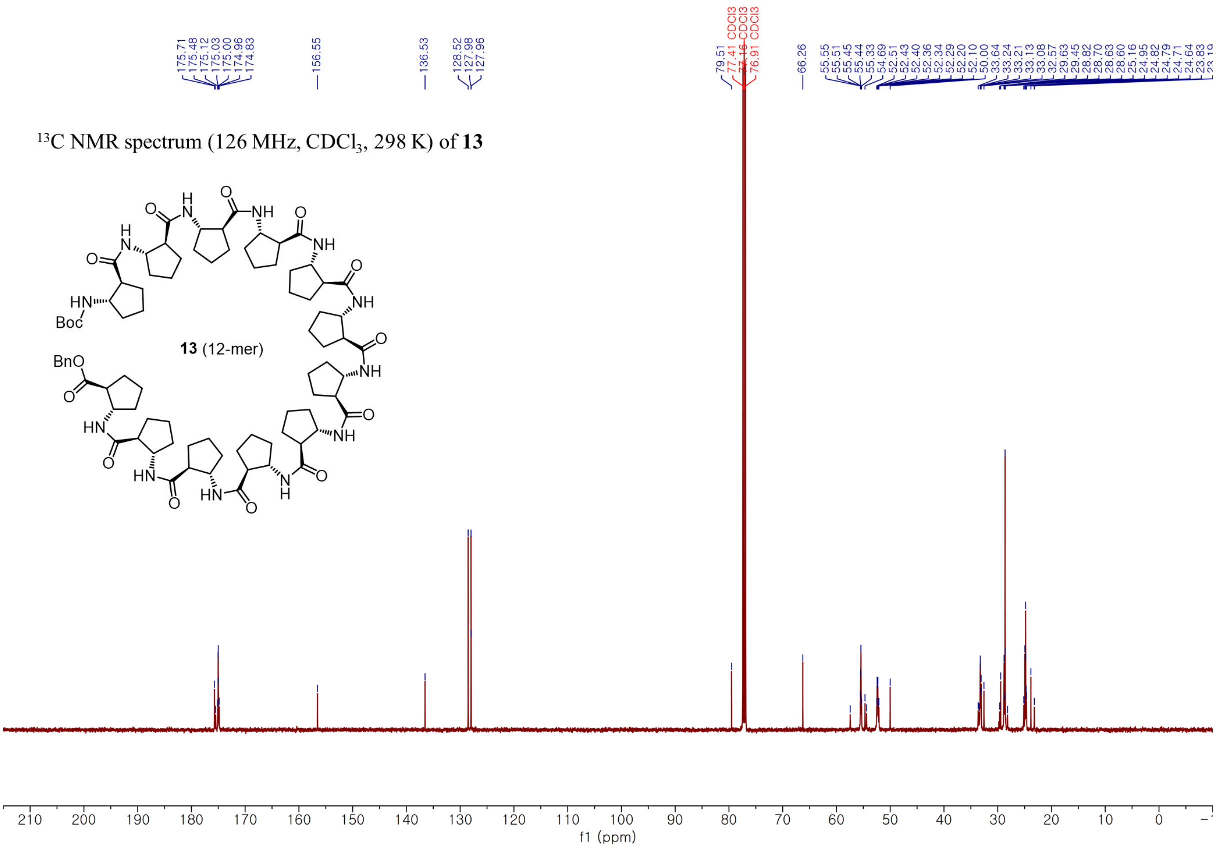

^{13}C NMR spectrum (126 MHz, $CDCl_3$, 298 K) of **13**

13 (12-mer)

^{1}H NMR spectrum (500 MHz, $CDCl_3$, 298 K) of **14**

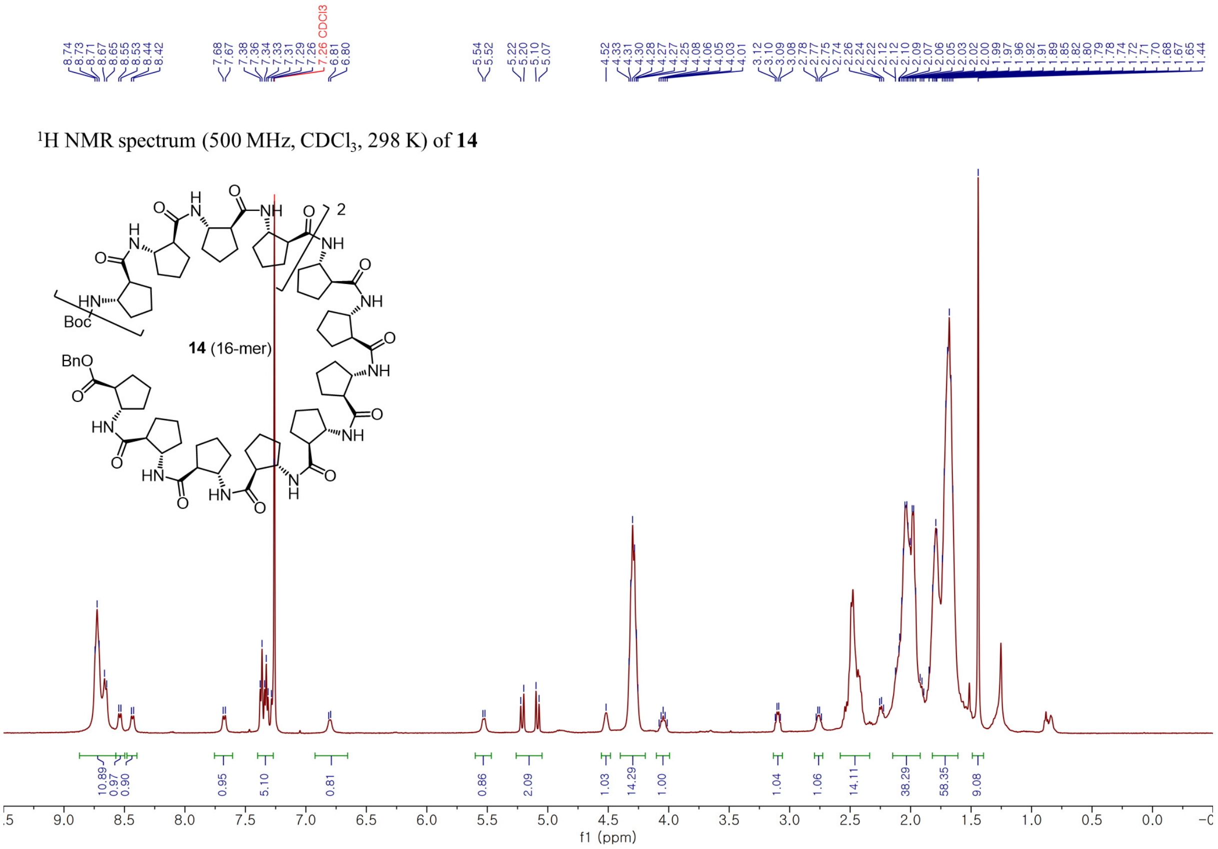

^{13}C NMR spectrum (126 MHz, $CDCl_3$, 298 K) of **14**

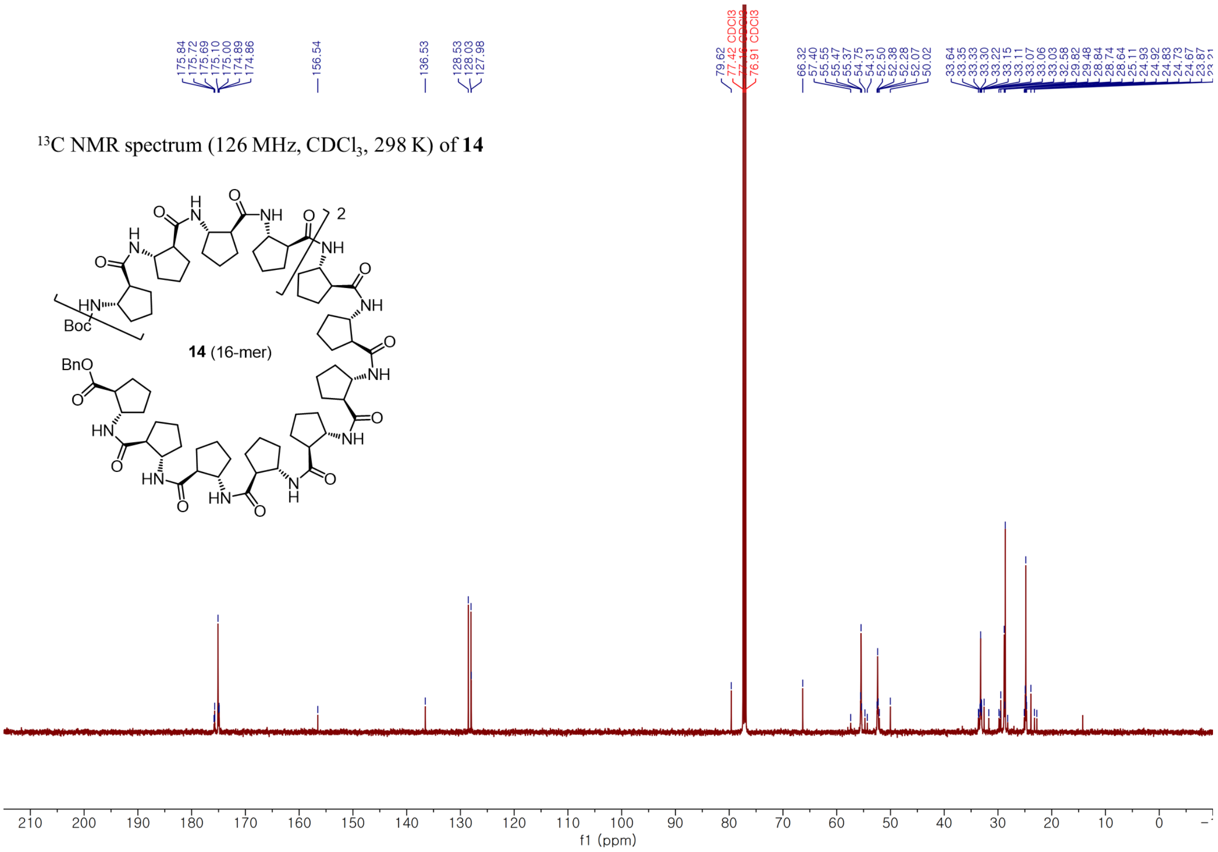

6.2. 2D NMR Spectra

2D NMR Spectra of **2b**

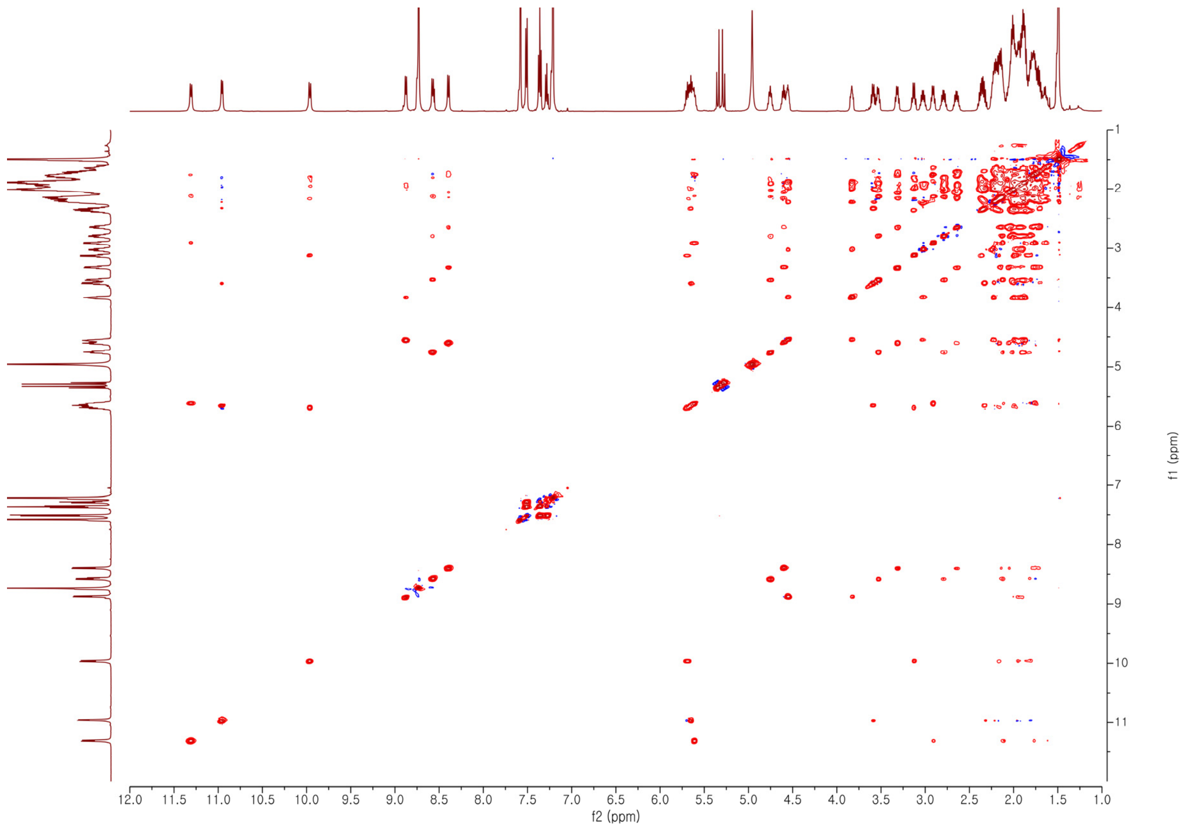

TOCSY NMR spectrum (500 MHz, pyridine-d_5) of **2b**.

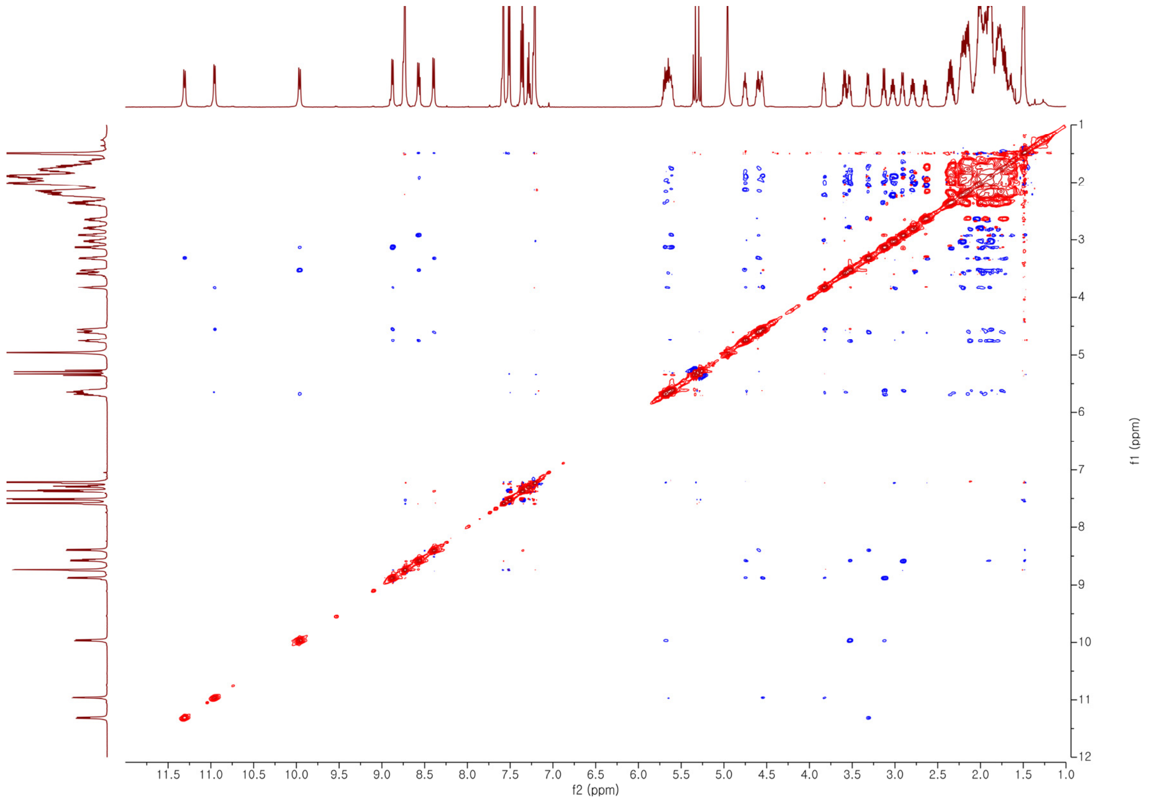

ROESY NMR spectrum (500 MHz, pyridine-d_5) of **2b**.

Chemical shift table for the backbone proton of **2b**.

	Boc	H_N	$H_β$	$H_α$	OBn
N-terminus	1.49	–	–	–	–
ACPC(1)	–	8.46	4.60	3.32	–
ACPC(2)	–	11.31	5.61	2.91	–
ACPC(3)	–	8.57	4.75	3.53	–
ACPC(4)	–	9.96	5.69	3.13	–
ACPC(5)	–	8.88	4.55	3.83	–
ACPC(6)	–	10.96	5.65	3.59	–
C-terminus	–	–	–	–	5.31

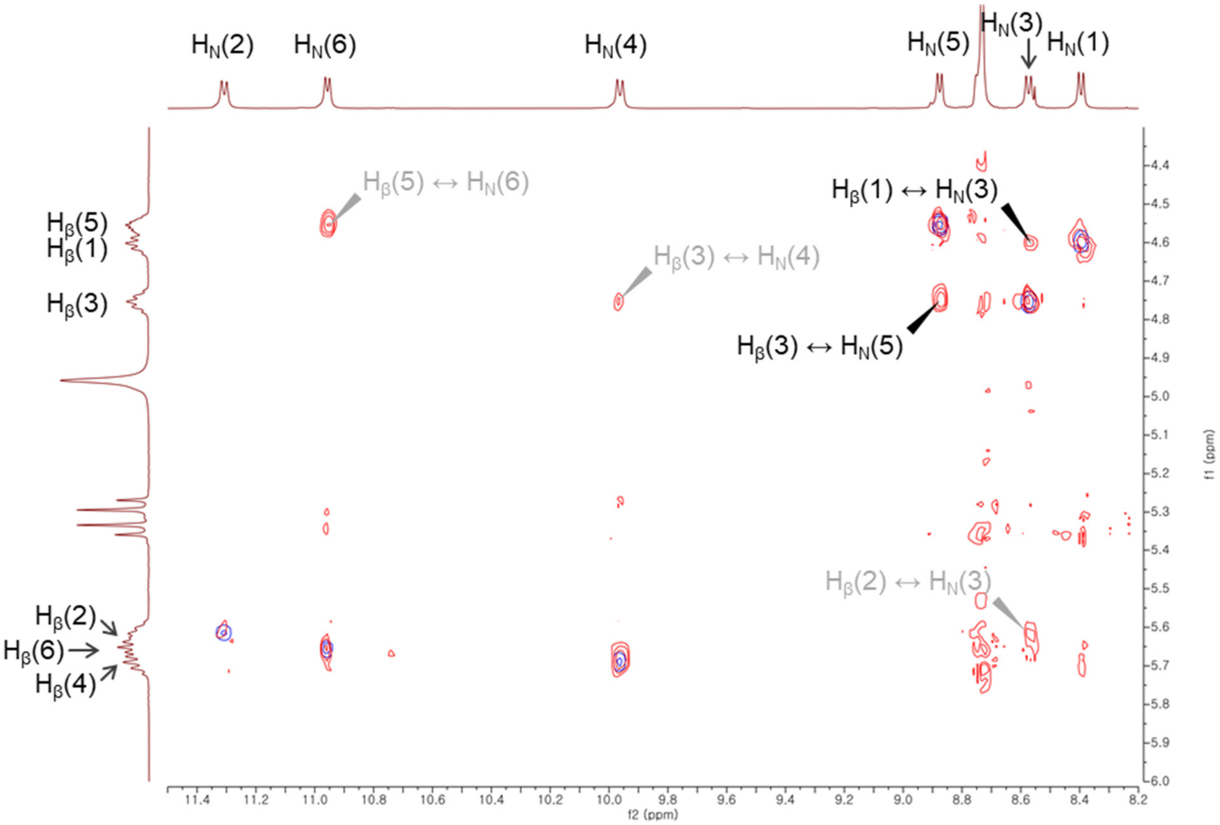

Superimposed partial ($H_β$ ↔ H_N region) TOCSY (blue) and ROESY (red) spectra (500 MHz, pyridine-d_5) of **2b**. Non-$H_β(i)$↔$H_N(i+2)$ NOEs were noted as grey. The $H_β(2)$ ↔ $H_N(4)$ and $H_β(4)$ ↔ $H_N(6)$ NOE cross-peaks were not observed.

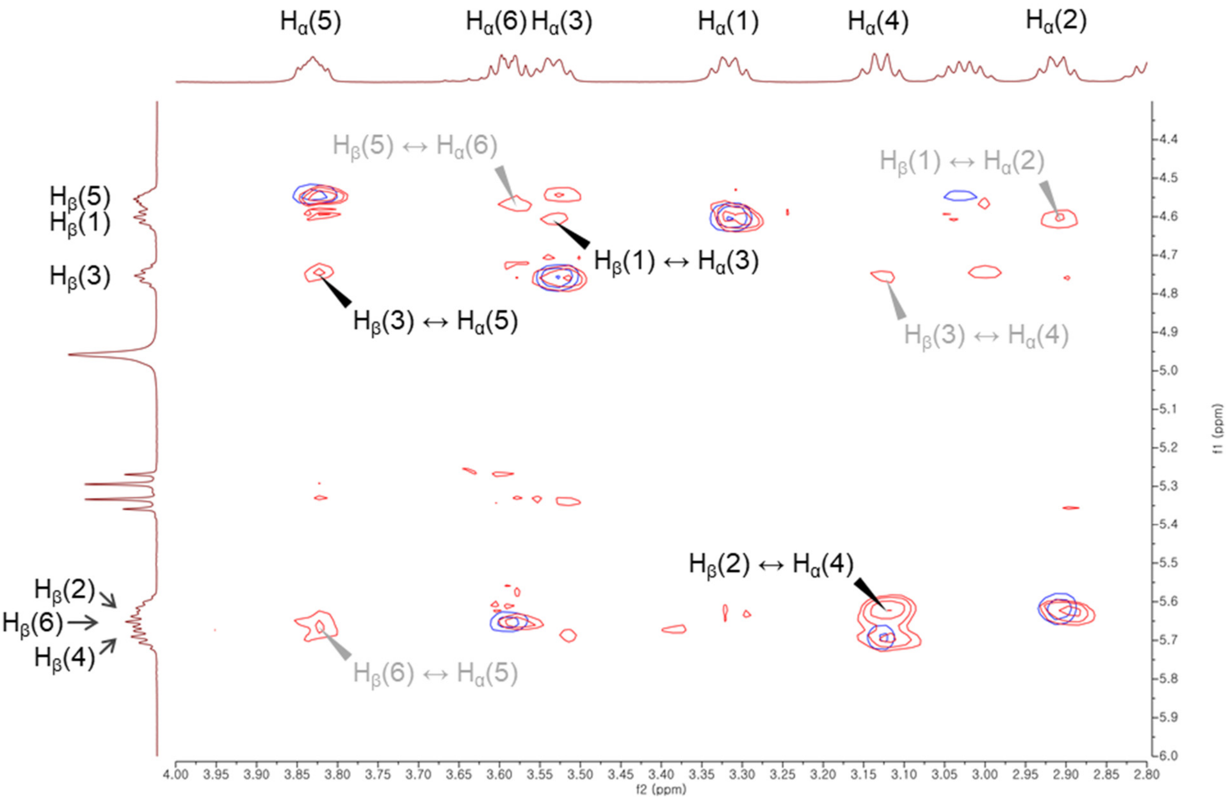

Superimposed partial ($H_\beta \leftrightarrow H_\alpha$ region) TOCSY (blue) and ROESY (red) spectra (500 MHz, pyridine-d_5) of **2b**. Non-$H_\beta(i) \leftrightarrow H_\alpha(i+2)$ NOEs were noted as grey. The $H_\beta(4) \leftrightarrow H_\alpha(6)$ NOE cross-peak was not observed.

7. References

(1) Otwinowski, Z.; Minor, W. Processing of X-ray diffraction data collected in oscillation mode. *Methods in Enzymology*; Vol 276; Academic Press, 1997; pp 307–326. DOI: 10.1016/S0076-6879(97)76066-X

(2) Minor, W.; Cymborowski, M; Otwinowski, Z.; Chruszcz, M. *HKL*-3000: the integration of data reduction and structure solution – from diffraction images to an initial model in minutes. *Acta Cryst. D* **2006**, *62* (8), 859–866. DOI: 10.1107/S0907444906019949

(3) Sheldrick, G. M. *SHELXTL-97.*

(4) Sheldrick, G. M. *SHELXT* – Integrated space-group and crystal-structure determination. *Acta Cryst. A* **2015**, *71* (1), 3–8. DOI: 10.1107/S2053273314026370

(5) Sheldrick, G. M. Crystal structure refinement with *SHELXL*. *Acta Cryst. C* **2015**, *71* (1), 3–8. DOI: 10.1107/S2053229614024218

(6) Bourhis, L. J.; Dolomanov, O. V.; Gildea, R. J.; Howard, J. A. K.; Puschmann, H. The anatomy of a comprehensive constrained, restrained refinement program for the modern computing environment – *Olex2* dissected. *Acta Cryst. A* **2015**, *71* (1), 59–75. DOI: 10.1107/S2053273314022207

(7) Dolomanov, O. V.; Bourhis, L. J.; Gildea, R. J.; Howard, J. A. K.; Puschmann, H. *OLEX2*: a complete structure solution, refinement and analysis program. *J. Appl. Cryst.* **2009**, *42* (2), 339–341. DOI: 10.1107/S0021889808042726

(8) Hong, J.; Lee, W.; Lee, H.-S. Optimized stereoselective and scalable synthesis of five-membered cyclic *trans*-β-amino acid building blocks via reductive amination. *Bull. Korean Chem. Soc.* **2023**, *44* (12), 1034–1039. DOI: 10.1002/bkcs.12786

(9) Appella, D. H.; Christianson, L. A.; Klein, D. A.; Richards, M. R.; Powell, D. R.; Gellman, S. H. Synthesis and Structural Characterization of Helix-Forming β-Peptides: *trans*-2-Aminocyclopentanecarboxylic Acid Oligomers. *J. Am. Chem. Soc.* **1999**, *121* (33), 7574–7581. DOI: 10.1021/ja991185g

(10) Kim, J.; Kwon, S.; Kim, S. H.; Lee, C.-K.; Lee, J.-H.; Cho, S. J.; Lee, H.-S.; Lhee, H. Microtubes with Rectangular Cross-Section by Self-Assembly of a Short β-Peptide Foldamer. *J. Am. Chem. Soc.* **2012**, *134* (51), 20573–20576. DOI: 10.1021/ja3088482
